# Placental endocrine insufficiency programs anxiety, deficits in cognition and atypical social behaviour in offspring

**DOI:** 10.1093/hmg/ddab154

**Published:** 2021-06-07

**Authors:** David J Harrison, Hugo D J Creeth, Hannah R Tyson, Raquel Boque-Sastre, Susan Hunter, Dominic M Dwyer, Anthony R Isles, Rosalind M John

**Affiliations:** Biomedicine Division, School of Biosciences, Cardiff University, Cardif CF10 3AX, UK; Biomedicine Division, School of Biosciences, Cardiff University, Cardif CF10 3AX, UK; Biomedicine Division, School of Biosciences, Cardiff University, Cardif CF10 3AX, UK; Biomedicine Division, School of Biosciences, Cardiff University, Cardif CF10 3AX, UK; Biomedicine Division, School of Biosciences, Cardiff University, Cardif CF10 3AX, UK; School of Psychology, Cardiff University, Cardiff CF10 3AX, UK; Behavioural Genetics Group, MRC Centre for Neuropsychiatric Genetics and Genomics, Neuroscience and Mental Health Research Institute, Cardiff University, Cardiff CF24 4HQ, UK; Biomedicine Division, School of Biosciences, Cardiff University, Cardif CF10 3AX, UK

## Abstract

Abnormally elevated expression of the imprinted *PHLDA2* gene has been reported in the placenta of human babies that are growth restricted *in utero* in several studies. We previously modelled this gene alteration in mice and found that just 2-fold increased expression of *Phlda2* resulted in placental endocrine insufficiency. In addition, elevated *Phlda2* was found to drive fetal growth restriction (FGR) of transgenic offspring and impaired maternal care by their wildtype mothers. Being born small and being exposed to suboptimal maternal care have both been associated with the increased risk of mental health disorders in human populations. In the current study we probed behavioural consequences of elevated *Phlda2* for the offspring. We discovered increased anxiety-like behaviours, deficits in cognition and atypical social behaviours, with the greatest impact on male offspring. Subsequent analysis revealed alterations in the transcriptome of the adult offspring hippocampus, hypothalamus and amygdala, regions consistent with these behavioural observations. The inclusion of a group of fully wildtype controls raised in a normal maternal environment allowed us to attribute behavioural and molecular alterations to the adverse maternal environment induced by placental endocrine insufficiency rather than the specific gene change of elevated *Phlda2*. Our work demonstrates that a highly common alteration reported in human FGR is associated with negative behavioural outcomes later in life. Importantly, we also establish the experimental paradigm that placental endocrine insufficiency can program atypical behaviour in offspring highlighting the under-appreciated role of placental endocrine insufficiency in driving disorders of later life behaviour.

## Introduction

Fetal growth restriction (FGR) is a condition where the baby’s growth slows *in utero* resulting in birth weight significantly lower than normal (1). FGR is a major threat to human health because of the impact on short-term survival and the longer term impact on the mental and metabolic health of survivors, a phenomenon described as fetal programming or developmental origins of disease (2–6). Specifically, FGR infants are at increased risk of neurological disorders including attention deficit/hyperactivity disorder (ADHD), anxiety, depression and schizophrenia (7–10). It is therefore imperative that we achieve a greater understanding of the causes and consequence of FGR.

Aberrant expression of *PLECKSTRIN HOMOLOGY-LIKE DOMAIN FAMILY A MEMBER 2* (*PHLDA2*) in the placenta has been reported in a number of studies of FGR, fetal death and low birth weight (11–17). An estimated one quarter of FGR cases show higher than normal expression levels of this gene in the placenta (11) making this a highly common alteration. *Phlda2* belongs to a remarkable family of genes which are subject to genomic imprinting, a process through which one parental allele is silenced by epigenetic events initiated in the parental germline (18). *Phlda2* is predominantly expressed from the maternal allele in the human and mouse placenta, with no evidence of expression in neuronal tissues (19–22)*. Phlda2* is located within a single imprinted domain called IC2 located on mouse chromosome 7/human chromosome 11p15 (19,20), and exclusively imprinted in Eutherians (23). *Phlda2 was originally identified as functioning to restrain placental growth based on the observation that mice lacking the maternal Phlda2 allele were found to have larger than normal placenta with a specific expansion of the junctional zone (referred to as spongiotrophoblast layer in this publication)* (24)*. A direct role for Phlda2 as a negative regulator of placental growth was then demonstrated in two ways. Firstly, a model of loss-of-imprinting of the IC2 domain, in which Phlda2 is elevated 2-fold, was combined with loss-of-expression of Phlda2. This genetic combination normalized Phlda2 expression levels and also restored placental weight and the junctional zone to near normal proportions in the model* (25)*. In this same study, mice carrying three copies of a bacterial artificial chromosome (BAC) transgene spanning Phlda2 were reported to have smaller placenta and to be >10% lighter at embryonic day 16.5 (E16.5)* (25)*. The junctional zone in rodents is composed of two distinct lineages called the spongiotrophoblast lineage and the glycogen cell lineages. Further studies on a single copy BAC transgenic line more precisely identified the function of Phlda2* as a negative regulator of just the spongiotrophoblast lineage with 2-fold *Phlda2* resulting in a 40% loss of this lineage in the mature mouse placenta and loss-of-function has the opposite effect, with an expanded spongiotrophoblast and no significant impact on the size of the glycogen cell lineage (26,27). Just 2-fold elevated *Phlda2* was also found to result in late onset, asymmetrical FGR with offspring born >10% lighter than their wildtype littermates, followed by rapid catch-up growth (28), suggesting that this alteration is a cause rather than a consequence of FGR in human pregnancy. The spongiotrophoblast is the major endocrine lineage in the rodent placenta synthesizing a number of hormones including *prolactin family 3, subfamily b, member 1* which encodes mouse placental lactogen (29–32). Placental lactogens function to modify the metabolic state of the mother securing nutrients to support fetal growth (31,33) consistent with the FGR phenotype in our model (28). Placental lactogens are also thought to prime the maternal brain in preparation for her caregiving of the newborn offspring (34–36). Consistent with this function, wildtype dams carrying offspring with different doses of *Phlda2* exhibit alterations in their maternal behaviour (37). Specifically, dams exposed to 2-fold *Phlda2* (low placental hormones) spend less time nurturing and grooming their pups, whereas those exposed to loss-of-function (high placental hormones) spend considerably more time nurturing their pups than control dams. The induction of maternal care is critically important for the successful rearing of healthy offspring and their behavioural outcomes (38) with maternal deprivation or naturally occurring low levels of licking and grooming shown to result in heightened stress reactivity, heightened fear response to novelty and poor learning/memory task performance in rodents (39–46). Consequently, *Phlda2* transgenic offspring expressing 2-fold *Phlda2* are growth restricted *in utero*, and both they and their non-transgenic littermates experience the same adverse maternal environment as a result of placental endocrine insufficiency.

**Figure 1:**
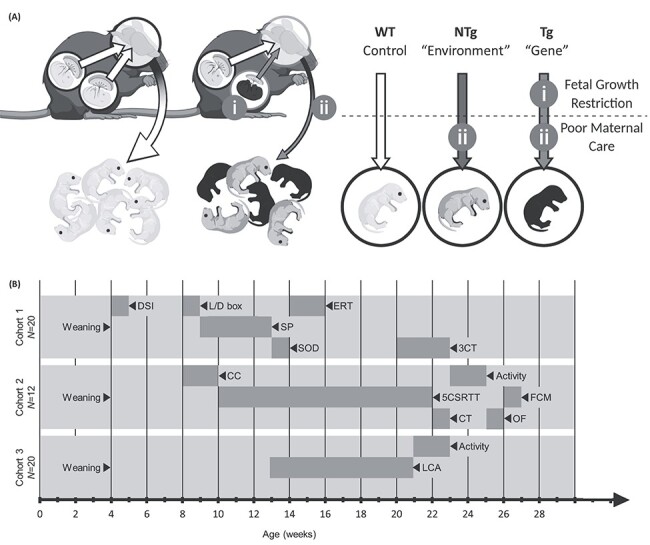
Experimental paradigm. **(A) Experimental groups. WT** = Wildtype offspring with normal expression (X1) of *Phlda2* born from a wildtype mating. **Tg** = Transgenic offspring with overexpression (X2) of *Phlda2* born from mating wildtype females to transgenic studs. These animals have a reduced placental endocrine compartment, exhibit restricted fetal growth (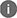) and are also exposed to an adverse maternal environment (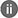). **NTg** = Non-transgenic offspring with normal expression (X1) of *Phlda2* born from mating wildtype females to transgenic studs, which are exposed to adverse maternal environment (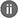).**(B) Timeline detailing the behavioural test batteries of the three cohorts. Cohort 1: DSI** = direct social interaction test, **L/D** = light/dark box test, **SP** = social propinquity test, **SOD** = social olfactory discrimination test, **ERT** = exploratory reluctance test, and **3CT** = three-chamber test. **Cohort 2: CC** = classical conditioning task, **5CSRTT** = five-choice serial reaction time task, **CT** = consumption test, **Activity** = locomotor activity, **OF** = open field test and **FCM** = fecal cortisol metabolite measure. **Cohort 3: LCA** = lick cluster analysis and **Activity** = locomotor activity.

Given the detrimental later life behavioural outcomes associated with being growth restricted *in utero* or being exposed to suboptimal maternal care early in life, we asked whether elevated *Phlda2* was associated with similarly detrimental outcomes. We generated offspring carrying the single copy BAC transgene spanning *Phlda2* (transgenic) and genetically wildtype littermates, which we labelled ‘non-transgenic’ as they are exposed to an adverse environment *in utero* and after birth. To distinguish the direct genetic effect of elevated *Phlda2* from the indirect environmental effect of the shared adverse environment induced by placental endocrine insufficiency, we included a fully wildtype control comparison (Fig. 1A). We undertook a series of behavioural tests and an RNA expression comparison of four adult brain regions previously implicated in early-life adversity (47). We observed behavioural and gene expression differences shared by the transgenic and non-transgenic offspring and absent in the fully wildtype animals, revealing the impact of sharing the adverse maternal environment induced by *Phlda2*-mediated placental endocrine insufficiency. We also discovered that the combined adversities of being growth restricted *in utero* and exposed to suboptimal maternal care after birth had the greatest consequences for later life behaviour, consistent with this sequential adversity.

## Results

Behavioural analysis was conducted on male and female transgenic (*Phlda2*^+/+BACx1(129)^; Tg) and non-transgenic littermates (*Phlda2*^+/+^; NTg) studied concurrently with fully wildtype (*Phlda2*^+/+^; WT) animals to control for the impact of the adverse maternal environment. Tests were selected to incorporate traits linked to early-life adversity in human pregnancy. Animals were tested in three separate cohorts of either N = 20 or 12, depending on the testing regime, and individual outcome measures were assigned to categories broadly encompassing the behavioural traits they probe (Supplementary Material, Table S1).

### Increased anxiety-like behaviours in both transgenic and non-transgenic offspring

**Figure 2:**
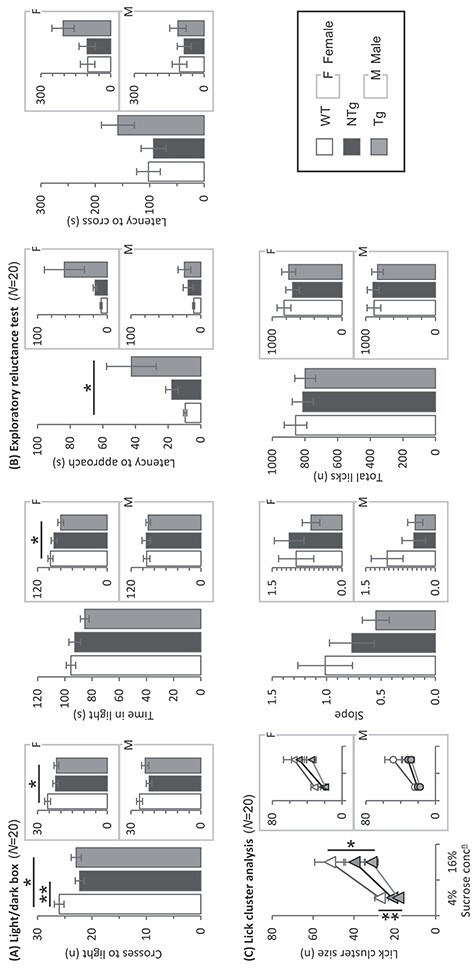
Probing anxiety and depression. **(A) Light/dark box test:**  *N* = WT(F), 20; NTg(F), 20; Tg(F), 20; WT(M), 20; NTg(M), 19; Tg(M), 20; Mean number of crosses into the anxiogenic light chamber and mean time spent within light chamber. Tg and NTg groups made fewer crosses into the light than WT (^*^P < 0.05 and ^*^^*^P < 0.01, respectively). Tg females made fewer crosses compared to WT (^*^P < 0.05). There were no differences between male groups. No group difference was seen in the time spent in the light. Tg females spent less time in the light than WT (^*^P < 0.05). There were no differences between male groups. **(B) Exploratory reluctance test:**  *N* = WT(F), 20; NTg(F), 20; Tg(F), 20; WT(M), 19; NTg(M), 20; Tg(M), 20. Mean latency to first approach to unknown home-cage and mean latency to first cross into unknown home-cage. Tg mice took longer to approach the unfamiliar home cage than WT (^*^P < 0.05). No differences were observed when split by sex. No differences were shown in the latency to cross into the unknown cage. **(C) Lick cluster analysis:**  *N* = WT(F), 19; NTg(F), 15; Tg(F), 18; WT(M), 20; NTg(M), 19; Tg(M), 20. Mean LCS with sucrose solutions of 4% (baseline) and 16%, slope describing the relative increase in LCS between baseline and 16% sucrose solution concentrations and mean total licks made in lickometry testing sessions. Tg mice made fewer licks at both 4 and 16% sucrose concentrations (^*^^*^P < 0.01 and ^*^P < 0.05, respectively). There were no differences when split by sex, nor in the relative slope of licking response. No difference in the total number of licks was observed. Error bars represent ±SEM.

Two different tests were used to probe anxiety-associated behaviours; the light/dark box and exploratory reluctance tests (Fig. 2). Tg animals made fewer crosses into the anxiogenic arena of the light/dark box compared to WT (GROUP: F_2,113_ = 5.19, *P* = 0.007; t_78_ = 3.01; Fig. 2A). Importantly, despite being genetically wildtype, the NTg animals also demonstrated a reduced number of crosses into the bright arena (*P* = 0.035 and t_77_ = 4.07, *P* = 0.009) and no significant difference was observed between Tg and NTg. When split by sex, a group difference was seen in females (GROUP: F_2,57_ = 3.35, *P* = 0.042). However, no significant differences were shown in the pairwise comparisons after correcting for multiple comparisons. No difference was detected between males. There were no significant differences between groups in the amount of time spent in the illuminated arena (GROUP: F_2,113_ = 2.06, *P* = 0.132)**.** However, when split by sex, female Tg mice spent significantly less time in the light than WT females (GROUP(F): F_2,57_ = 3.48, *P* = 0.0.38; t_38_ = 2.60, *P* = 0.031). There were no differences between the males. In the exploratory reluctance test, Tg but not NTg animals took significantly longer than the WT animals to approach the unfamiliar home-cage (GROUP: F_2,113_ = 3.59, *P* = 0.031; t_77_ = 2.57, *P* = 0.031 and t_77_ = 0.63, *P* = 0.802, respectively; Fig. 2B). No significant differences between the Tg and NTg or in sex-separated comparisons were found. There was no difference in the time to cross into the unfamiliar home-cage. Overall, the findings suggested heightened anxiety-like behaviours in both Tg and NTg offspring of both sexes.

### Depression-like behaviour in transgenic offspring

Depression-like behaviours were assessed by measuring hedonic response to sucrose reward using lickometry testing and included lick cluster size (LCS) and licking behaviour changes in response to increasing reward value (Fig. 2C). LCS generally increased between 4 and 16% sucrose concentrations as expected (CONC: F_1,102_ = 45.36, *P* < 0.001). LCS was reduced in Tg animals compared to WT at both 4 and 16% sucrose concentrations (GROUP: F_2,103_=5.02, *P* = 0.008; t_73_=3.52, *P* = 0.002 and t_73_=2.55, *P* = 0.036, respectively). No difference was detected for either 4% or 16% solutions between WT and NTg or between Tg and NTg groups. When split by sex, no group differences were observed. Response to increased sucrose concentration (slope of increased LCS between 4 and 16%) was not significantly different between groups or when split by sex. Motivation to work for the sucrose reward, as measured by total number of licks per session, was equivalent between groups, indicating that differences in LCS signify a deficit in hedonistic response (GROUP: F_2,103_ = 0.25, *P* = 0.782). These findings are indicative of depression-like behaviours in male Tg and NTg offspring.

### Learning deficits in transgenic and non-transgenic males

**Figure 3:**
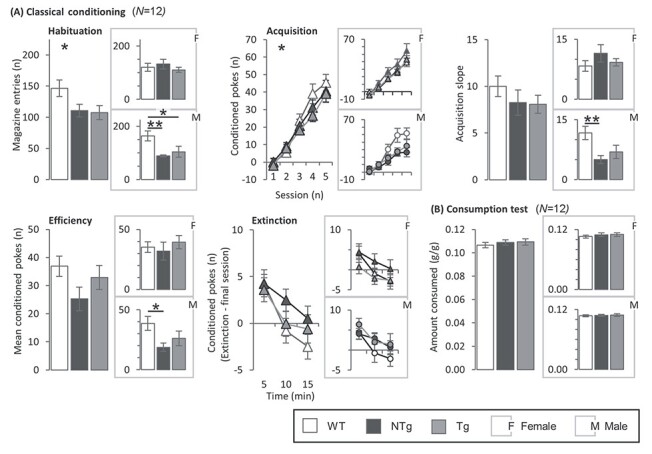
Learning in a classical conditioning task. **(A) Classical conditioning test:**  *N* = WT(F), 12; NTg(F), 11; Tg(F), 12; WT(M), 12; NTg(M), 12; Tg(M), 12. Total reward magazine entries made during the habituation phase, mean difference in the number of magazine entries during the conditioned stimulus (CS) and the inter-trial interval (ITI) periods per session in the learning phase, the slope describing the relative increase in CS magazine entries during the learning phase, mean difference in the number of magazine entries during the CS and ITI periods during sessions 6–10 and mean difference in CS responses made during an extinction test compared to the final test session. A main effect of group on habituation responses was observed (^*^P < 0.05). Tg (^*^P < 0.05) and NTg (^*^^*^P < 0.01) males made fewer entries to the reward magazine than WT, with no difference observed in females. A GROUP^*^SEX interaction was identified during the acquisition phase (^*^P < 0.05), with the learning slope of NTg males diminished compared to WT (^*^^*^P < 0.01), and they made fewer appropriate responses in the efficiency learning phase (^*^P < 0.05). No differences were observed during the extinction learning phase. **(B) Reward consumption test:** Weight of strawberry milk reward consumed per g of mouse in free access consumption test. There were no differences in amount consumed. Error bars represent ±SEM.

Aspects of learning were assessed in an operant-based classical conditioning test which included outcome measures of acquisition learning, conditioned responses and extinction learning (Fig. 3A). During the habituation phase of the classical conditioning training, WT animals made the greatest number of entries to the reward magazine and whilst there was a main effect of group, post hoc tests did not reach significance after correcting for multiple comparisons (GROUP: F_2,53_ = 3.76, *P* = 0.030; t_38_ = 2.37, *P* = 0.054 and t_37_ = 2.40, *P* = 0.051, respectively). When examined by sex, both Tg and NTg males made significantly fewer entries than WT males (GROUP(M): F_2,29_ = 6.25, *P* = 0.006; t_20_ = 2.53, *P* = 0.044 and t_20_ = 3.19, *P* = 0.009, respectively), whereas there was no difference between Tg and NTg males nor between female groups. The second and third figure panels show responses made to the conditioned stimulus (CS) per session during the learning phase and the data summarized as learning slopes. A GROUP^*^SEX interaction was observed in a repeated measures analysis of variance (ANOVA) (GROUP^*^SEX: F_2,65_ = 4.00, *P* = 0.023), but significance was not reached in post hoc analyses nor when split be sex. Whilst no main effect of group was seen in acquisition slope, when split by sex, the acquisition slope of NTg males was diminished compared to WT (GROUP(M): F_2,33_ = 5.30, *P* = 0.010; t_22_ = 3.16, *P* = 0.009). The difference between Tg and WT males was not significant after correcting for multiple comparisons (t_22_ = 2.26, *P* = 0.075) and no difference between male NTg and Tg was seen. Again, no group differences were found between the female groups. There was no difference in the number of responses made to the CS during the efficiency learning phase (fourth panel). When split by sex, NTg males were shown to make fewer CS responses than WT (GROUP(M): F_2,33_ = 4.12, *P* = 0.025; t_22_ = 2.83, *P* = 0.021), but there was no difference between WT and Tg or Tg and NTg males. No further differences were identified between the female groups. No differences in the extinction learning phase were seen between groups. A consumption test confirmed no differences in the motivation for the operant test reward with all groups consuming a similar amount of milkshake given free access (Fig. 3B), implicating an associative learning deficit rather than motivational deficits to explain the observed differences in the classical conditioning performance. Together, these tests supported cognitive deficits restricted to male Tg and NTg.

### Attention deficits in transgenic and non-transgenic males

**Figure 4:**
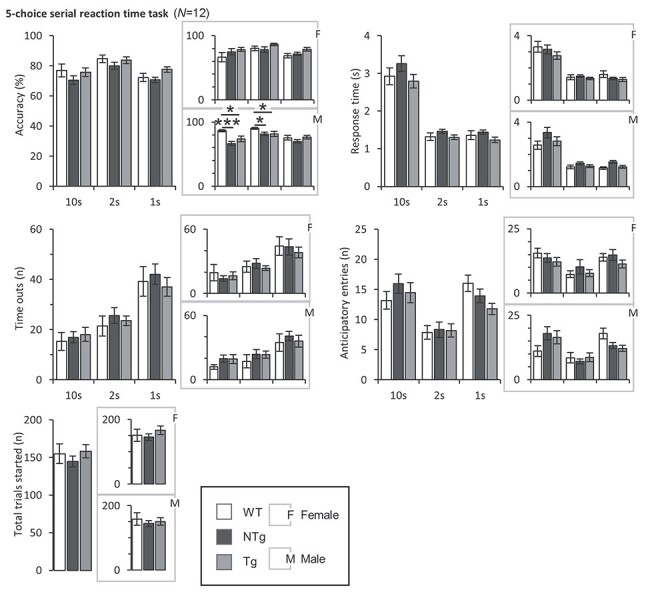
Measuring attention in the five-choice serial reaction time task (5CSRTT). **5CSRTT:**  *N* = WT(F), 8; NTg(F), 10; Tg(F), 10; WT(M), 9; NTg(M), 12; Tg(M), 12. Accuracy, correct response time, number of timeouts, number of anticipatory responses at stimulus lengths 10, 2 and 1 s and total trials started. No main effects of group on accuracy were found. When split by sex, Tg and NTg males were less accurate than WT at the 10 s (^*^P < 0.05 & ^*^^*^^*^P < 0.001, respectively) and 2 s stimulus lengths (^*^P < 0.05 & ^*^P < 0.05, respectively). No further differences in other measures were observed. Error bars represent ±SEM.

Attention was assessed using measures from the five-choice serial reaction-time task (5CSRTT). Measures taken from the 5CSRTT comprised of accuracy, reaction time and number of time-outs (Fig. 4). No main effects of group on accuracy were found. When split by sex, Tg and NTg males were less accurate than WT at the 10 s (^*^P < 0.05 & ^*^^*^^*^P < 0.001, respectively) and 2 s stimulus lengths (^*^P < 0.05 & ^*^P < 0.05, respectively). No other significant differences in accuracy were detected after correcting for multiple comparisons, nor between female groups. The reduction in accuracy suggests deficits in attention in the transgenic and non-transgenic males. However, there were no differences in other indices of attention such as reaction time and number of time outs incurred. The total number of trials started was comparable between groups, which together with similar total licks made in the LCA and free reward consumption strengthen the evidence for lack of motivational perturbation. Overall, deficits in attention were restricted to Tg and NTg males, and were relatively modest.

**Figure 5:**
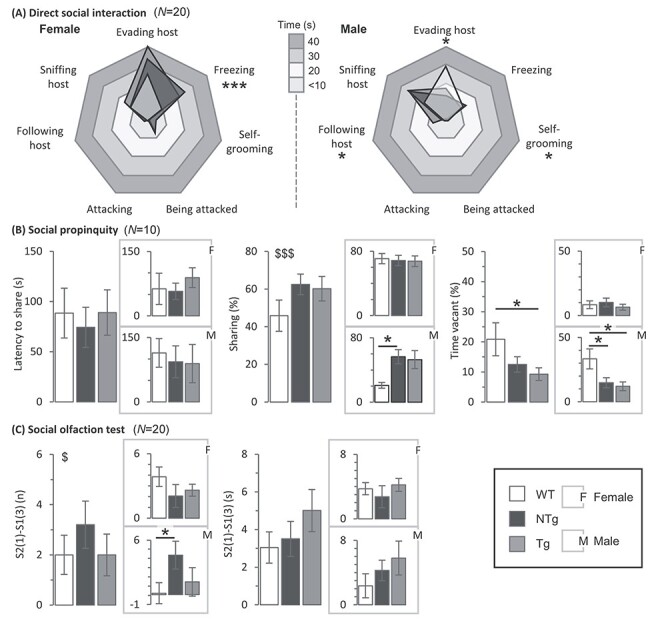
Probing social interactions. **(A)**, **Direct social interaction:**  *N* = WT(F), 20; NTg(F), 20; Tg(F), 20; WT(M), 20; NTg(M), 20; Tg(M), 20. Ethogram charts of direct social interaction task behaviours. Tg and NTg females exhibited freezing behaviour for a greater amount of time than WT (^*^^*^^*^P < 0.05 and *P* < 0.001, respectively). Tg males spent more time following the host than WT (^*^P < 0.05), and less time self-grooming (^*^P < 0.05). NTg males also spent more time following the host than WT (^*^P < 0.05) and less time avoiding the host (^*^P < 0.05). **(B) Social propinquity:**  *N* (pairs) = WT(F), 6; NTg(F), 9; Tg(F), 9; WT(M), 6; NTg(M), 9; Tg(M), 9. Mean latency to share the tube for the first time, proportion of time spent sharing and proportion of time tube was vacant. There was no difference in the latency to share the tube. Females spent longer sharing the tube than males (^$$$^*P* < 0.001). NTg males spent longer sharing than WT (^*^P < 0.05). Tg animals spent less time outside the tube than WT (^*^P < 0.05). Both Tg and NTg males spent significantly less time out in the open than WT (^*^P < 0.05). **(B) Social odour discrimination:**  *N* = WT(F), 20; NTg(F), 20; Tg(F), 20; WT(M), 20; NTg(M), 20; Tg(M), 20. Mean difference in the number of visits to and time spent investigating a familiar social odour (S1 (3)) and a novel social odour (S2 (1)). A GROUP^*^SEX interaction was identified (^$^*P* < 0.05). NTg males visited the novel social odour more times than the WT (^*^P < 0.05). Error bars represent ±SEM.

### Differences in baseline fecal cortisone metabolite concentration levels

Fecal cortisone metabolites (FCMs) were analysed as an indicator of stress levels and included baseline-state concentration, stressed-state concentration and change in concentration in response to stress (Supplementary Material, Fig. S1). At baseline Tg (GROUP: F_2,64_ = 4.77, *P* = 0.012; t_45_ = 2.40, *P* = 0.050) but not NTg mice had a lower concentration of FCMs than WT animals, but no difference between Tg and NTg was detected. When split by sex, female Tg had a lower baseline compared to WT (GROUP(F): F_2,31_ = 3.50, *P* = 0.043; t_21_ = 2.48, *P* = 0.048), with no differences detected between NTg and WT or Tg groups, or between any male group. Under stressed conditions, no differences between groups were found, nor was there a difference in the percentage change in FCM concentrations in response to stress.

### Evidence for atypical social behaviour in transgenic and non-transgenic males

Sociability traits were assessed through the direct social interaction (Fig. 5A), social propinquity (Fig. 5B), social olfaction (Fig. 5C) and three-chamber tests (Supplementary Material, Fig. S2). During the direct social interaction test, compared to WT, both Tg and NTg females spent more time exhibiting socially anxious behaviours such as freezing (GROUP: F_2,57_ = 7.94, *P* < 0.001; t_38_ = 2.83, *P* = 0.017 and t_38_ = 3.84, *P* = 0.001, respectively) but no differences in moving away from the host whilst being followed (Fig. 5A). In contrast, Tg and NTg males spent more time being socially interactive compared to WT, including following the host more (GROUP: F_2,57_ = 4.18, *P* = 0.020; t_38_ = 2.52, *P* = 0.038 and t_38_ = 2.48, *P* = 0.042, respectively) and evading the host less (NTg only; GROUP: F_2,57_ = 3.87, *P* = 0.027; t_38_ = 2.73, *P* = 0.023). They also spent more time exhibiting anxiety-linked self-grooming behaviour (Tg only; GROUP: F_2,57_ = 3.58, *P* = 0.034; t_38_ = 2.59, *P* = 0.032). Whilst a group difference was detected for sniffing the host, *post hoc* analysis comparing WT and Tg groups did not yield a difference once corrected for multiple comparisons (GROUP: F_2,57_ = 3.36, *P* = 0.042, t_38_ = 2.35, *P* = 0.056). There were no group differences in the latency to share the tube in the social propinquity test (Fig. 5B), or when split by sex. However, females spent longer sharing the tube with the unfamiliar mouse than males (SEX: F_1,42_ =18.53, *P* < 0.001), and a significant interaction was identified (GROUP^*^SEX: F_2,42_ = 4.21, *P* = 0.022). NTg males spent more time than WT sharing the tube with the unfamiliar mouse (GROUP(M): F_2,21_=5.40, *P* = 0.013; t_13_ = 2.54, *P* = 0.048). There was no difference between Tg and either WT or NTg males or any female group. In the anxiety-related aspect of the social propinquity test, Tg animals spent less time outside the tube in the illuminated arena than WT (GROUP: F_2,42_ = 5.75, *P* = 0.006; t_38_ = 2.91, *P* = 0.014), and no difference was detected between NTg and either WT or Tg groups. There was a main effect of SEX (SEX: F_1,42_ = 16.65, *P* < 0.001) and a GROUP^*^SEX interaction (GROUP^*^SEX: F_2,42_ = 5.34, *P* = 0.009) driven by WT males spending less time in the tube. Splitting by sex revealed that both Tg and NTg males spent significantly less time in the open arena than the WT males (GROUP(M): F_2,21_ = 7.53, *P* = 0.003; t_15_ = 3.03, *P* = 0.017 and t_15_ = 2.63, *P* = 0.039, respectively). There was no difference between the female groups. In the social odour discrimination test, a GROUP^*^SEX interaction was identified (GROUP^*^SEX: F_2,114_ = 3.23, *P* = 0.043), with WT females visiting the novel social smell more than WT males (t_38_ = 2.21, *P* = 0.029), and no sex differences between NTg or Tg groups. NTg males visited the novel odour more times than the WT males (t_38_ = 2.50, *P* = 0.041; Fig. 5C). No further differences were observed. There were no group differences in the time spent at the novel social smell, or when split by sex. There were no differences in behaviours within the three-chamber test on individual outcome measures including number of crosses into the empty or occupied chambers, the time spent in the empty and occupied chambers, the latency to enter into or the mean duration of visits to the occupied chamber (Supplementary Material, Fig. S2). Compared to males, females spent a greater amount of time in the empty chamber (SEX: F_1,112_ = 7.88, *P* = 0.006) and less in the occupied chamber (SEX: F_1,112_ = 6.57, *P* = 0.012). Females also visited the occupied chamber more than males (SEX: F_1,112_ = 3.92, *P* = 0.050) and took longer to enter the occupied chamber (SEX: F_1,112_ = 9.31, *P* = 0.003). This behavioural assessment suggested overall higher levels of sociability in males, both Tg and NTg.

Activity within an open field arena and measured over a 24 h period were used to compare motor performance and activity levels (Supplementary Material, Fig. S3A and B, respectively). No differences were observed in total distance moved, movement time or velocity within the open field, nor in the number of beam breaks within the dark or light period during of the locomotor activity test. These results support the assumption that deficits seen in operant-based tests are not due to impaired movement, hyper- or hypoactivity in the Tg and NTg animals.

### Behavioural results summary

**Figure 6:**
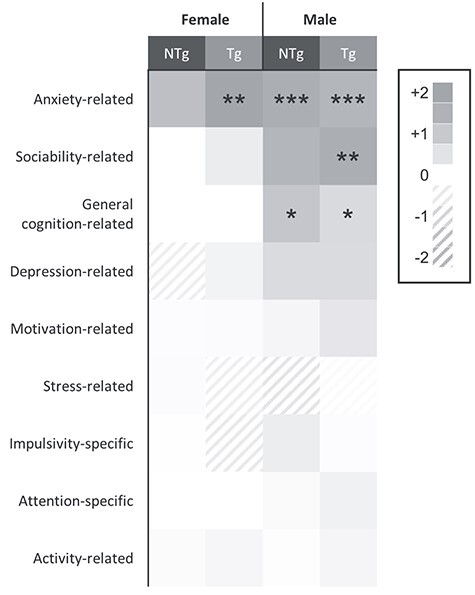
Summary heatmap of behavioural changes represented by fold change in prevalence of behavioural trait deficits in NTg and Tg offspring compared to WT control group populations. Raw data of individuals were normalized within sex groups to provide a score between 0 and 1 for each outcome measure, with ‘0’ representing the lowest value and ‘1’ representing the greatest value. Scores from outcome measures probing related behavioural traits were combined to provide a behavioural trait score. Anxiety-related scores included crosses to and time spent in the light in the light/dark box test, percentage time tube was vacant in the social propinquity test, latency to approach and cross in the exploratory reluctance test. Sociability-related scores included all direct social interaction measures, latency to share and time spent sharing the tube in the social propinquity test, visits to and time spent investigating social odours in the odour discrimination test and all three-chamber test measures. Cognition-related scores included number of responses in all phases of the classical conditioning task and acquisition slope. Depression-related scores included LCS and reward response from LCA. Motivation-related scores included number of habituation responses in classical conditioning, total trials started in five-choice serial reaction time task, total number of licks in the LCA and amount consumed in reward consumption test. Stress-related scores included baseline, stressed and change in response in the fecal corticosterone metabolite analysis. Impulsivity-related scores included number of anticipatory responses in the five-choice task. Attention-related scores included accuracy, response time and number of time outs in the five-choice task. Activity-related scores included distance moved, time spent moving and velocity in the open field test and number of beam breaks in the locomotor activity test. ‘0’   represents the proportion of animals within the WT groups scoring greater than the cohort median for each behavioural trait (prevalence baseline). Positive  and  negative values represent an increase or decrease, respectively, in the proportion of animals within a group scoring a greater behavioural deficit than the cohort median compared to the WT baseline. ^*^P < 0.05, ^*^^*^P < 0.01 and ^*^^*^^*^P < 0.001 indicate where a significant difference from WT scores was observed. The female Tg population was affected to a greater extent than the NTg females, with prevalence of anxiety-like outcomes increasing to the greatest degree. Prevalence of behavioural deficits in the male mice was typically 2- to 3-fold greater than in the female population, with both Tg and NTg mice affected. The prevalence of anxiety-like behaviours, sociability-related and cognition disturbances in the male population were increased to the greatest extent.

Subtle changes in behaviour were apparent both in the genetically altered offspring and their littermates sharing the adverse environment in a number of behavioural tests. To gain further insight into these changes and overcome the potential for issues arising through multiple testing, scores from outcome measures probing related behavioural traits were combined to provide a behavioural trait score (48). Raw data of individuals were normalized within sex groups to provide a score between 0 and 1 for each outcome measure, with ‘0’ representing the lowest value and ‘1’ representing the greatest value. Prevalence of behavioural deficit scores within the Tg and NTg groups compared to WT are summarized in Figure 6. Significantly increased anxiety-related behavioural scores were apparent in both female and male Tg offspring compared to WT (GROUP(M): F_2,57_ = 11.72, *P* < 0.001; t_38_ = 4.45, *P* < 0.001 and GROUP(F): F_2,57_ = 6.11, *P* = 0.004; t_38_ = 3.42, *P* = 0.003, respectively). Whilst behavioural deficits in Tg females were restricted to anxiety-related traits, Tg males also showed evidence of atypical social behaviours and cognitive deficits (GROUP(M): F_2,57_ = 5.16, *P* = 0.009; t_38_ = 3.17, *P* = 0.007 and GROUP(M): F_2,33_ = 4.853, *P* = 0.014; t_22_ = 2.45, *P* = 0.050, respectively).

**Figure 7:**
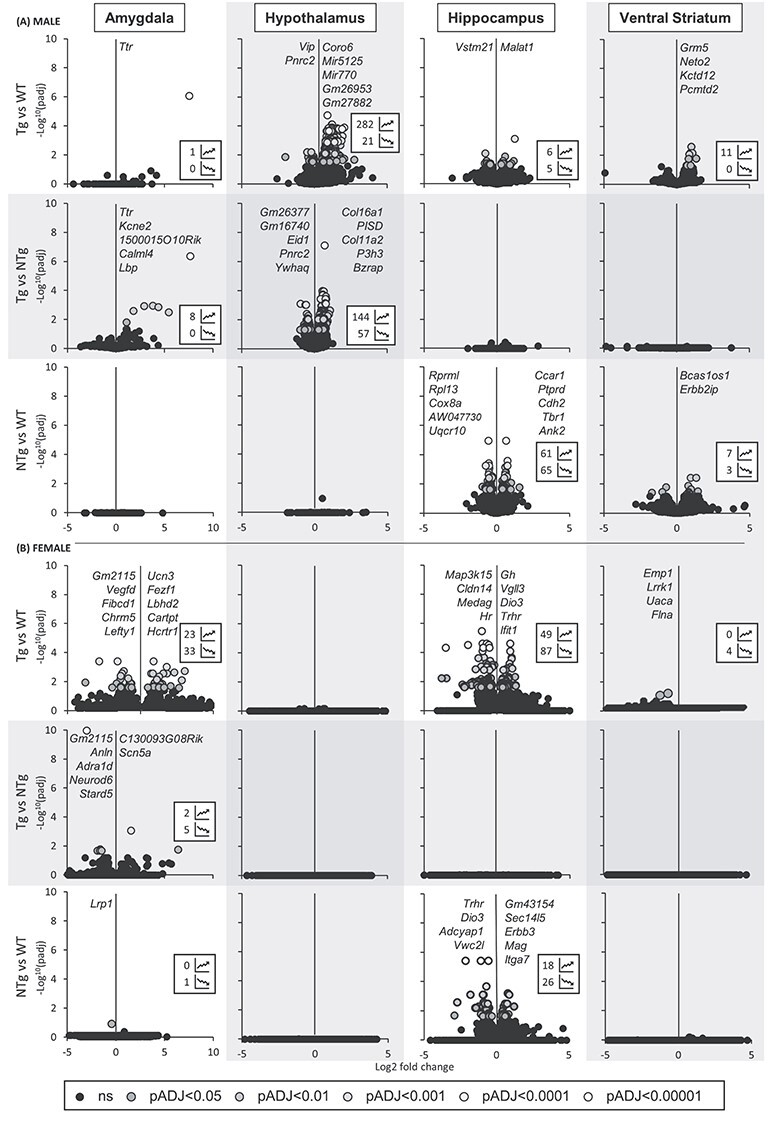
Analysis of RNAseq data reveals gene and environmental effects. **Volcano plots of gene expression changes in pairwise comparisons normalized to brain region in P21 males (A) and females (B). [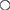 = adj.pval < 0.00001,  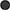 = adj.pval < 0.0001,  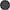 = adj.pval < 0.001,  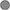 = adj.pval < 0.01,  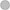 = adj.pval < 0.05 and   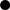 = ns].** Inset values show the total number of adj.pval significant upregulated and downregulated gene expression changes, and listed genes represent the top five genes with the greatest fold change expression adj.pval < 0.01. **First and fourth rows:** Tg compared to WT groups. **Second and fifth rows:** Tg compared to NTg. **Third and sixth rows:** NTg compared to WT.

The NTg populations, despite sharing a wildtype genotype with the WT, also demonstrated an increase in anxiety-related behaviours. This was particularly evident in the male NTg population GROUP(M): F_2,57_ = 11.72, *P* < 0.001, t_38_ = 3.87, *P* = 0.001), and whilst females showed a general increase in anxiety-related behaviours, differences in anxiety score did not reach significance once corrected for multiple comparisons. The female NTg population did not show any further deviation from WT, demonstrating similar incidences of non-typical sociability, cognition, depression-related behaviours, motivation, stress, impulsivity, attention and activity. In contrast, the male NTgs showed a similar increase in incidence of cognitive deficits to Tg when compared to WT (GROUP(M): F_2,33_ = 4.853, *P* = 0.014; t_22_ = 2.89, *P* = 0.018) and a general increase in the prevalence of sociability-related deficits. However, differences in sociability scores did not persist after correcting for multiple comparisons (t_38_ = 2.05, *P* = 0.109). There was a general increase in the prevalence of depression-related behaviours in males of both Tg and NTg littermates compared to WT, but no significant difference in mean depression-related behavioural scores. Prevalence of negative behavioural scores relating to motivation, stress, impulsivity, attention and activity were relatively similar to WT. Overall, there was a higher prevalence of adverse behavioural scores in the males compared to the females indicated by a greater number and intensity of red squares in the matrix, and a greater impact on Tg compared to NTg offspring.

### Transcriptional alterations to key regions of the adult brain in both transgenic and non-transgenic offspring

To identify alterations in the brains that might underlie the changes in behaviour, we performed whole-genome RNA sequencing (RNAseq) on postnatal day (P) 21 male and female hypothalamus (Hyp), ventral striatum (VS), hippocampus (Hip) and amygdala (Amyg) from transgenic (*Phlda2*^+/+BACx1(129)^; Tg) and non-transgenic (*Phlda2*^+/+^; NTg) experimental animals and fully wildtype (*Phlda2*^+/+^; WT) controls.

Gene expression values normalized by sex-matched brain region were compared between groups in both male and female tissues to identify differentially expressed genes (DEGs; adj.pval < 0.05; Fig. 7A-B). The Tg, which are both FGR and exposed to an adverse maternal environment, showed the greatest number of DEGs in comparison to WT for both male and female animals (First rows of Fig. 7A and B) with a total of 326 identified in males, and 196 in females. For males, the majority of changes were observed within the Hyp (n = 303), Hip (n = 11), and VS (n = 11) compared to the Amyg (n = 1). In females, DEGs were restricted to the Hip (n = 135) and Amyg (n = 48) only. The most highly significant expression changes involved genes associated with neurodevelopment (M: *Ttr*, *Coro6*, *Pcmtd2*, *Gm26953*, *Gm27882*; F: *Fezf1, Chrm5*), neuroprotection (M: *Malat1*, *Vastm21*; F: *Hcrtr1*), modulation of the limbic system (M: *Vip*, *Neto2*, *Kctd12*, F: *Ucn3, Cartpt, Trhr*) and regulation of metabolism (M: *Pnrc2*; F: *Map3k15*) (Supplementary Material, Table S2A). For Tg and NTg comparisons (Second rows of Fig. 7A and B), DEGs were identified in both male and female Amyg (n = 8 and n = 7, respectively). A much greater number of DEGs were identified in male Hyp (n = 201). These genes related to neurodevelopment (M: *1500015O10Rik*, *Calml4*, *Lbp*, *YWHAQ*; F: *Neurod6*), endocrine modulation (M: *Kcne2*, *Calml4*, *Eid1*, *Ttr*; F: *Adea1d, Scn5a*) and neurotransmitter release (M: *Bzrap1*, *Kcne2*; F: *Neurod6*) (Supplementary Material, Table S2B). Despite being genetically identical, DEGs were detected between NTg and WT in the Hip of both male (n = 126) and female (n = 49), as well as the male VS (n = 10), indicative of an environmental impact, with none in the Amyg or Hyp of either sex (Third rows of Fig. 7A and B, Supplementary Material, Table S2C). Significant DEGs in these comparisons broadly related to neurodevelopment (M: *Tbr1, Ank2, Ptprd*; F: *Erbb3, Dio3, Vwc2l*), signalling (M: *Erbb2ip*; F: *Erbb3, Mag)* neuroendocrine system (F: *Erbb3, Dio3, Adcyap1*) cellular metabolism (M: *Cox8a, Uqcr10*).

Importantly, a number of common DEGs were identified between gene lists of the pairwise comparisons. These ‘shared’ gene changes support evidence for both genetic and maternal environment effects on the observed gene expression changes, dependent on the experimental groups within which the DEGs are shared. DEGs shared between ‘Tg vs WT’ and ‘Tg vs NTg’ pairwise comparisons imply a ‘genetic’ cause since these are Tg specific; Supplementary Material, Table S3A and B, and DEGs shared between ‘Tg vs WT’ and ‘NTg vs WT’ comparisons indicate an ‘environmental’ cause since the NTg and WT animals are genetically identical with the only difference bring their environment; Supplementary Material, Table S3C and D. Pathway analysis of these shared genes revealed significantly affected gene networks and their associated biological and molecular processes (Table 1). *Transthyretin (Ttr;* FC x186 and adj.pval = 0.0000008) was the only ‘gene’ effect DEG identified in the Amyg of male animals, and just six mostly downregulated genes in the females including *Neuronal Differentiation 6* (*Neurod6*; FC x-.3 and adj.pval = 0.008) (Table 1A). The male Hyp yielded many ‘gene’ effect DEGs (n = 112; Fig. 8B), the majority of which were upregulated (91%). The most significant associated pathways involved regulation of synaptic proteins (*TSPO Associated Protein 1* (*Bzrap1*; FC x1.9, adj.pval = 0.000003), neuronal development (*SET Domain Containing 1A, Histone Lysine Methyltransferase* (*Setd1a*; FC x1.4, adj.pval = 0.002), *Histone Deacetylase 7* (*Hdac7*; FC x1.8, adj.pval = 0.02) and neural crest differentiation (*Dishevelled Segment Polarity Protein 1* (*Dvl1*; FC x1.5, adj.pval = 0.02)). There were no ‘gene’ effect DEGs in the female Hypo, nor in either the male or female Hip or VS.

**Table 1:** RNAseq pathway analysis. **(A)** Differentially expressed genes (DEGs, adj.pval < 0.05) shared between Tg vs WT and NTg vs WT comparisons (‘environmental’ effects and **(B)** between Tg vs NTg and Tg vs WT comparisons (‘gene’ effects**)**. **Amyg** = amygdala, **Hypo** = hypothalamus, **Hip** = hippocampus and **VS** = ventral striatum.   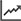 represents number of upregulated genes,   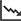 represents number of downregulated genes. Top pathways were calculated from gene lists inputted into Enrich-R pathway analysis software and described by ‘KEGG 2019 Mouse’, ‘GO Biological Process 2018’ and ‘GO Molecular Function 2018’ databases. **pval** = unadjusted *P*-value, **adj.pval** = adjusted *P*-value, **O.R.** = odds ratio, **C.S.** = combined score (log(pval) multiplied by z-scoren/a denotes where too few DEGs were shared to perform analyses.

(A) Shared DEGs between Tg vs WT and Tg vs NTg (Genetic effect)									
	Sex	N^o^ shared DEGs			Top pathways	pval	adj. pval	O.R.	C.S
		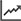	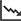						
Amyg	M	1	0		n/a	n/a	n/a	n/a	n/a
	F	1	5	Enriched pathways	Monoamine GPCRs Calcium regulation in the cardiac cell	0.010 0.043	0.040 0.077	124.8 27.2	576.3 85.4
				Biological processes	Cholesterol import Sterol import Regulation of synaptic glutamatergic transmission Regulation of cytosolic calcium ion concentration Dopamine receptor signalling pathway	0.002 0.002 0.003 0.003 0.003	0.017 0.017 0.017 0.017 0.017	799.6 799.6 499.7 444.1 444.1	5053.7 5053.7 2955.7 2580.4 2580.4
				Molecular function	Alpha-adrenergic receptor activity Dopamine neurotransmitter receptor activity Dopamine binding Ammonium ion binding Dopamine neurotransmitter receptor activity	0.002 0.003 0.003 0.004 0.004	0.010 0.010 0.010 0.010 0.010	799.6 499.7 399.7 333.0 333.0	5053.7 2955.7 2284.2 1847.8 1847.8
Hypo	M	102	10	Enriched pathways	NOVA regulated synaptic proteins Focal adhesion Neural crest differentiation	0.023 0.004 0.019	0.285 0.143 0.285	9.02 5.12 5.56	34.0 28.4 22.0
				Biological processes	Decapping of mRNA Receptor clustering Epithelial tube morphogenesis Regulation of axon extension Regulation of axonogenesis	4.59E-04 9.08E-06 3.80E-05 2.79E-04 2.55E-04	0.068 0.007 0.015 0.054 0.054	90.4 36.8 24.5 27.3 14.4	694.6 427.1 249.6 223.8 119.2
				Molecular function	Histone methyltransferase activity Histone-lysine N-methyltransferase activity Regulation of postsynaptic membrane potential Protein-lysine N-methyltransferase activity Histone methyltransferase activity	0.004 0.002 0.007 0.002 0.002	0.140 0.140 0.206 0.140 0.140	25.8 14.4 17.2 12.4 12.1	145.6 92.9 84.7 75.3 72.9
	F	0	0		n/a	n/a	n/a	n/a	n/a
Hip	M	0	0		n/a	n/a	n/a	n/a	n/a
	F	0	0		n/a	n/a	n/a	n/a	n/a
VS	M	0	0		n/a	n/a	n/a	n/a	n/a
	F	0	0		n/a	n/a	n/a	n/a	n/a
(B) Shared DEGs between Tg vs WT and NTg vs WT (environmental effect)									
							
	Sex	N ^o^ shared DEGs			Top pathways	pval	adj. pval	O.R.	C.S
		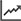	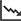						
Amyg	M	0	0		n/a	n/a	n/a	n/a	n/a
	F	0	0		n/a	n/a	n/a	n/a	n/a
Hypo	M	0	0		n/a	n/a	n/a	n/a	n/a
	F	0	0		n/a	n/a	n/a	n/a	n/a
Hip	M	6	13	Enriched pathways	Oxidative phosphorylation Parkinson disease Non-alcoholic fatty liver disease Alzheimer disease Huntington disease	6.91E-06 9.18E-06 1.11E-05 1.98E-05 2.85E-05	4.80E-05 4.80E-05 4.80E-05 6.44E-05 7.41E-06	40.7 37.8 36.0 30.9 28.1	483.9 438.3 410.6 334.6 293.8
				Biological processes	Mitochondrial ATP synth^s^ coupled electron transport Aerobic respiration Respiratory electron transport chain Mitochondrial electron transport Mitochondrial respiratory chain complex assembly	1.12E-06 3.19E-04 1.68E-06 8.63E-04 1.01E-04	7.39E-05 0.007 7.39E-05 0.015 0.003	65.5 90.3 58.9 53.3 39.7	897.6 727.1 783.7 376.1 364.9
				Molecular function	Phosphatidylethanolamine binding Oxidoreductase activity Ubiquinol-cytochrome-c reductase activity NADH dehydrogenase activity Oxidoreductase activity	0.006 0.008 0.008 0.010 0.014	0.056 0.056 0.056 0.057 0.062	222.0 158.5 158.5 111.0 79.2	1147.4 774.0 774.0 506.6 337.3
	F	17	18	Enriched pathways	Dopaminergic Neurogenesis Mecp2 and Associated Rett Syndrome SIDS Susceptibility Pathways Peptide GPCRs Spinal Cord Injury	0.001 0.003 0.006 0.008 0.014	0.028 0.034 0.038 0.038 0.058	40.7 25.3 19.3 16.5 11.7	267.3 143.6 99.7 80.3 49.6
				Biological processes	Neuron migration Positive regulation of kinase activity Regulation of neuron projection development Regulation of signal transduction Positive regulation of ERK1 and ERK2 cascade	0.002 0.001 0.001 0.001 0.006	0.139 0.122 0.122 0.122 0.139	30.0 15.9 15.1 10.5 8.8	179.7 107.3 99.5 73.5 45.0
				Molecular function	Acetylglucosaminyltransferase activity Receptor protein tyrosine kinase activity Chloride channel activity Transmembrane receptor protein kinase activity Mitogen-activated protein kinase binding	0.004 0.006 0.006 0.006 0.006	0.090 0.090 0.090 0.090 0.090	23.7 19.3 19.3 19.0 18.1	131.8 99.7 99.7 97.4 91.1
VS	M	2	0	n/a	n/a	n/a	n/a	n/a	n/a
	F	0	0	n/a	n/a	n/a	n/a	n/a	n/a

DEGs attributed to an ‘environment’ effect were predominantly observed in the Hip, both in male (n = 19, mostly downregulated) and female (n = 35, both upregulated and downregulated) offspring (Table 1B). Disturbed pathways associated with these genes relate to neuronal development (M: *T-Box Brain Transcription Factor 1* (*Tbr1*; FC x1.5, adj.pval = 0.02), *Pleckstrin And Sec7 Domain Containing* (*Psd*; FC x-1.4, adj.pval = 0.02), *V-Set And Transmembrane Domain Containing 2 Like* (*Vstm2l*; FC x-1.6, adj.pval = 0.02)), synapse (*NADH:Ubiquinone Oxidoreductase Core Subunit S7* (*Ndufs7*; FC x-1.4, adj.pval = 0.04); F: *Ephrin B2* (*Efnb2*, FC x-0.6, adj.pval = 0.007), *Ret Proto-Oncogene* (*Ret*; FC x-0.6, adj.pval = 0.005), *Myelin Associated Glycoprotein* (*Mag*; FC x1.8, adj.pval = 0.004)) and mitochondrial function (M: *Cytochrome C Oxidase Subunit 8A* (*Cox8a*; FC x-1.4, adj.pval = 0.02), *Ubiquinol-Cytochrome C Reductase, Complex III Subunit X* (*Uqcr10*; FC x-1.5, adj.pval = 0.02)). Just two significant male VS DEGs could be attributed to an ‘environment’ effect: *Erb-B2 Receptor Tyrosine Kinase 2 Interacting Protein* (*Erbb2ip*; FC x1.9 and adj.pval = 0.004) encoding a protein associated with early-life stress, anxiety disorders and ADHD (49) and *SECIS Binding Protein 2 Like* (*Secisbp2l*; FC x1.7 and adj.pval = 0.013), linked to thyroid hormone-dependent growth and embryonic differentiation processes (50).

In summary, gene alterations were present in brains of both Tg (‘gene’ effect; experiencing FGR and exposure to abnormal maternal environment) and NTg (experiencing exposure to abnormal maternal environment only) offspring in comparison to WT controls, with all four sampled regions impacted in Tg males (Fig. 7A), and hypothalamus spared in females (Fig. 7B). In contrast, for NTg animals, the hippocampus was solely (females) or predominantly (males) affected, with the males also demonstrating some expression changes in ventral striatum.

## Discussion

Our work here demonstrates that transgenically elevated expression of *Phlda2* drives alterations in the brains and behaviour of mouse offspring, both for those carrying the transgene that induces changes to the placenta and for those individuals sharing the abnormal early-life environment driven by placental endocrine insufficiency. This is the first example, to our knowledge, of a common genetic placental alteration reported in human FGR that results in alterations in offspring behaviour later in life. Heightened anxiety and deficits in cognition have both been reported in children growth restricted *in utero* and those exposed to poor maternal care. Our experimental strategy enabled us to distinguish between the direct genetic effect of elevated *Phlda2* and the impact of an abnormal environment driven by placental endocrine insufficiency which includes suboptimal maternal caregiving (37). Placental endocrine insufficiency is a highly common outcome of a number of prenatal adversities experienced by the mother which are also associated with a variety of undesirable outcomes for her offspring. Our findings are therefore relevant not only to *Phlda2* but to any genetic or environmental insult that results in placental endocrine insufficiency, of which there are many.

First and foremost, our findings are important because the alteration we are modelling in mice, which is elevated placental *Phlda2*, has been reported in a substantial number of studies on human FGR (51). FGR is estimated to occur in 3–7% of pregnancies and is linked to poor outcomes in the short and longer term (2,52–54). An estimated 25% of infants that are growth restricted *in utero* have abnormally high levels of *PHLDA2* in their placenta (11). Our previous work experimentally established causality by demonstrating that mice modelling this alteration are growth restricted, being born approximately 10% lighter than normal with relative sparing of the head (28). We further identified substantial defects in the placental endocrine compartment in this model (26,27) and deficits in the maternal care provided by WT dams carrying and caring for these mutant offspring (37). The current study provides the first indication that the specific alteration of elevated *PHLDA2* also programs alterations in later life behaviour of offspring. Given the prevalence of elevated *PHLDA2* in FGR infants, this alteration may contribute to the behavioural disorders observed in this high-risk population.

The second important aspect to this work is that we can attribute changes in offspring behaviour to the abnormal maternal environment rather than the direct effect of the genetic alteration. We are able to make this conclusion because both the transgenic animals and their non-transgenic littermates exhibit similar alterations in behaviour, albeit with a greater impact on transgenic offspring. A previous study has demonstrated that placental insufficiency driven by the altered expression of an imprinted gene is sufficient to modify offspring behaviour, with mice null for the placenta-specific P0 transcript of *insulin-like growth factor-2* exhibiting increased reactivity to anxiety-provoking stimuli (55). However, in this model the atypical behaviour was apparent in the genetically modified offspring in comparison to their littermates. In contrast, our study demonstrates atypical behaviour in both the genetically modified offspring and their non-transgenic littermates sharing the early-life environment. While it is possible that the transgenic offspring are directly influencing their non-transgenic siblings, the most straightforward explanation for our findings is that the shared behavioural and transcriptional alterations are due to the wider adversity caused by defects in the placental endocrine lineage, which include atypical maternal care (37). Demonstrating the importance of placental endocrine lineage development *per se* rather than one specific placental hormone is critical for the wider interpretation of this work because placental hormones are considerably diverse between species of mammal, as are placental structures (56). However, the fundamental function of placental hormones in inducing maternal adaptions in pregnancy is well conserved (31). Moreover, we have data from human pregnancies demonstrating the same inverse correlation between placental *PHLDA2* and serum human placental lactogen (16). Therefore, our findings in mice are likely to be relevant to FGR in human pregnancy.

A third aspect to this work is the wider relevance of placental endocrine insufficiency and the programming of neurological disorders in children. Maternal undernutrition in both mice and rats results in a loss of spongiotrophoblast lineage alongside the reduced expression of a number of placental hormones expressed from this lineage (57–63). Most recently, a low-protein diet from two weeks pre-pregnancy through gestation has been reported to result in a reduction of the spongiotrophoblast and glycogen cell lineages alongside elevated *Phlda2* (64). Disruption in the expression of placental hormones has also been reported in response to gestational exposure to bisphenol A (65), chromium (VI) (66), dexamethasone (67) and perfluorooctanoic acid (68), and in response to the physical stressor of reduced uteroplacental perfusion pressure (69). Given the importance of placental hormones for fetal growth, maternal nurturing and the offspring’s behavioural outcomes, placental endocrine insufficiency may mediate the well-established relationship between prenatal adversities experienced by the mother and these undesirable outcomes for both her and her offspring. Evidence that these observations in experimental models are relevant to human pregnancy may come from studies of maternal obesity (70,71) and depression (72,73) where lower level of placental lactogen has been reported. However, further work is required to link specific placental hormone deficiencies to neurological disorders in human populations.

In our study we applied a unified behavioural scoring system combining results across multiple test measures interrogating similar behaviours. In addition to overcoming potential issues with multiple testing, this system consolidates information obtained across multiple test measures which individually might not reach significance but, when combined, uncover the most subtle phenotypes (48). This approach is particularly important when assessing the impact of an environment rather than genetic alteration, as is the case for the current study. The specific behaviours uncovered by this approach were heightened anxiety, atypical social behaviour and deficits in cognition. Most prominent were the anxiety-related behaviours evident in both Tg and NTg animals in individual measures across a number of tests, further highlighted when all anxiety-related measures were combined in the unified score. Anxiety is often comorbid with depression, the latter being a major feature of many neurological disorders linked to early-life adversity (74). While we observed a general decrease in hedonistic response, which is associated with depression-related behaviours, in the Tg group and NTg males demonstrating a depression scores closer to Tg than WT, evidence for depressive symptoms was modest. Both Tg and NTg males had altered social behaviours, showing an increased interest and willingness to interact and co-habit with an unknown individual. In human infants surgency is a temperamental characteristic associated with active, sociable and approach behaviours. While these behaviours can be positive, they have also been identified as indicators of behavioural dysregulation associated with psychopathology (e.g. ADHD) throughout childhood and into adulthood (75). Low accuracy scores observed of Tg and NTg male offspring in the 5CSRTT might be indicative of reduced attentional control. The fact that no deficits in response time or number of missed trials were found could also indicate a more general reduction in rule-learning cognition, an argument strengthened by the learning deficits observed in the classical conditioning task (CCT).

Alongside the changes in behaviour, we observed alterations to the male and female hippocampal transcriptomes shared by transgenic and non-transgenic animals. Hippocampal changes in both males and females were associated with downregulation of genes linked to neuronal differentiation. The anxiety-like phenotype was consistent with these shared transcriptional changes in the hippocampus, a brain region linked to anxiety-driven behaviours (76). Social behaviours are also dependent, at least in part, on the Hip and this region has been linked to maladaptive social behaviour in psychiatric disorders (77). Furthermore, disturbances within the nucleus accumbens (NAc), the main component of VS, have been associated with social behavioural disorders (78) as seen in the males. Several DEGs shared by the male Tg and NTg have previously been linked to early-life stress, anxiety disorders and ADHD (*Erbin*) (49), sex-specific gene expression in ASD (79) and dopamine changes (*Erbb2ip, Neurod6*) (80), and to TH-dependent growth and embryonic differentiation processes (*Secisbp2l*) (50). Other pathways associated with hippocampal DEGs in the males involved mitochondrial energy metabolism. Dysfunction of mitochondria is widely accepted as a central feature of neurological diseases (81), several of which appear in the top five pathways associated with the DEGs in the hippocampus, and which share some disease symptoms with the behavioural changes we observed (82).

Whether these gene expression differences directly underpin the behavioural differences seen in Tg and NTg animals remains to be established. However, taken together, these shared differences in comparison to WT animals are strongly indicative of brain changes in response to the adverse maternal environment.

While both the transgenic and non-transgenic animals were clearly impacted by the adverse environment, some alterations were exclusive to the transgenic offspring. Transcriptional analysis of changes in the male hypothalamus suggested disturbances in synaptic function and neuronal development, and disruption of histone modifying enzymes in comparison to NTg and WT animals. Within the amygdala, most impacted in the female Tg animals, gene expression changes were associated with dopamine receptor activity. Amygdala response to dopamine has been shown to impact response to anxiety, stress and fear in mice (83–85), behaviours reflected in our animals. These changes were notably absent in the NTg females. *Ttr,* which encodes a transporter protein mediating transfer of thyroid hormone (Th), was expressed at significantly higher levels in male Tg offspring. While we cannot exclude a genetic effect of the transgene, the region-specific effects in the brain suggest sequential adversities of FGR and the adverse maternal environment experienced postnatally. The Hyp and Amyg are neural tissues that develop predominantly prenatally (86–88), and were disproportionally affected in the Tg animals which are asymmetrically growth restricted *in utero* (28). In contrast, the Hip and VS that continue to develop post-partum (88) were impacted in both Tg and NTg animals exposed to poor maternal care. Mapping of the developmental timelines (Supplementary Material, Fig. S4) demonstrates that proliferation of the Hyp and Amyg and synaptogenesis occur at the time when there are significant deficits in the placental spongiotrophoblast compartment (26,27) and when fetal growth is restricted in our model (28). This developmental timing is consistent with the Hyp and Amyg being particularly susceptible to FGR (89). Conversely, neuronal tissues such as the Hip and NAc of the VS continue to proliferate postnatally potentially rendering them more susceptible to adversities experienced in the postnatal period.

Overall, in all the behaviours tested, males exhibited a greater number of behavioural deficits compared to females (Fig. 6). Numerous epidemiological data similarly report a greater impact of early-life adversities on male offspring, with boys at increased risk of attention deficits, cognitive problems and externalizing behaviour and girls at increased risk of anxiety (90–92). The work presented here lends further supports the higher vulnerability of males to early-life adversity.

## Conclusions

In summary, elevated placental *PHLDA2* is a highly common alteration associated with FGR and low birth weight in humans. Here, we use a *Phlda2* overexpression model to identify alterations in the brains and behaviour of offspring, with the greatest impact for males. Most importantly, we attribute altered behaviour to the adverse maternal environment induced by placental endocrine insufficiency and not the gene change *per se* since both transgenic and non-transgenic offspring are impacted. This aspect of our work is particularly important because placental endocrine insufficiency can result from a wide range of environmental stressors in pregnancy with considerable implications for human health.

## Material and Methods

### Ethics statement

Animal studies and breeding were approved by the Universities of Cardiff ethical committee, performed under a UK Home Office project licence (RMJ; PPL 3003134) and abide by ARRIVE guidelines. At the end of the study, animals were euthanized as required by the UK Home Office.

### Experimental animals

Experimental animals were housed within an environmentally controlled facility (temp = 21 ± 2°C, humidity = 60 ± 5%, light:dark cycle 12:12h), in home cages (45 × 12 × 12 cm) containing sawdust, cardboard tube, transparent plastic tube, a wooden chew stick and two squares of bedding material. *Ad libitum* standard chow food (Formulab Diet 5008, TestDiet, UK) and tap water were available throughout the study, unless specified. Test subjects were obtained from breeding virgin 129S2/SvHsd (129) females (Envigo, UK) with *Phlda2*^+/+BACx1(129)^ (26) studs to generate *Phlda2*^+/+BACx1(129)^ transgenic (Tg) and *Phlda2*^+/+^ non-transgenic (NTg) experimental animals, respectively. Concurrently, fully wildtype control was obtained from breeding 129 females (Envigo, UK) with in-house bred 129 studs. Female mice were housed individually from embryonic day (E) 16.5 to litter and rear the pups to post-natal day (P) 21 before weaning. Genotyping was performed as described (26).

### Behavioural analysis

Animals were weaned at P21 and housed with sex-matched littermates in groups of four, consisting of either WT or 2:2 NTg:Tg individuals. Behavioural characterization was performed on three cohorts to probe behavioural traits associated with known adversity-driven outcomes (Fig. 1B; Supplementary Material, Table S1).

Tests were carried out during the light period (6 am–6 pm) by both male and female researchers using tunnel or open hand technique to reduce potential anxiety-related effects of researcher influence on behaviour (93,94). Unless stated, behavioural tests were recorded and videos blindly analysed using either automated tracking software (EthoVision XT 13, Noldus, Tracksys Ltd., UK, RRID:SCR_000441 and RRID:SCR_004074) (light/dark box test, three-chamber test, social odour discrimination and open field), or manually using Behavioural Observation Research Interactive Software (BORIS) (95) (direct social interaction, social propinquity, exploratory reluctance test).

### Direct social interaction

Interactions with an unfamiliar host were observed over 3 min as a measure of sociability (48). Duration of time spent following host, sniffing host, being followed by host, being attacked by host, self-grooming and freezing/immobile were analysed.

### Light/dark box

An adapted light/dark box test (37,96) was used to probe anxiety recording total time spent in, and the number of entries made to the anxiogenic chamber.

### Social propinquity

Anxiety-like and sociability behaviour outcome measures were taken from a social propinquity test (37,48,97). Briefly, two weight- and group-matched non-cage mate and unrelated mice were placed in a shared aversive arena for 60 min with access to a cardboard tube providing a single sheltered space. From recorded sessions latency to the first time both mice cohabited the tube, proportion of tube co-occupation time (sociability) and the proportion of tube vacant time (anxiety) were measured.

### Social odour discrimination

Response to unfamiliar social odour was assessed using the social odour discrimination task (48). Briefly, mice were presented three times with three sample odours for 2 min; first water (*W* (1)*, W* (2) and *W* (3)), secondly with a novel sex-mismatched home cage odour (*S1* (1)*, S1* (2) and *S1* (3)), and finally with a different novel sex-mismatched home cage odour (*S2* (1)*, S2* (2) and *S2* (3)). The difference in number of visits and time spent at the odour between the third presentation of the original social odour (*S1* (3)) and the first presentation of the novel social odour (*S2* (1)) were calculated using automated video tracking software and taken as a measure of sociability.

### Exploratory reluctance test

The propensity of individuals to explore novel social settings or remain within a familiar environment was used to assess anxiety. Briefly, cage-mates except for the test subject were removed from their home cage and all mice were removed from a second genotype- and sex-matched home cage. Cages without lids were placed end-to-end in a dimly lit room (<30 lux), experimenter left the room and cages were filmed for 10 min. Latency to first approach and time taken to cross into the novel cage were recorded.

### Three-chamber test

Outcome measures of the three-chamber test were used to assess sociability. As described (37,48,98), mice were placed into the middle of three identically sized chambers. The two adjoining chambers each housed an upturned wire mesh cage, one containing an unknown ‘host’ mouse (CD1, sex mis-matched), and the other left empty. The set-up was recorded for 5 min, with the total length of time spent in each chamber, number of entries and latency to enter measured.

### Operant testing

Cognitive testing was conducted using sound-attenuated operant boxes running a CCT and the five-choice serial reaction-time task (5CSRTT). Detailed operant protocols can be found in Supplemental methods S1. The number of magazine entries in the habituation period of CCT and total trials started in the 5CSRTT were analysed as motivation measures. Acquisition slope of conditioned stimulus (CS) response, mean number of consolidation phase CS responses and number of responses in an extinction test were analysed as cognitive measures in CCT. Accuracy, response time and number of time-outs in 5CSRTT were analysed as attentional measures. The number of anticipatory responses was analysed as a measure of impulsivity. Motivation and perceived reward value of the strawberry milkshake was measured in a consumption test (99) performed following the final day of operant testing, as detailed in Supplemental methods S2.

### Locomotor activity

Movement activity was recorded over 32 h in clear Perspex cages (42 × 26 × 19 cm, Med Associates, St. Albans VT, USA) with three infrared (IR) beams crossing the width of the cage at 10 cm intervals. Mice were individually housed with powdered food and free access to water. Following a 6-h habituation period (12 pm–6 pm), movement was quantified by automated counting of non-preservative beam breaks (MED-PC IV software) for the 12 h dark period (6 pm–6 am) and the 12 h light period (6 am–6 pm).

### Open field

Distance moved, movement time and velocity within an open field arena were taken as measures of activity. Within a dimly lit room (<30 lux) mice were habituated in home cage groups to a square Perspex arena (80 × 80 cm) within a larger arena with opaque sides for 5 min. Later the same day mice were individually placed in the centre of the arena and tracked for 15 min using an overhead camera and Ethovision software (Version 2.3.19, Noldus Information Technology, The Netherlands).

### Analysis of FCMs

Concentrations of metabolites of the stress hormone corticosterone were used as a non-invasive measure of stress under baseline and stressed conditions, as described previously (48). Briefly, fecal samples were collected from individually housed mice over a period of 3 h after being left unhandled for 48 h (baseline) and following overnight exposure to a stressor (fox odour) in their home cages. FCMs were quantified with a 5a-pregnane-3b,11b,21-triol-20-one enzyme immunoassay.

### Lickometry

Depression-like behaviours were examined by measuring hedonic response to increasing sucrose reward concentrations through lickometry testing, as described (100). Detailed lickometry protocols can be found in Supplemental methods S3. Total number of licks was analysed as a motivational measure. LCS and response to increased sucrose concentration were analysed as depression-related measures.

### RNA sequencing analysis

*N* = 3 per group of P21 male and female hypothalamus (Hyp), ventral striatum (VS), hippocampus (Hip) and amygdala (Amyg) were dissected into RNA*later* (Sigma, UK (R0901)). RNA was prepared using RNA binding columns (GenElute Mammalian Total RNA Miniprep Kit, Sigma, UK (RNB100-100RXN)). A total of 200–500 ng of RNA (1 × 75 bp) was analysed at a depth of 30 million reads. Paired-end reads from Illumina sequencing were trimmed with Trimmomatic (101), assessed for quality using FastQC (Babraham Bioinformatics), mapped to mouse GRCm38 using STAR (102) and counts assigned to transcripts using featureCounts (103) with the GRCm38.84 Ensembl gene build GTF. Differential expressed genes (DEGs; adj.pval < 0.05, Benjamini-Hochberg correction for multiple testing) were identified using DESeq2 package (104). Pairwise differential analyses of gene counts normalized to brain region were performed to provide lists of DEGs between Tg vs NTg, NTg vs WT and Tg vs WT of adj.pval < 0.01. DEGS, within a significance threshold of adj.pval < 0.05, shared between the ‘Tg vs WT’ and ‘Tg vs NTg’ comparisons, and those shared between the ‘Tg vs WT’ and ‘NTg vs WT’ comparisons were analysed using EnrichR (105,106). Note: the male and female RNAsequencing was performed at different times.

### Statistics

A unified scoring method (48) was used to combine outcome measures probing similar behavioural traits. Outcome measure data for each test were normalized within sex to obtain a ‘measure score’ between 0 (low indication of that particular behaviour) and 1 (greatest indication of that behaviour) for each individual using the formula;

$$ X(i)=\frac{M(i)}{M(m)} $$

where X(i) = normalized individual measure score, M(i) = actual individual measure datum [e.g. time spent in light (s)], and M(m) = maximum measure datum in study cohort (split by sex). Negative measure data values were assigned a score of 0, and time-out scores (e.g. failure to enter light) received a latency measure score of 1. For measures determined to be negatively associated with a behaviour (e.g. time spent in light and ‘anxiety’), the measure score was subsequently inverted using the formula below, thus ensuring a greater score in any measure related to an increase in the specified behaviour.

$$ X(i)=1-\frac{M(i)}{M(m)} $$

Mean X(i) values of similar measures associated with a behavioural test (e.g. all anxiety-related measures of the light/dark box test) were calculated for each individual to give a ‘test score’, T(i), for that behavioural trait. Mean scores incorporating T(i) values from each behavioural test were calculated for individuals, giving a ‘unified score’ for each behavioural trait. This enabled all outcome measures of each test to contribute to the score, with each test having an equal influence on the final result. Datasets of raw, transformed and unified data are available (Figshare).

The proportion of individuals within experimental groups scoring greater than the cohort median for each behavioural trait was used summarize behavioural outcomes. The incidence of these high scoring individuals within the Tg and NTg groups was compared to that within the WT control population and described as a fold-change in the incidence of each behavioural trait.

Data were analysed using GenStat 19th edition software (VSN International, UK) using two-way ANOVA or repeated measures ANOVA where appropriate, with post hoc tests performed using SPSS version 25 (SSPS, USA) with Tukey’s or Bonferroni correction for multiple comparisons. For conciseness, only main significant findings are described in the main text. Full statistical results are presented as Supplementary Material, Table S4. Supplemental Data include three figures, four tables and supplemental methods.

## Supplementary Material

Supplemental_information_NEW_ddab154

## Web resources

Links to RNA sequencing data to be included.
