# Supplementary material for: Placental endocrine insufficiency programs anxiety, deficits in cognition and atypical social behaviour in offspring
Source: Hum Mol Genet. 2021 Jun 7;30(19):1863–80. doi: 10.1093/hmg/ddab154 (PMC8444454; doi:10.1093/hmg/ddab154)
Supplement: Supplemental_information_NEW_ddab154 [file supplemental_information_new_ddab154.docx]

**Supplemental methods**

**Supplemental methods S1** Operant testing

Cognitive testing was conducted using 12 modified sound-attenuated operant boxes controlled using a BehaviourNet Controller BNC MKII operating system (Campden Instruments, Loughborough, UK). Chambers of dimensions 140 x 135 x 135 mm were constructed of four aluminium walls and a clear Perspex lid. The back wall of each chamber held an array of 9 nose-poke holes containing a bulb to illuminate the hole and an infrared sensor to detect nose entries into the hole. The opposing wall contained a reward magazine, also with a light bulb and infrared sensor, into which liquid strawberry milkshake (Yazoo, FrieslandCampina, Amersfoort, Netherlands) was dispensed via a peristaltic pump. An additional nose-poke hole with bulb and sensor was located on each side of the magazine and 2 house-light bulbs were located at the top of the side wall panels to illuminate the chamber.

For 5 days prior to introducing the mice to the operant chambers, a graduated water restriction regime was introduced, reducing free access to water to 3 hrs per day. During this time, the strawberry milkshake was presented in their home cages to reduce any neophobic response to the reward.

Classical Conditioning Task (CCT)

All nose-poke holes were blockaded for the entirety of the classical conditioning task. On the first operant testing day mice underwent a habituation session of 20 minutes, for which a 10-second (500 µl) pulse of milkshake reward was presented in the magazine at the start of the session, with the house-light illuminated throughout. Mice were able to explore the chamber and retrieve the reward freely from the magazine. The total number of magazine entries was recorded.

CCT consisted of 10 consecutive daily sessions consisting of 15 trials. Each trial began with an inter-trial interval (ITI) of pseudo-random length (60, 75, 90, 105 or 120 seconds), during which all lights were turned off. Following the ITI, the house-light (conditioned stimulus (CS)) was illuminated for 10 seconds, followed directly by a 250 ms (12.5 µl) pulse of reward into the magazine. The house-light would remain on until the reward was retrieved, triggering a 5-second delay before the start of the next trial. The delay timer would reset if an animal returned to the magazine within the 5-second wait period. The number of entries to the magazine were recorded in 2-second bins during the last 10 s of each ITI and the 10 s of each stimulus presentation. In addition, the number of magazine entries made during the wait period was recorded. A final extinction session was conducted under the same conditions as described above, but with the milkshake delivery tube removed from the reward pot ensuring no reward was delivered to the chamber.

Sessions 1 to 5 of the CCT were designated as the ‘acquisition phase’, during which the mice learn to associate the CS with the reward. The development of the CS-reward association was assessed by plotting the difference between the number of CS and baseline pokes per session, using the formula:

$$Total Conditioned Pokes=CS\left( n \right)-ITI(n)$$

where $CS\left( n \right)$ = the total number of magazine entries made during the 10s CSs (total 150 seconds) and $ITI\left( n \right)$= the total number of magazine entries made during the final 10s of the ITIs (total 150 seconds). The slope of the line for these days was taken as the rate of learning and was calculated using the following formula:

$$m\left( i \right)=\frac{r(i)\times sy(i)}{sx(i)}$$

Where $\left( i \right)$ = individual animal data set, $m\left( i \right)$ = slope, $sy\left( i \right)$= standard deviation of y values (*Total Conditioned Pokes*), $sx\left( i \right)$= standard deviation of the x values (session number), and $r\left( i \right)$= correlation between x and y values calculated using the formula: $r\left( i \right)$= $\frac{covariance x\left( i \right) and y(i)}{sx\left( i \right) \times sy(i)}$.

Sessions 6 to 10 were defined as the ‘efficiency learning phase’ whereby the mice learn that a reduction of the number of visits to the magazine during the CS amounts to the same reward outcome. The mean Total conditioned pokes over these sessions was used to assess the strength of the CS-reward association.

An extinction session used to probe cognitive flexibility measured how the mice respond to learning a new association, CS ≠ reward. Extinction values were calculated as $Total CS pokes$ made during the presentation of the CS of the extinction test (total 150 s) was compared to the total number of entries made in during the CS of the final CCT session (150 s).

Operant conditioning

Following completion of the CCT, the blockades were removed to open holes ‘L’ and ‘R’, and mice were trained to respond in the illuminated holes using a fixed ratio response program. Both lateral holes would remain illuminated until a response was made in either, whereby they would be extinguished, the magazine light illuminated and a 50 µl reward delivered. The magazine would remain illuminated until the reward was collected, whereby a 2-second ITI period with all light off would precede the beginning of the next trial. To encourage the mice to explore the illuminated hole, milkshake was painted around and into the back. Once mice began initiating hole pokes by themselves no further ‘painting’ was needed. The performance criteria for passing onto the next training phase was set at 50 completed trials in a 20-minute session, or 10 sessions if 50 trials were not completed. Subsequently, mice were trained to poke in the centre nose-poke hole ‘C’ at the back of the chamber, opposite the reward magazine, with a program design as above with blockades replaced on hole ‘L’ and ‘R’ and removed from hole ‘C’. Performance criteria for this phase was set at ≥100 responses within two consecutive daily 20-minute sessions, or 10 sessions if 100 responses were not completed. Any mice that achieved criteria before the tenth session were removed from training until the final session.

Five-Choice Serial Reaction Time Task (5CSRTT)

For the 5CSRTT, hole blockades were removed from holes ‘A’, ‘B’, ‘C’, ‘D’ and ‘E’. Mice received an introduction session of 30 mins whereby one of the 5 open holes was illuminated, in a pseudo-random order, for 10 seconds. A response into the correct hole would trigger the light to extinguish, the magazine to illuminate and a 25 µl reward to be delivered. Retrieval of the reward triggered a 2-second ITI period during which all lights were extinguished, before the next trial was initiated. A response to an incorrect hole, or failure to respond within a 30 second limited hold time resulted in a 5-second time-out (TO) period, during which all lights were extinguished, before initiation of the next trial. Subsequently, mice underwent a series of attentional probes whereby the time for which the stimulus light was illuminated was progressively reduced (5 days at 10 seconds, 5 days at 2 seconds, and 5 days at 1 second). Performance was assessed through the following measures; Total trials started (TTS), response accuracy ($=\left( \frac{Correct responses}{Correct + Incorrect responses} \right)$), time taken to make a correct response (RT), number of time-outs incurred (TO), and number of anticipatory pokes made during the ITI or TO periods.

All animals including those that did not reach training criteria continued to undergo the full testing for the duration of the experiment. Sessions for which <100 trials were completed were removed from the analyses.

**Supplemental methods S2** Consumption test

Motivation and perceived reward value of the strawberry milkshake was measured in a consumption test[1] performed for two consecutive days following the final day of operant testing. Water restriction was maintained, and mice were placed within the operant boxes as before for 30 mins. A 2.5 cm diameter plastic petri dish (Fisher Scientific, United Kingdom, RRID:SCR_008452) was filled with strawberry milkshake reward, weighed, and fixed to the floor of the operant box to allow free access. The house light was illuminated for the duration of the test. At the end of each session the dishes were re-weighed to obtain the amount consumed. A mean value of the two sessions was calculated and adjusted for subject weight using the following formula:

$$amount consumed =\left( \frac{g^{1}+g^{2}}{2} \right)/{wt}$$

where $g^{1}$ and $g^{2}$ = weight in grams of milkshake consumed in first and second session, respectively, $wt$= weight in grams of subject.

**Supplemental methods S3** Lickometry testing

Mice were trained and tested in custom-made acrylic drinking chambers (32 x 15 x 12 cm, Med Associates) with steel mesh floor and lid holding a 50 ml falcon tube attached to a stainless-steel drinking spout. A contact-sensitive lickometer connected to a PC running MED-PC software (Med Associates) was used to record individual licks made to the nearest 0.01 s. Prior to training, mice were placed on an incremental water-restricted regime which culminated with water being removed from home cages at 5 pm and returned post-testing the following morning, allowing continuous free access for a minimum of 6 hrs per day. On the first training day mice were placed in individual test chambers for 30 mins with the drinking spout extended fully into the chamber allowing access to tap water. The following day, the spouts for the water were retracted until they were flush with the lid so contact could only be made with the tongue, and the session time reduced to 15 mins. Mice were trained in this way for 4 daily sessions until all mice were licking consistently. Subsequently, mice were switched to a food-restricted protocol in which food was removed from the home cages at 5pm and returned post-testing the following morning, allowing continuous free access for a minimum of 6 hrs per day. Habituation to sucrose solution was performed over nine 15-minute sessions using a 10% w/v concentration of sucrose in tap water, until mice were consistently performing >40 licks. The testing phase was conducted using 4% and 16% w/v sucrose solutions, with mice undergoing three consecutive daily sessions with each concentration, and the order of presentation counterbalanced across groups. The mean lick cluster size (LCS) for each sucrose concentration, and the slope of the increase in response are presented. LCS was defined as the number of licks in a series in which the inter-lick interval (ILI) did not exceed 500 ms, and a pause between licks of ≥500 ms would indicate a separate lick cluster[2]. Test session data of individuals from which <100 licks were recorded were excluded from analysis.

**Supplemental References**

1. Forbes NF, Stewart CA, Matthews K, Reid IC. Chronic mild stress and sucrose consumption: validity as a model of depression. Physiol Behav. 1996;60(6):1481-4. Epub 1996/12/01. doi: 10.1016/s0031-9384(96)00305-8. PubMed PMID: 8946494.

2. Davis JD, Smith GP. Analysis of the microstructure of the rhythmic tongue movements of rats ingesting maltose and sucrose solutions. Behav Neurosci. 1992;106(1):217-28. PubMed PMID: 1554433.

**Supplemental figures**

**Supplemental Figure S1 Fecal corticosterone metabolite analysis:** *N* = WT(F), 11; NTg(F), 11; Tg(F), 12; WT(M), 12; NTg(M), 12; Tg(M), 12. Baseline concentration, concentration under stressed conditions and percentage change in of corticosterone metabolites. Tg mice had a lower baseline concentration of corticosterone metabolites compared to WT (*p<0.05). No differences were observed in the stressed state or the percentage change in concentration between baseline and stressed states. Error bars represent ±SEM.


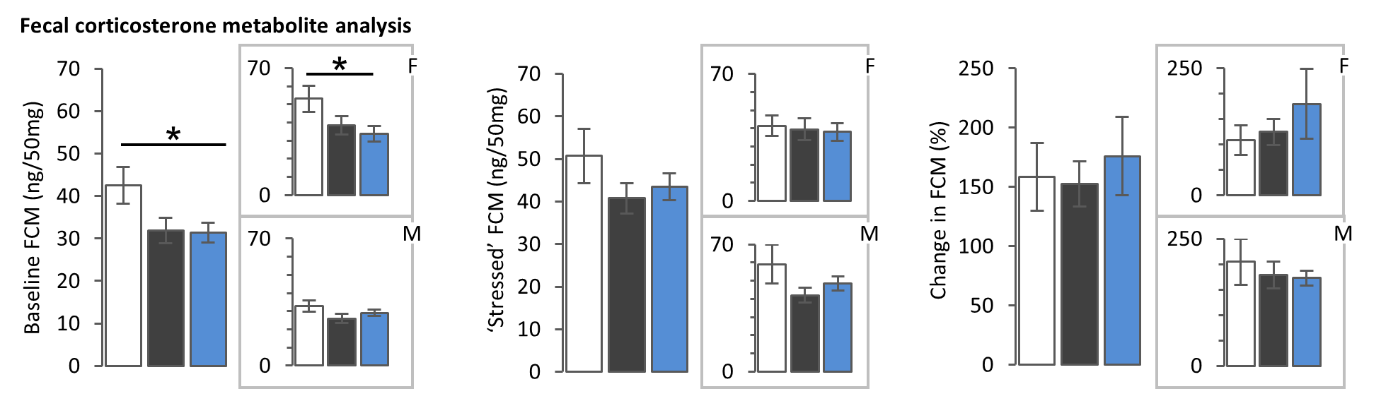


**Supplemental Figure S2 Three-chamber test:** *N* = WT(F), 20; NTg(F), 20; Tg(F), 19; WT(M), 20; NTg(M), 20; Tg(M), 20. Mean number of crosses into and time spent within the empty and occupied chambers, and latency to enter the occupied chamber. No difference in number of crosses into the empty chamber was observed. Compared to males, females visited the occupied chamber more than males (^$^p<.05), spent a greater amount of time in the empty chamber (^$$^p<.01) and less in the occupied chamber (^$^p<.05). Males were quicker to first enter the occupied chamber compared to females (^$$^p<.01). Error bars represent ±SEM


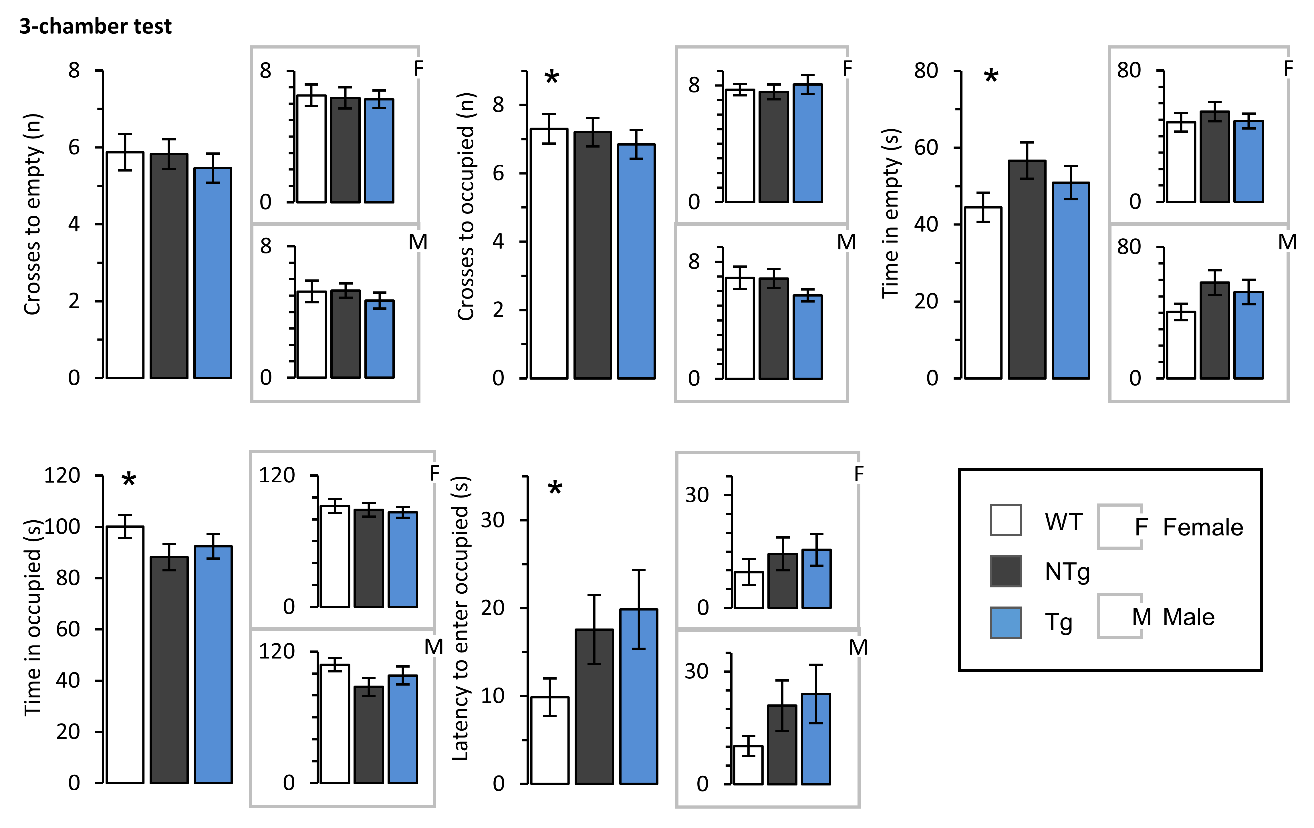


**Supplemental Figure S3 (A) Open field:** *N* = WT(F), 12; NTg(F), 12; Tg(F), 12; WT(M), 12; NTg(M), 12; Tg(M), 12. Total distance moved, total movement time and velocity. No differences in any measure were observed. **(B) Locomotor activity:** *N* = WT(F), 30; NTg(F), 27; Tg(F), 32; WT(M), 32; NTg(M), 31; Tg(M), 31. Total non-perseverative beam breaks in the dark period 6pm – 6am (
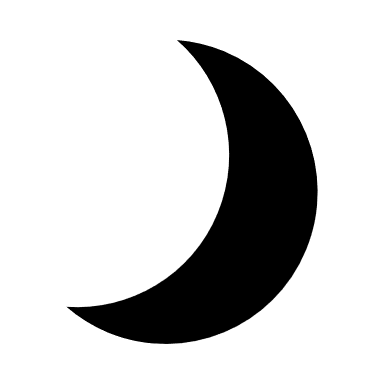
), and light period 6am – 6pm (
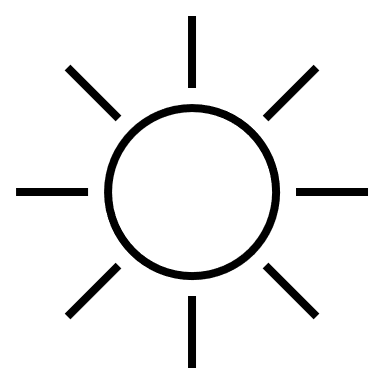
). No differences in activity were found. Error bars represent ±SEM.


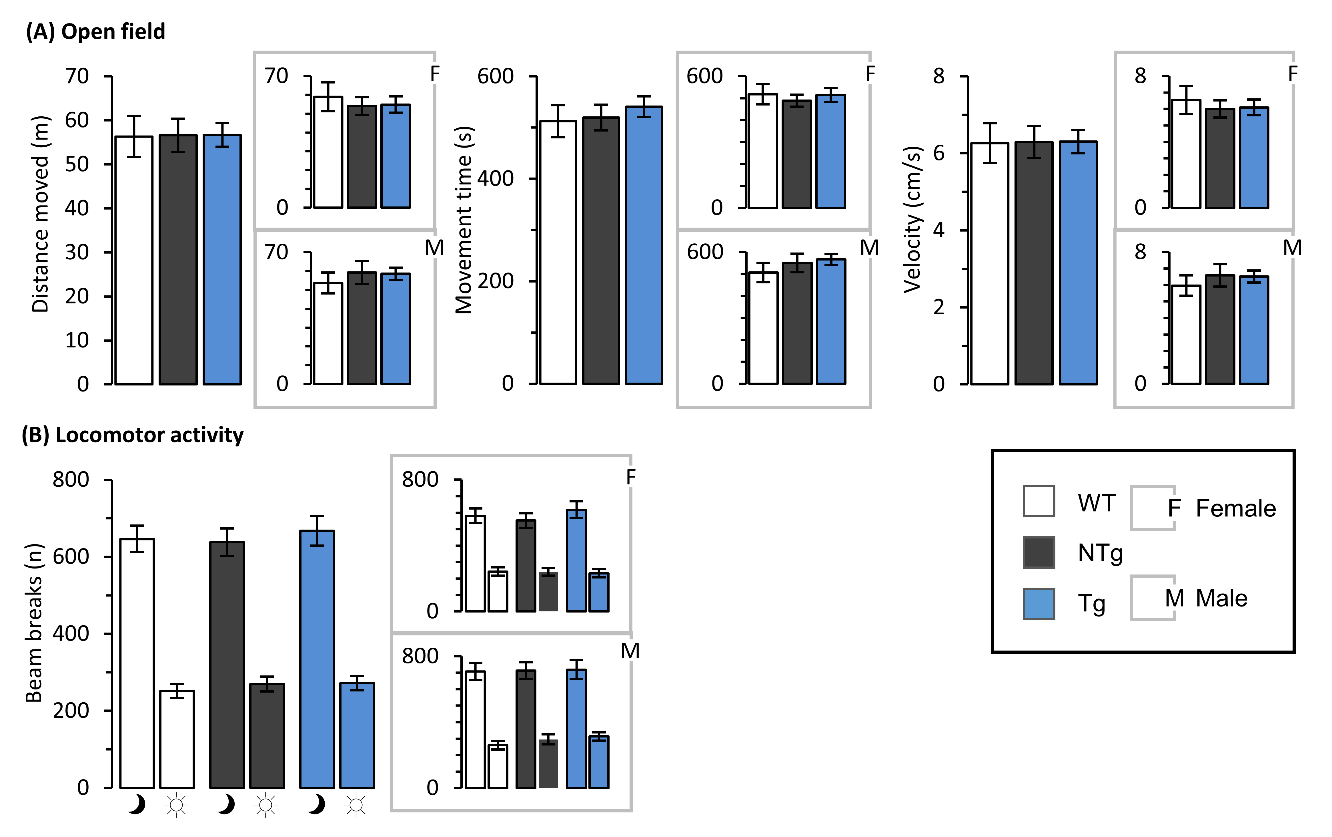


**Supplemental Figure S4 Developmental timeline of placenta and fetal brain:** Timeline demonstrating the proliferation of tissues, cells, and processes in the pre- and early post‑natal period in the developing mouse brain. Adapted from (86).

**
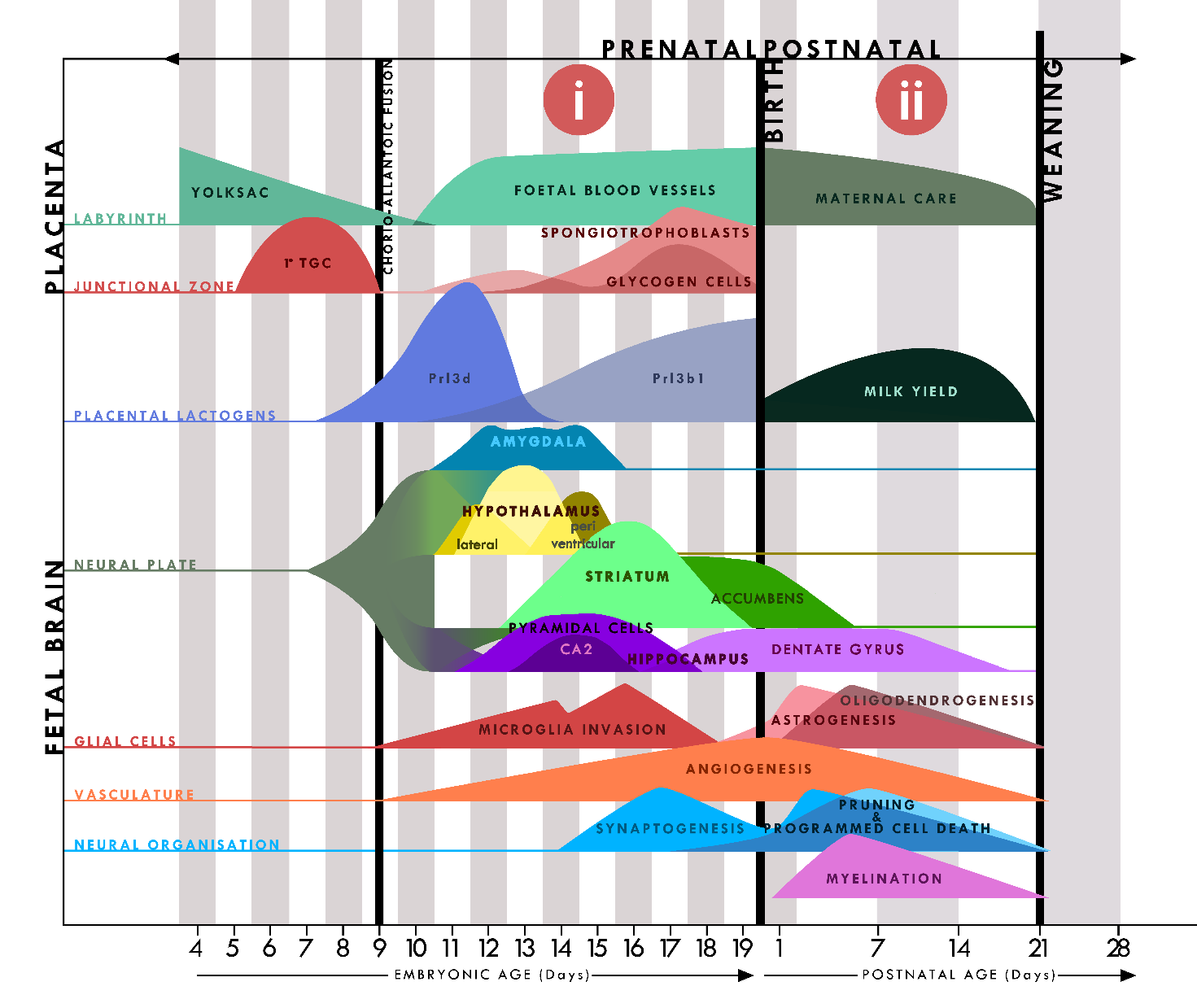
**

**Supplemental tables**

**Supplemental Table S1** Behavioural testing

**List of tests performed for behavioural cohorts presented in order of testing.** Including test name and respective outcome measures, the associated behavioural traits probed, and direction of influence each measure contributes to the unified behavioural scores. A positive influence (+ve) is assigned to measures which *increase* as a tendency towards the behavioural trait it is probing increases (e.g. ‘Accuracy’ in the 5CSRTT and ‘cognition’ score). A negative influence (-ve) is assigned to measures which *decrease* as a tendency towards the behavioural trait it is probing increases (e.g. ‘Time in light’ in the light/dark box and ‘anxiety’ score). **CS** = conditioned stimulus (Classical conditioning). **FCM** = faecal corticosterone metabolites. **LCS**= lick cluster size (Lickometry).

| **Cohort 1 (*N*=20)** |  |  |  |
| --- | --- | --- | --- |
| **Test** | **Measure** | **Behavioural trait** | **Influence** |
| Direct social interaction  (Age 4 weeks) | - Time following host  - Time being followed  - Time sniffing host  - Time self-grooming  - Time attacking host  - Time being attacked  - Time immobile | Sociability  Sociability  Sociability  Sociability  Sociability  Sociability  Sociability | +ve  +ve  +ve  -ve  +ve  +ve  -ve |
| Light/dark box  (Age 8 weeks) | - Crosses into light  - Time in light | Anxiety  Anxiety | -ve  -ve |
| Social propinquity  (Age 9-12 weeks) | - Latency to first share  - Time vacant  - Duration of double occupancy | Sociability  Anxiety  Sociability | -ve  -ve  +ve |
| Social odour discrimination  (Age 13 weeks) | - Increase in frequency of visits to novel social odour  - Increase in time spent sniffing novel social odour | Sociability  Sociability | +ve  +ve |
| Exploratory reluctance test  (Age 14-15 weeks) | - Latency to first approach  - Latency to first cross | Anxiety  Anxiety | +ve  +ve |
| 3-chamber test  (Age 20-22 weeks) | - Crosses into empty  - Crosses into occupied  - Time in empty  - Time in occupied  - Latency to enter occupied  - Mean time in occupied | Sociability  Sociability  Sociability  Sociability  Sociability  Sociability | -ve  +ve  -ve  +ve  -ve  +ve |
|  |  |  |  |
| **Cohort 2 (*N*=12)** |  |  |  |
| **Test** | **Measure** | **Behavioural trait** | **Influence** |
| Classical conditioning  (Age 8 -9 weeks) | - Entries during habituation  - CS learning  - CS efficiency learning  - Extinction learning | Motivation  Cognition  Cognition  Cognition | +ve  +ve  +ve  -ve |
| Five-choice serial reaction time task  (Age 10 - 21 weeks) | - Total trials started  - Number of time outs  - Accuracy  - Response time  - Number anticipatory pokes | Motivation  Attention  Attention  Attention  Impulsivity | +ve  -ve  +ve  -ve  +ve |
| Consumption test  (Age 22 weeks) | - Amount consumed | Motivation | +ve |
| Locomotor activity  (Age 23-24 weeks) | - Dark period activity  - Light period activity | Activity  Activity | +ve  +ve |
| Open field  (Age 25 weeks) | - Distance moved  - Movement time  - Velocity | Activity  Activity  Activity | +ve  +ve  +ve |
| Faecal corticosterone metabolites (FCMs)  (Age 26 weeks) | - Baseline FCM levels  - ‘Stressed’ FCM levels  - Change in FCM levels | Stress  Stress  Stress | +ve  +ve  +ve |

| **Cohort 3 (*N*=20)** |  |  |  |
| --- | --- | --- | --- |
| **Test** | **Measure** | **Behavioural trait** | **Influence** |
| Lickometry  (Age 13 - 20 weeks) | - Total number of licks  - Baseline lick cluster size (LCS)  - LCS with increased value reward  - Change in LCS | Motivation  Depression  Depression  Depression | +ve  -ve  -ve  -ve |
| Locomotor activity  (Age 21-23 weeks) | - Dark period activity  - Light period activity | Activity  Activity | +ve  +ve |

**Supplemental Table S2** RNAseq analysis

**(A)** Differentially expressed genes between male Tg and WT groups

| **Amygdala** | | | | | | |
| --- | --- | --- | --- | --- | --- | --- |
| **Gene name** | **Ensembl ID** | | **Fold change** | **Log2 fold change** | **pval** | **adj.pval** |
| Ttr | ENSMUSG00000061808 | | 186.11 | 7.54 | 1.07E-10 | 8.22E-07 |
| **Hypothalamus** | | | | | | |
| **Gene name** | | **Ensembl ID** | **Fold change** | **Log2 fold change** | **pval** | **adj.pval** |
| Zfr2  Lrrc16b  Bzrap1  Zgpat  Celsr3  Coro6  Zfp512b  Dot1l  Fcho1  Fam193b  Tmem191c  Col4a2  Gigyf1  PISD  Gm27998  Mir770  Crocc  Unc13a  Sema4g  Col4a1  Agrn  Vwa5b2  Ebf4  Pnn  Map4k2  Col16a1  Sptbn4  Ankrd24  Colgalt1  Mir5125  P3h3  Clasrp  Suv420h2  Col11a2  Rgs11  Mbd6  5031439G07Rik  Lime1  Sema6c  Cacna1g  Arhgef1  Plekhn1  Gm26953  Plxna3  Kmt2b  Arhgap33  Lrrc45  Ccdc84  Myo9b  Slc16a11  Adamts10  Inha  Zfp692  Rgl2  Nktr  Kifc2  A230050P20Rik  Pisd-ps1  Gcn1l1  Tle2  Speg  Smpd4  Setd1a  Atxn7l2  Zswim8  Cpsf4  Man2c1  Rps6kb2  Ece2  4933439C10Rik  Lmtk3  Gpc1  Dennd6b  Zfp523  Kcnt1  Leng8  Prpf40b  Pxn  Tbkbp1  Thbs3  Clk2  Adam11  Hdac7  Gm27882  Dgkz  Gm13563  Abcc5  Zfp384  Sfswap  Ssh3  Akap8l  Srrm2  Ncln  Cacna1h  Atxn2l  RP23-325D10.3  Zfp598  Pnisr  Gm996  Repin1  Snapc4  Gm26702  Tjap1  Slc4a3  Srrm1  Safb2  Pcgf2  Mapk15  B4galnt4  Nrbp2  Zfp57  Ccnl2  Ercc2  Celf3  Epha10  Pkd1  Flna  Klhl17  Snrnp70  Mroh1  Vps9d1  Uckl1  Ring1  Gm20521  Pabpn1  Dmpk  Cbx7  Tmem181b-ps  Ptpn23  Fbf1  Dus3l  Shkbp1  Dcaf15  Vip  Crtc2  Ltbp4  Dusp15  Ap1g2  Trank1  Phldb1  Phf1  Pnrc2  Gga1  Adam15  Gm20605  Firre  Cc2d1a  Cyth1  Csf2ra  Arhgef40  BC037034  Fbxl19  Malat1  Rap1gap  Cpt1c  Begain  Scrib  Rbm5  Ttc4  Lrch4  1700047M11Rik  Gm3764  Sirt7  Edc4  Dazap1  Whamm  Dync1h1  Klc4  Syne4  Phc1  Josd2  Gm27032  Tmem145  Spaca6  Calb1  Vegfa  Arrb2  Traf7  Ptpru  Chfr  Kndc1  Rfx1  Dstn  Pltp  Slc9a5  Gm13562  Acap3  Mafg  Mir1906-1  Gtpbp2  Miat  Pced1a  Fbrs  Cpne7  Acaa1a  Usp19  Atp8b2  9430015G10Rik  Gramd1a  B3gat1  Npr2  Clcn2  Chn1  Ankrd13b  Eng  Igkc  Gm26377  Ppox  Shisa7  Trim39  Srsf5  Dbn1  Zfyve27  Mink1  Dnase2a  Nckap5l  Gm25994  Trim11  Matk  Lig1  Rsrp1  Dvl1  Dusp11  Mettl17  Trmt1  Ccdc9  Meg3  Phf2os1  Snhg20  Bmp1  Wsb1  Rimbp2  Acin1  Mir3072  Zfp335  Amfr  Letm2  Mau2  Amy1  Zmym3  Fam214b  Izumo4  Slc18a2  Tia1  Ssr3  Clk3  Srek1  Megf8  Clk1  AW209491  Supt20  Atg2a  Cdk9  Car11  Appl2  Pnma3  Zmym6  Vars  Mapk11  Zcchc7  Mir3101  Pdlim7  Ube2e3  Gm22357  Cep131  Ddx17  Hsf1  Noc2l  Usp21  Morf4l1  Abhd14b  Impa1  Adck5  Micall1  Abtb1  Cpsf7  Limk2  Pid1  Sap25  Ppfia4  Serpini1  Dlk2  Gdpd2  Rnf123  Mapkbp1  Iglon5  Mier2  Ciz1  Cpne6  Ankzf1  Fmnl1  Rdh13  Odf2  Pam  Adgrb1  Rpl15  Sin3b  Gm16740  Ankrd46  Cc2d1b  Hook2  Atat1  Rap2a | | ENSMUSG00000034949  ENSMUSG00000022211  ENSMUSG00000034156  ENSMUSG00000027582  ENSMUSG00000023473  ENSMUSG00000020836  ENSMUSG00000000823  ENSMUSG00000061589  ENSMUSG00000070000  ENSMUSG00000021495  ENSMUSG00000055692  ENSMUSG00000031503  ENSMUSG00000029714  ENSMUSG00000095041  ENSMUSG00000098395  ENSMUSG00000076451  ENSMUSG00000040860  ENSMUSG00000034799  ENSMUSG00000025207  ENSMUSG00000031502  ENSMUSG00000041936  ENSMUSG00000046613  ENSMUSG00000053552  ENSMUSG00000020994  ENSMUSG00000024948  ENSMUSG00000040690  ENSMUSG00000011751  ENSMUSG00000054708  ENSMUSG00000034807  ENSMUSG00000092981  ENSMUSG00000023191  ENSMUSG00000061028  ENSMUSG00000059851  ENSMUSG00000024330  ENSMUSG00000024186  ENSMUSG00000025409  ENSMUSG00000036046  ENSMUSG00000090077  ENSMUSG00000038777  ENSMUSG00000020866  ENSMUSG00000040940  ENSMUSG00000078485  ENSMUSG00000097986  ENSMUSG00000031398  ENSMUSG00000006307  ENSMUSG00000036882  ENSMUSG00000025145  ENSMUSG00000043923  ENSMUSG00000004677  ENSMUSG00000040938  ENSMUSG00000024299  ENSMUSG00000032968  ENSMUSG00000037243  ENSMUSG00000041354  ENSMUSG00000032525  ENSMUSG00000004187  ENSMUSG00000038884  ENSMUSG00000082286  ENSMUSG00000041638  ENSMUSG00000034771  ENSMUSG00000026207  ENSMUSG00000005899  ENSMUSG00000042308  ENSMUSG00000048997  ENSMUSG00000021819  ENSMUSG00000029625  ENSMUSG00000032295  ENSMUSG00000024830  ENSMUSG00000022842  ENSMUSG00000072893  ENSMUSG00000062044  ENSMUSG00000034220  ENSMUSG00000015377  ENSMUSG00000024220  ENSMUSG00000058740  ENSMUSG00000035545  ENSMUSG00000023007  ENSMUSG00000029528  ENSMUSG00000038517  ENSMUSG00000028047  ENSMUSG00000068917  ENSMUSG00000020926  ENSMUSG00000022475  ENSMUSG00000099235  ENSMUSG00000040479  ENSMUSG00000085767  ENSMUSG00000022822  ENSMUSG00000038346  ENSMUSG00000029439  ENSMUSG00000034616  ENSMUSG00000002625  ENSMUSG00000039218  ENSMUSG00000020238  ENSMUSG00000024112  ENSMUSG00000032637  ENSMUSG00000108815  ENSMUSG00000041130  ENSMUSG00000028248  ENSMUSG00000029419  ENSMUSG00000052751  ENSMUSG00000036281  ENSMUSG00000097872  ENSMUSG00000012296  ENSMUSG00000006576  ENSMUSG00000028809  ENSMUSG00000042625  ENSMUSG00000018537  ENSMUSG00000063704  ENSMUSG00000055629  ENSMUSG00000075590  ENSMUSG00000036036  ENSMUSG00000029068  ENSMUSG00000030400  ENSMUSG00000028137  ENSMUSG00000028876  ENSMUSG00000032855  ENSMUSG00000031328  ENSMUSG00000078484  ENSMUSG00000063511  ENSMUSG00000022558  ENSMUSG00000001062  ENSMUSG00000089917  ENSMUSG00000024325  ENSMUSG00000092232  ENSMUSG00000022194  ENSMUSG00000030409  ENSMUSG00000053411  ENSMUSG00000096780  ENSMUSG00000036057  ENSMUSG00000020776  ENSMUSG00000007603  ENSMUSG00000089832  ENSMUSG00000037103  ENSMUSG00000019772  ENSMUSG00000027936  ENSMUSG00000040488  ENSMUSG00000042662  ENSMUSG00000040701  ENSMUSG00000062296  ENSMUSG00000048537  ENSMUSG00000024193  ENSMUSG00000028675  ENSMUSG00000033128  ENSMUSG00000028041  ENSMUSG00000029720  ENSMUSG00000085396  ENSMUSG00000036686  ENSMUSG00000017132  ENSMUSG00000059326  ENSMUSG00000004562  ENSMUSG00000036948  ENSMUSG00000030811  ENSMUSG00000092341  ENSMUSG00000041351  ENSMUSG00000007783  ENSMUSG00000040867  ENSMUSG00000022568  ENSMUSG00000032580  ENSMUSG00000025413  ENSMUSG00000093445  ENSMUSG00000100147  ENSMUSG00000097156  ENSMUSG00000025138  ENSMUSG00000036270  ENSMUSG00000069565  ENSMUSG00000045795  ENSMUSG00000018707  ENSMUSG00000003546  ENSMUSG00000019737  ENSMUSG00000040669  ENSMUSG00000038695  ENSMUSG00000098051  ENSMUSG00000043843  ENSMUSG00000080316  ENSMUSG00000028222  ENSMUSG00000023951  ENSMUSG00000060216  ENSMUSG00000052752  ENSMUSG00000028909  ENSMUSG00000014668  ENSMUSG00000066129  ENSMUSG00000031706  ENSMUSG00000015932  ENSMUSG00000017754  ENSMUSG00000014786  ENSMUSG00000086779  ENSMUSG00000029033  ENSMUSG00000051510  ENSMUSG00000084535  ENSMUSG00000023952  ENSMUSG00000097767  ENSMUSG00000037773  ENSMUSG00000042423  ENSMUSG00000034796  ENSMUSG00000036138  ENSMUSG00000006676  ENSMUSG00000060671  ENSMUSG00000059939  ENSMUSG00000001248  ENSMUSG00000045994  ENSMUSG00000028469  ENSMUSG00000022843  ENSMUSG00000056486  ENSMUSG00000037907  ENSMUSG00000026814  ENSMUSG00000076609  ENSMUSG00000076138  ENSMUSG00000062729  ENSMUSG00000053550  ENSMUSG00000045409  ENSMUSG00000021134  ENSMUSG00000034675  ENSMUSG00000018820  ENSMUSG00000020827  ENSMUSG00000003812  ENSMUSG00000023009  ENSMUSG00000093133  ENSMUSG00000020455  ENSMUSG00000004933  ENSMUSG00000056394  ENSMUSG00000037266  ENSMUSG00000029071  ENSMUSG00000030002  ENSMUSG00000004561  ENSMUSG00000001909  ENSMUSG00000041375  ENSMUSG00000021268  ENSMUSG00000086889  ENSMUSG00000086859  ENSMUSG00000022098  ENSMUSG00000017677  ENSMUSG00000029420  ENSMUSG00000022185  ENSMUSG00000093351  ENSMUSG00000039834  ENSMUSG00000031751  ENSMUSG00000037363  ENSMUSG00000031858  ENSMUSG00000074264  ENSMUSG00000031310  ENSMUSG00000036002  ENSMUSG00000055862  ENSMUSG00000025094  ENSMUSG00000071337  ENSMUSG00000027828  ENSMUSG00000032316  ENSMUSG00000032621  ENSMUSG00000045039  ENSMUSG00000026034  ENSMUSG00000039182  ENSMUSG00000027751  ENSMUSG00000024773  ENSMUSG00000009555  ENSMUSG00000003273  ENSMUSG00000020263  ENSMUSG00000046287  ENSMUSG00000042408  ENSMUSG00000007029  ENSMUSG00000053137  ENSMUSG00000035649  ENSMUSG00000093348  ENSMUSG00000021493  ENSMUSG00000027011  ENSMUSG00000064816  ENSMUSG00000039781  ENSMUSG00000055065  ENSMUSG00000022556  ENSMUSG00000095567  ENSMUSG00000053483  ENSMUSG00000062270  ENSMUSG00000042073  ENSMUSG00000027531  ENSMUSG00000022550  ENSMUSG00000033039  ENSMUSG00000030083  ENSMUSG00000034820  ENSMUSG00000020451  ENSMUSG00000045658  ENSMUSG00000079165  ENSMUSG00000026458  ENSMUSG00000027834  ENSMUSG00000047428  ENSMUSG00000019359  ENSMUSG00000041528  ENSMUSG00000033902  ENSMUSG00000013367  ENSMUSG00000042570  ENSMUSG00000039205  ENSMUSG00000022212  ENSMUSG00000026199  ENSMUSG00000055805  ENSMUSG00000008435  ENSMUSG00000026790  ENSMUSG00000026335  ENSMUSG00000034730  ENSMUSG00000012405  ENSMUSG00000031622  ENSMUSG00000097167  ENSMUSG00000048307  ENSMUSG00000028582  ENSMUSG00000052566  ENSMUSG00000024426  ENSMUSG00000051615 | 1.51  1.84  1.89  1.91  1.78  3.29  1.66  1.82  1.67  2.37  1.58  1.88  1.86  2.41  2.59  2.98  1.85  1.46  1.81  1.81  1.51  1.80  1.64  1.72  1.69  2.31  1.60  1.89  1.46  3.06  2.12  2.30  2.23  2.11  2.44  2.09  1.56  2.12  1.64  1.73  1.91  2.27  2.89  1.50  1.38  1.58  2.14  2.12  1.76  2.12  2.19  2.06  2.17  2.02  1.67  1.42  1.47  2.47  1.40  2.09  1.51  2.02  1.42  1.79  1.38  2.06  1.60  2.12  1.44  2.19  1.46  1.54  1.74  1.73  1.60  2.20  1.63  1.77  1.73  1.76  1.66  1.38  1.96  2.78  1.55  1.49  1.52  1.56  1.59  1.99  1.97  1.64  1.43  1.41  1.64  1.45  1.50  1.83  1.34  1.40  1.81  1.34  1.83  1.37  1.39  1.78  1.60  1.84  1.32  1.66  2.18  1.73  1.75  1.40  1.58  1.50  1.62  1.62  1.75  1.34  1.41  1.60  1.61  1.70  1.86  2.22  1.49  1.86  1.50  1.40  1.54  2.23  1.58  -1.70  1.65  1.46  1.63  2.11  1.48  1.48  1.46  -1.38  1.37  1.46  1.61  1.87  1.30  1.38  1.74  1.44  1.38  1.35  2.13  1.25  1.28  1.39  1.51  1.56  1.57  1.44  2.00  2.04  1.90  1.26  1.49  1.58  1.24  1.64  2.58  1.25  1.74  2.34  1.50  2.14  -1.33  1.36  1.40  1.42  1.41  1.46  1.31  1.49  -1.35  1.27  1.53  1.81  1.48  1.45  2.61  1.47  2.75  1.33  1.36  1.53  1.58  1.27  1.25  1.47  1.48  1.23  1.36  1.49  -1.24  1.37  1.51  -4.64  -1.62  1.46  1.63  1.80  1.50  1.32  1.31  1.26  1.32  1.38  1.82  1.46  1.54  1.52  1.53  1.44  1.92  1.67  1.41  1.42  2.94  2.82  2.38  1.50  1.78  1.34  1.42  3.27  1.39  -1.23  1.52  1.35  2.06  1.29  1.27  1.48  -1.43  1.73  -1.28  1.42  1.37  1.18  1.89  -1.38  1.51  1.38  1.48  1.31  1.42  1.35  1.62  1.33  1.59  1.93  5.25  1.59  -1.35  3.05  1.43  1.43  1.33  1.44  1.31  -1.24  1.84  -1.32  1.57  1.30  1.47  1.27  1.32  -1.52  2.32  1.24  -1.36  1.63  1.53  1.32  1.49  1.40  1.38  1.42  1.27  1.64  1.32  1.34  1.39  -1.25  1.25  -1.32  1.22  -1.33  -1.30  1.46  1.68  1.26  -1.31 | 0.60  0.88  0.92  0.93  0.84  1.72  0.73  0.86  0.74  1.25  0.66  0.91  0.90  1.27  1.37  1.58  0.89  0.55  0.85  0.85  0.59  0.84  0.71  0.78  0.76  1.21  0.68  0.92  0.54  1.61  1.09  1.20  1.16  1.07  1.29  1.06  0.64  1.09  0.71  0.79  0.93  1.18  1.53  0.59  0.47  0.66  1.10  1.09  0.82  1.08  1.13  1.04  1.12  1.01  0.74  0.50  0.56  1.31  0.48  1.06  0.60  1.02  0.51  0.84  0.47  1.04  0.68  1.09  0.52  1.13  0.55  0.63  0.80  0.79  0.68  1.14  0.70  0.82  0.79  0.81  0.73  0.46  0.97  1.47  0.63  0.57  0.61  0.64  0.67  0.99  0.98  0.71  0.52  0.49  0.71  0.54  0.59  0.87  0.42  0.49  0.86  0.42  0.87  0.46  0.48  0.83  0.68  0.88  0.40  0.73  1.13  0.79  0.81  0.49  0.66  0.59  0.70  0.69  0.81  0.42  0.50  0.68  0.69  0.77  0.89  1.15  0.58  0.90  0.59  0.49  0.62  1.16  0.66  -0.76  0.73  0.55  0.70  1.08  0.56  0.57  0.55  -0.46  0.45  0.55  0.68  0.91  0.38  0.46  0.80  0.53  0.46  0.44  1.09  0.32  0.36  0.47  0.59  0.64  0.65  0.53  1.00  1.03  0.93  0.34  0.57  0.66  0.31  0.72  1.37  0.33  0.80  1.23  0.59  1.10  -0.41  0.44  0.48  0.51  0.49  0.54  0.39  0.58  -0.43  0.35  0.62  0.86  0.56  0.53  1.38  0.56  1.46  0.41  0.44  0.61  0.66  0.35  0.32  0.56  0.56  0.30  0.44  0.57  -0.31  0.46  0.59  -2.21  -0.69  0.54  0.71  0.85  0.59  0.40  0.39  0.33  0.41  0.46  0.86  0.55  0.63  0.60  0.61  0.53  0.94  0.74  0.50  0.51  1.56  1.50  1.25  0.58  0.83  0.42  0.51  1.71  0.47  -0.30  0.61  0.43  1.04  0.36  0.34  0.57  -0.51  0.79  -0.35  0.50  0.45  0.24  0.92  -0.46  0.60  0.46  0.57  0.39  0.51  0.43  0.70  0.41  0.67  0.95  2.39  0.67  -0.43  1.61  0.52  0.51  0.41  0.53  0.39  -0.31  0.88  -0.40  0.65  0.38  0.55  0.34  0.40  -0.61  1.21  0.31  -0.45  0.71  0.61  0.40  0.58  0.48  0.47  0.51  0.35  0.71  0.40  0.42  0.47  -0.32  0.32  -0.40  0.29  -0.42  -0.38  0.54  0.75  0.34  -0.39 | 5.62E-10  6.91E-09  1.03E-08  3.31E-08  3.73E-08  3.86E-08  5.88E-08  6.45E-08  6.60E-08  7.93E-08  8.06E-08  9.55E-08  9.93E-08  1.25E-07  1.37E-07  1.42E-07  1.66E-07  2.58E-07  2.59E-07  2.76E-07  2.85E-07  2.87E-07  3.03E-07  4.45E-07  5.65E-07  6.93E-07  7.14E-07  9.55E-07  1.09E-06  1.10E-06  1.33E-06  1.33E-06  1.58E-06  1.62E-06  1.65E-06  1.80E-06  1.81E-06  2.01E-06  2.57E-06  2.88E-06  2.98E-06  3.58E-06  4.00E-06  4.08E-06  4.18E-06  4.18E-06  4.21E-06  5.21E-06  5.35E-06  5.92E-06  5.95E-06  5.98E-06  6.29E-06  6.46E-06  6.65E-06  6.69E-06  6.71E-06  6.73E-06  6.74E-06  7.26E-06  7.78E-06  8.21E-06  8.79E-06  9.11E-06  9.14E-06  9.54E-06  9.69E-06  1.01E-05  1.05E-05  1.06E-05  1.13E-05  1.30E-05  1.30E-05  1.36E-05  1.38E-05  1.49E-05  1.57E-05  1.59E-05  1.61E-05  1.67E-05  1.68E-05  1.75E-05  1.81E-05  1.95E-05  2.01E-05  2.02E-05  2.17E-05  2.18E-05  2.27E-05  2.33E-05  2.34E-05  2.88E-05  2.95E-05  3.06E-05  3.21E-05  3.24E-05  3.64E-05  3.70E-05  3.76E-05  3.88E-05  3.90E-05  3.96E-05  4.13E-05  4.18E-05  4.28E-05  4.38E-05  4.49E-05  4.49E-05  4.58E-05  4.72E-05  4.75E-05  5.05E-05  5.14E-05  5.52E-05  5.55E-05  5.61E-05  5.66E-05  5.87E-05  5.98E-05  6.28E-05  6.96E-05  7.15E-05  7.29E-05  8.15E-05  8.16E-05  8.34E-05  9.60E-05  1.00E-04  1.02E-04  1.02E-04  1.03E-04  1.12E-04  1.20E-04  1.23E-04  1.24E-04  1.26E-04  1.28E-04  1.28E-04  1.28E-04  1.30E-04  1.31E-04  1.33E-04  1.40E-04  1.41E-04  1.43E-04  1.47E-04  1.47E-04  1.55E-04  1.68E-04  1.68E-04  1.75E-04  1.81E-04  1.96E-04  2.07E-04  2.07E-04  2.12E-04  2.22E-04  2.24E-04  2.27E-04  2.33E-04  2.34E-04  2.36E-04  2.37E-04  2.41E-04  2.43E-04  2.62E-04  2.66E-04  2.71E-04  2.71E-04  2.79E-04  2.84E-04  2.87E-04  2.95E-04  2.95E-04  3.14E-04  3.28E-04  3.37E-04  3.39E-04  3.41E-04  3.46E-04  3.47E-04  3.52E-04  3.53E-04  3.61E-04  3.67E-04  3.76E-04  3.92E-04  4.01E-04  4.05E-04  4.06E-04  4.11E-04  4.15E-04  4.18E-04  4.20E-04  4.32E-04  4.53E-04  4.56E-04  4.58E-04  4.64E-04  4.67E-04  4.70E-04  4.73E-04  4.79E-04  4.83E-04  4.90E-04  4.95E-04  4.97E-04  5.60E-04  5.61E-04  5.63E-04  5.87E-04  5.95E-04  5.96E-04  6.09E-04  6.14E-04  6.15E-04  6.29E-04  6.39E-04  6.40E-04  6.42E-04  6.44E-04  6.55E-04  6.55E-04  6.59E-04  6.87E-04  7.02E-04  7.14E-04  7.22E-04  7.29E-04  7.36E-04  7.44E-04  7.54E-04  7.98E-04  8.01E-04  8.14E-04  8.22E-04  8.22E-04  8.28E-04  8.39E-04  8.50E-04  8.58E-04  8.68E-04  8.91E-04  9.34E-04  9.48E-04  9.96E-04  1.01E-03  1.01E-03  1.01E-03  1.01E-03  1.02E-03  1.03E-03  1.08E-03  1.10E-03  1.10E-03  1.12E-03  1.12E-03  1.13E-03  1.13E-03  1.14E-03  1.14E-03  1.18E-03  1.18E-03  1.19E-03  1.19E-03  1.24E-03  1.25E-03  1.33E-03  1.33E-03  1.34E-03  1.36E-03  1.36E-03  1.37E-03  1.42E-03  1.43E-03  1.47E-03  1.48E-03  1.51E-03  1.54E-03  1.55E-03  1.56E-03  1.57E-03  1.62E-03  1.63E-03  1.66E-03  1.71E-03  1.72E-03  1.72E-03  1.73E-03  1.73E-03  1.73E-03  1.74E-03  1.76E-03  1.78E-03  1.79E-03  1.84E-03  1.85E-03  1.85E-03  1.88E-03  1.91E-03  1.96E-03  1.99E-03  1.99E-03 | 4.25E-06  2.60E-05  2.60E-05  4.86E-05  4.86E-05  4.86E-05  5.53E-05  5.53E-05  5.53E-05  5.53E-05  5.53E-05  5.77E-05  5.77E-05  6.70E-05  6.70E-05  6.70E-05  7.39E-05  9.85E-05  9.85E-05  9.85E-05  9.85E-05  9.85E-05  9.94E-05  1.40E-04  1.71E-04  2.00E-04  2.00E-04  2.58E-04  2.77E-04  2.77E-04  3.15E-04  3.15E-04  3.56E-04  3.56E-04  3.56E-04  3.69E-04  3.69E-04  3.99E-04  4.98E-04  5.44E-04  5.50E-04  6.44E-04  6.76E-04  6.76E-04  6.76E-04  6.76E-04  6.76E-04  8.19E-04  8.25E-04  8.63E-04  8.63E-04  8.63E-04  8.63E-04  8.63E-04  8.63E-04  8.63E-04  8.63E-04  8.63E-04  8.63E-04  9.14E-04  9.63E-04  1.00E-03  1.05E-03  1.06E-03  1.06E-03  1.09E-03  1.09E-03  1.13E-03  1.14E-03  1.14E-03  1.20E-03  1.35E-03  1.35E-03  1.39E-03  1.39E-03  1.48E-03  1.54E-03  1.54E-03  1.54E-03  1.57E-03  1.57E-03  1.61E-03  1.65E-03  1.75E-03  1.77E-03  1.77E-03  1.87E-03  1.87E-03  1.93E-03  1.94E-03  1.94E-03  2.37E-03  2.40E-03  2.46E-03  2.55E-03  2.55E-03  2.84E-03  2.85E-03  2.87E-03  2.91E-03  2.91E-03  2.93E-03  3.03E-03  3.04E-03  3.08E-03  3.12E-03  3.14E-03  3.14E-03  3.18E-03  3.23E-03  3.23E-03  3.41E-03  3.44E-03  3.64E-03  3.64E-03  3.65E-03  3.65E-03  3.76E-03  3.80E-03  3.95E-03  4.35E-03  4.42E-03  4.48E-03  4.93E-03  4.93E-03  5.00E-03  5.71E-03  5.90E-03  5.92E-03  5.92E-03  5.93E-03  6.42E-03  6.79E-03  6.95E-03  6.96E-03  6.98E-03  6.98E-03  6.98E-03  6.98E-03  7.00E-03  7.04E-03  7.08E-03  7.40E-03  7.40E-03  7.44E-03  7.55E-03  7.55E-03  7.90E-03  8.45E-03  8.45E-03  8.77E-03  9.00E-03  9.68E-03  1.01E-02  1.01E-02  1.03E-02  1.07E-02  1.07E-02  1.08E-02  1.10E-02  1.10E-02  1.10E-02  1.10E-02  1.11E-02  1.11E-02  1.19E-02  1.20E-02  1.21E-02  1.21E-02  1.24E-02  1.26E-02  1.26E-02  1.28E-02  1.28E-02  1.35E-02  1.41E-02  1.44E-02  1.44E-02  1.44E-02  1.45E-02  1.45E-02  1.46E-02  1.46E-02  1.48E-02  1.50E-02  1.53E-02  1.58E-02  1.61E-02  1.61E-02  1.61E-02  1.63E-02  1.63E-02  1.63E-02  1.64E-02  1.67E-02  1.75E-02  1.75E-02  1.75E-02  1.76E-02  1.76E-02  1.77E-02  1.77E-02  1.78E-02  1.79E-02  1.81E-02  1.81E-02  1.81E-02  2.02E-02  2.02E-02  2.02E-02  2.10E-02  2.11E-02  2.11E-02  2.15E-02  2.15E-02  2.15E-02  2.19E-02  2.20E-02  2.20E-02  2.20E-02  2.20E-02  2.22E-02  2.22E-02  2.22E-02  2.31E-02  2.35E-02  2.38E-02  2.39E-02  2.40E-02  2.42E-02  2.43E-02  2.45E-02  2.59E-02  2.59E-02  2.61E-02  2.62E-02  2.62E-02  2.63E-02  2.65E-02  2.67E-02  2.69E-02  2.71E-02  2.77E-02  2.89E-02  2.92E-02  3.06E-02  3.06E-02  3.06E-02  3.06E-02  3.06E-02  3.06E-02  3.10E-02  3.23E-02  3.26E-02  3.26E-02  3.29E-02  3.30E-02  3.30E-02  3.30E-02  3.31E-02  3.31E-02  3.38E-02  3.38E-02  3.38E-02  3.38E-02  3.53E-02  3.54E-02  3.73E-02  3.73E-02  3.75E-02  3.77E-02  3.77E-02  3.79E-02  3.91E-02  3.92E-02  4.01E-02  4.04E-02  4.09E-02  4.17E-02  4.18E-02  4.18E-02  4.21E-02  4.33E-02  4.33E-02  4.40E-02  4.50E-02  4.50E-02  4.50E-02  4.50E-02  4.50E-02  4.50E-02  4.51E-02  4.53E-02  4.58E-02  4.58E-02  4.70E-02  4.70E-02  4.70E-02  4.74E-02  4.82E-02  4.93E-02  4.96E-02  4.96E-02 |
| **Hippocampus** | | | | | | |
| **Gene name** | **Ensembl ID** | | **Fold change** | **Log2 fold change** | **pval** | **adj.pval** |
| Malat1  Tia1  Gm37376  Ablim1  Vstm2l  Rps2  Ndufs7  Ndufa11  Gm16586  Ubp1  Ddx17  Nr2f6  Ccar1  4930402H24Rik  Supt20  Dgcr6  Map1lc3a  Uqcr10  Tbr1  Ifitm10  Psd  Znhit2  Thra  Mir1906-1  Rprml  Tpgs1  Cox8a  Efr3a  Foxo6  Uqcrc1  Prdx5  Rpl13  Mir124a-1hg | ENSMUSG00000092341  ENSMUSG00000071337  ENSMUSG00000102349  ENSMUSG00000025085  ENSMUSG00000037843  ENSMUSG00000044533  ENSMUSG00000020153  ENSMUSG00000002379  ENSMUSG00000089788  ENSMUSG00000009741  ENSMUSG00000055065  ENSMUSG00000002393  ENSMUSG00000020074  ENSMUSG00000027309  ENSMUSG00000027751  ENSMUSG00000003531  ENSMUSG00000027602  ENSMUSG00000059534  ENSMUSG00000035033  ENSMUSG00000045777  ENSMUSG00000037126  ENSMUSG00000075227  ENSMUSG00000058756  ENSMUSG00000084535  ENSMUSG00000046215  ENSMUSG00000020308  ENSMUSG00000035885  ENSMUSG00000015002  ENSMUSG00000052135  ENSMUSG00000025651  ENSMUSG00000024953  ENSMUSG00000000740  ENSMUSG00000097545 | | 2.81  1.67  2.64  1.50  -1.61  -1.58  -1.43  -1.43  1.64  1.57  1.55  -1.77  1.47  1.38  1.37  -1.44  -1.38  -1.40  1.51  -1.41  -1.37  -1.60  -1.29  2.33  -1.54  -1.54  -1.35  1.37  -1.44  -1.32  -1.35  -1.31  2.16 | 1.49  0.74  1.40  0.58  -0.69  -0.66  -0.51  -0.52  0.71  0.65  0.63  -0.82  0.56  0.47  0.45  -0.53  -0.47  -0.49  0.59  -0.49  -0.45  -0.68  -0.37  1.22  -0.62  -0.62  -0.44  0.45  -0.52  -0.40  -0.43  -0.39  1.11 | 1.11E-07  3.76E-06  1.13E-05  1.74E-05  2.18E-05  2.21E-05  6.01E-05  6.47E-05  6.60E-05  7.91E-05  9.02E-05  9.78E-05  1.01E-04  1.04E-04  1.05E-04  1.06E-04  1.06E-04  1.11E-04  1.19E-04  1.23E-04  1.26E-04  1.34E-04  1.35E-04  1.45E-04  1.45E-04  1.45E-04  1.60E-04  1.82E-04  1.90E-04  1.94E-04  2.10E-04  2.11E-04  2.23E-04 | 7.77E-04  1.32E-02  2.59E-02  2.59E-02  2.59E-02  2.59E-02  3.92E-02  3.92E-02  3.92E-02  3.92E-02  3.92E-02  3.92E-02  3.92E-02  3.92E-02  3.92E-02  3.92E-02  3.92E-02  3.92E-02  3.92E-02  3.92E-02  3.92E-02  3.92E-02  3.92E-02  3.92E-02  3.92E-02  3.92E-02  4.17E-02  4.53E-02  4.53E-02  4.53E-02  4.63E-02  4.63E-02  4.75E-02 |
| **Ventral Striatum** | | | | | | |
| **Gene name** | **Ensembl ID** | | **Fold change** | **Log2 fold change** | **pval** | **adj.pval** |
| Neto2  Kctd12  Grm5  Pcmtd2  Spock3  Necab1  Gpm6b  Erbb2ip  Secisbp2l  Mef2a  Cttnbp2 | ENSMUSG00000036902  ENSMUSG00000098557  ENSMUSG00000049583  ENSMUSG00000027589  ENSMUSG00000054162  ENSMUSG00000040536  ENSMUSG00000031342  ENSMUSG00000021709  ENSMUSG00000035093  ENSMUSG00000030557  ENSMUSG00000000416 | | 2.00  1.90  2.11  1.82  2.55  1.79  1.76  1.68  1.59  1.66  1.95 | 1.00  0.93  1.07  0.87  1.35  0.84  0.82  0.75  0.67  0.73  0.96 | 4.69E-07  3.07E-06  3.92E-06  6.38E-06  1.56E-05  2.02E-05  2.33E-05  7.58E-05  8.65E-05  9.28E-05  1.00E-04 | 4.69E-07  3.07E-06  3.92E-06  6.38E-06  1.56E-05  2.02E-05  2.33E-05  7.58E-05  8.65E-05  9.28E-05  1.00E-04 |

**(B)** Differentially expressed genes between male Tg and NTg groups

| **Amygdala** | | | | | | |
| --- | --- | --- | --- | --- | --- | --- |
| **Gene name** | **Ensembl ID** | | **Fold change** | **Log2 fold change** | **pval** | **adj.pval** |
| Ttr  Calml4  Lbp  1500015O10Rik  Gm26917  Kcne2  Nnat  Snhg3 | ENSMUSG00000061808  ENSMUSG00000032246  ENSMUSG00000016024  ENSMUSG00000026051  ENSMUSG00000097971  ENSMUSG00000039672  ENSMUSG00000067786  ENSMUSG00000085241 | | 201.02  13.76  7.49  20.56  3.49  43.18  2.12  2.11 | 7.65  3.78  2.91  4.36  1.80  5.43  1.08  1.08 | 5.68E-11  2.88E-07  4.50E-07  7.09E-07  1.67E-06  2.43E-06  1.45E-05  4.83E-05 | 4.37E-07  1.11E-03  1.15E-03  1.36E-03  2.56E-03  3.12E-03  1.59E-02  4.65E-02 |
| **Hypothalamus** | | | | | | |
| **Gene name** | | **Ensembl ID** | **Fold change** | **Log2 fold change** | **pval** | **adj.pval** |
| Kmt2b  Agrn  Zfr2  Unc13a  Gcn1l1  Sema6c  Bzrap1  Celsr3  Gpc1  Dync1h1  Plxnb1  Map4k2  Epha10  Gm26377  Eid1  Gm16740  Sptbn4  Slc4a3  Zswim8  Adam11  Col16a1  Crocc  Plec  Plxna3  Setd1a  Gm996  Gm26702  Dot1l  Ptpru  Zfp512b  Vars  Wnk2  Mroh1  Cyth1  Speg  Ints1  Col11a2  Col4a2  Lrrc16b  Pcnxl3  Fcho1  Atp8b2  Pnrc2  PISD  Myh9  Slc9a5  Gigyf1  Atg2a  Phc1  Dgkz  Scrib  P3h3  Cacna1g  Tmem35  Cacna1h  Chd5  Gtf3c1  Lrp1  Pkd1  Flna  Lmtk3  Rgp1  Ptprs  Edc4  Ywhaq  Tmem191c  Sptbn2  Trrap  B4galnt4  Myo9b  Megf8  Rgs4  Vwa5b2  Sema4g  Magel2  Zfp598  Ppfia4  Arhgap33  Cetn2  Nckap5l  RP23-325D10.3  5031439G07Rik  Dek  Gda  Eif1a  Rab2a  Vps9d1  Calb1  Slc16a11  Cbx7  Inpp5j  Col4a1  Plekhg5  Fasn  Usp19  Plxnb2  Impa1  Nptx2  Mbd6  Ltbp4  Ubr4  Cnbp  Prpf40b  Colgalt1  Sep-15  Inf2  Slc29a2  Inha  Sar1b  Rab11a  Gkap1  Tac1  Zfp384  Rab18  Ptpn23  Rap1gap  Dstn  Phldb1  Ppp2ca  Adgrb1  Cmpk1  Grik5  Fbxl19  Cxcl14  Mast1  Zgpat  Dvl1  Mllt11  Kcnh2  Madd  Ankrd24  Ptges3  Clcn2  Ube2r2  Ube2e1  Srrm2  BC037034  6330403K07Rik  Kcnt1  Pid1  Ap1s2  Ube2o  Ercc2  Ssr3  Srrm1  Lmo3  Lmo4  Prrc2a  Abca2  Dennd6b  Rdh13  Fam193b  Ano8  Pdgfa  Msantd4  Man2c1  Nktr  Cfl2  Atp13a2  Hdac7  Jag2  Dopey2  Chn1  Snapc4  Zfp523  Fkbp1a  Rap1b  Fbrs  Tceal1  Rhoa  Gon4l  Arhgef1  Cabin1  B3gat1  Lzts3  Morf4l1  Repin1  Spred1  Spryd7  Serpina3n  Psd  Mfsd10  Pcp4  Eif3e  Tra2b  Rnpepl1  Sptb  Tuba1a  Arpc5  Apc2  Kif21b  Dst  Nr1h2  Tbkbp1  Ank1  Itm2b  Thbs3  Erlec1  Tmed7  Lrp3  Mcts1 | | ENSMUSG00000006307  ENSMUSG00000041936  ENSMUSG00000034949  ENSMUSG00000034799  ENSMUSG00000041638  ENSMUSG00000038777  ENSMUSG00000034156  ENSMUSG00000023473  ENSMUSG00000034220  ENSMUSG00000018707  ENSMUSG00000053646  ENSMUSG00000024948  ENSMUSG00000028876  ENSMUSG00000076138  ENSMUSG00000091337  ENSMUSG00000097167  ENSMUSG00000011751  ENSMUSG00000006576  ENSMUSG00000021819  ENSMUSG00000020926  ENSMUSG00000040690  ENSMUSG00000040860  ENSMUSG00000022565  ENSMUSG00000031398  ENSMUSG00000042308  ENSMUSG00000029419  ENSMUSG00000097872  ENSMUSG00000061589  ENSMUSG00000028909  ENSMUSG00000000823  ENSMUSG00000007029  ENSMUSG00000037989  ENSMUSG00000022558  ENSMUSG00000017132  ENSMUSG00000026207  ENSMUSG00000029547  ENSMUSG00000024330  ENSMUSG00000031503  ENSMUSG00000022211  ENSMUSG00000054874  ENSMUSG00000070000  ENSMUSG00000060671  ENSMUSG00000028675  ENSMUSG00000095041  ENSMUSG00000022443  ENSMUSG00000014786  ENSMUSG00000029714  ENSMUSG00000024773  ENSMUSG00000040669  ENSMUSG00000040479  ENSMUSG00000022568  ENSMUSG00000023191  ENSMUSG00000020866  ENSMUSG00000033578  ENSMUSG00000024112  ENSMUSG00000005045  ENSMUSG00000032777  ENSMUSG00000040249  ENSMUSG00000032855  ENSMUSG00000031328  ENSMUSG00000062044  ENSMUSG00000028468  ENSMUSG00000013236  ENSMUSG00000036270  ENSMUSG00000076432  ENSMUSG00000055692  ENSMUSG00000067889  ENSMUSG00000045482  ENSMUSG00000055629  ENSMUSG00000004677  ENSMUSG00000045039  ENSMUSG00000038530  ENSMUSG00000046613  ENSMUSG00000025207  ENSMUSG00000056972  ENSMUSG00000041130  ENSMUSG00000026458  ENSMUSG00000036882  ENSMUSG00000031347  ENSMUSG00000023009  ENSMUSG00000108815  ENSMUSG00000036046  ENSMUSG00000021377  ENSMUSG00000058624  ENSMUSG00000057561  ENSMUSG00000047187  ENSMUSG00000001062  ENSMUSG00000028222  ENSMUSG00000040938  ENSMUSG00000053411  ENSMUSG00000034570  ENSMUSG00000031502  ENSMUSG00000039713  ENSMUSG00000025153  ENSMUSG00000006676  ENSMUSG00000036606  ENSMUSG00000027531  ENSMUSG00000059991  ENSMUSG00000025409  ENSMUSG00000040488  ENSMUSG00000066036  ENSMUSG00000030057  ENSMUSG00000023007  ENSMUSG00000034807  ENSMUSG00000037072  ENSMUSG00000037679  ENSMUSG00000024891  ENSMUSG00000032968  ENSMUSG00000020386  ENSMUSG00000004771  ENSMUSG00000021552  ENSMUSG00000061762  ENSMUSG00000038346  ENSMUSG00000073639  ENSMUSG00000036057  ENSMUSG00000041351  ENSMUSG00000015932  ENSMUSG00000048537  ENSMUSG00000020349  ENSMUSG00000034730  ENSMUSG00000028719  ENSMUSG00000003378  ENSMUSG00000030811  ENSMUSG00000021508  ENSMUSG00000053693  ENSMUSG00000027582  ENSMUSG00000029071  ENSMUSG00000053192  ENSMUSG00000038319  ENSMUSG00000040687  ENSMUSG00000054708  ENSMUSG00000071072  ENSMUSG00000022843  ENSMUSG00000036241  ENSMUSG00000021774  ENSMUSG00000039218  ENSMUSG00000036948  ENSMUSG00000018451  ENSMUSG00000058740  ENSMUSG00000045658  ENSMUSG00000031367  ENSMUSG00000020802  ENSMUSG00000030400  ENSMUSG00000027828  ENSMUSG00000028809  ENSMUSG00000030226  ENSMUSG00000028266  ENSMUSG00000024393  ENSMUSG00000026944  ENSMUSG00000015377  ENSMUSG00000008435  ENSMUSG00000021495  ENSMUSG00000034863  ENSMUSG00000025856  ENSMUSG00000041124  ENSMUSG00000032295  ENSMUSG00000032525  ENSMUSG00000062929  ENSMUSG00000036622  ENSMUSG00000022475  ENSMUSG00000002799  ENSMUSG00000022946  ENSMUSG00000056486  ENSMUSG00000036281  ENSMUSG00000024220  ENSMUSG00000032966  ENSMUSG00000052681  ENSMUSG00000042423  ENSMUSG00000049536  ENSMUSG00000007815  ENSMUSG00000054199  ENSMUSG00000040940  ENSMUSG00000020196  ENSMUSG00000045994  ENSMUSG00000037703  ENSMUSG00000062270  ENSMUSG00000052751  ENSMUSG00000027351  ENSMUSG00000021930  ENSMUSG00000021091  ENSMUSG00000037126  ENSMUSG00000001082  ENSMUSG00000090223  ENSMUSG00000022336  ENSMUSG00000022858  ENSMUSG00000026269  ENSMUSG00000021061  ENSMUSG00000072235  ENSMUSG00000008475  ENSMUSG00000020135  ENSMUSG00000041642  ENSMUSG00000026131  ENSMUSG00000060601  ENSMUSG00000038517  ENSMUSG00000031543  ENSMUSG00000022108  ENSMUSG00000028047  ENSMUSG00000020311  ENSMUSG00000033184  ENSMUSG00000001802  ENSMUSG00000000355 | 1.62  1.55  1.44  1.48  1.48  1.72  1.74  1.69  1.65  1.35  1.34  1.66  1.72  -1.93  -1.50  -1.55  1.56  1.43  1.41  1.42  2.12  1.69  1.49  1.48  1.42  1.37  1.37  1.63  1.52  1.50  1.46  1.41  1.38  1.44  1.49  1.44  1.93  1.65  1.56  1.54  1.49  1.31  -1.43  2.00  1.46  1.65  1.62  1.50  1.29  1.53  1.58  1.88  1.61  -1.34  1.39  1.29  1.30  1.39  1.49  1.61  1.41  1.33  1.30  1.29  -1.36  1.39  1.24  1.36  1.30  1.62  1.21  -1.33  1.55  1.56  1.34  1.46  1.29  1.47  -1.48  1.43  1.41  1.43  -1.46  -1.46  -1.38  -1.31  1.39  -1.35  1.87  1.47  1.43  1.54  1.44  1.28  1.29  1.30  -1.37  -1.38  1.76  1.44  1.36  -1.28  1.51  1.32  -1.34  1.55  1.46  1.78  -1.41  -1.47  -1.45  -1.45  1.45  -1.65  1.45  1.24  -1.34  1.44  -1.31  1.29  -1.44  1.25  1.33  -1.25  1.33  1.51  1.46  -1.31  1.29  1.20  1.57  -1.59  1.48  -1.32  -1.38  1.50  1.34  -1.24  1.44  -1.57  -1.54  1.23  1.59  -1.28  1.31  -1.47  -1.43  1.29  1.29  1.53  1.37  1.71  1.49  -1.32  -1.33  1.42  1.45  -1.44  1.33  1.68  1.38  1.31  -1.22  1.60  1.51  -1.27  -1.55  1.33  -1.34  -1.29  1.34  1.57  1.27  1.21  1.24  -1.25  1.31  -1.78  -1.37  -1.28  1.29  1.54  -1.31  -1.41  -1.39  1.43  1.27  -1.21  -1.30  1.29  1.25  1.35  1.38  1.50  1.35  -1.20  1.52  -1.32  -1.96  1.21  -1.60 | 0.69  0.63  0.53  0.56  0.56  0.78  0.80  0.76  0.72  0.43  0.42  0.73  0.78  -0.95  -0.59  -0.63  0.64  0.52  0.49  0.50  1.08  0.76  0.57  0.57  0.51  0.46  0.45  0.70  0.61  0.58  0.55  0.49  0.46  0.53  0.57  0.53  0.95  0.72  0.64  0.63  0.57  0.39  -0.51  1.00  0.55  0.72  0.69  0.59  0.37  0.61  0.66  0.91  0.69  -0.43  0.47  0.37  0.38  0.47  0.58  0.68  0.49  0.41  0.38  0.36  -0.44  0.48  0.31  0.45  0.38  0.70  0.28  -0.41  0.64  0.64  0.42  0.55  0.37  0.55  -0.56  0.52  0.49  0.51  -0.55  -0.54  -0.47  -0.39  0.48  -0.43  0.90  0.56  0.52  0.62  0.53  0.36  0.37  0.38  -0.46  -0.46  0.82  0.53  0.45  -0.36  0.60  0.40  -0.43  0.63  0.54  0.83  -0.49  -0.56  -0.53  -0.53  0.54  -0.72  0.53  0.31  -0.42  0.52  -0.39  0.36  -0.52  0.32  0.41  -0.32  0.41  0.59  0.54  -0.39  0.37  0.27  0.65  -0.67  0.56  -0.40  -0.47  0.58  0.42  -0.31  0.53  -0.65  -0.63  0.30  0.67  -0.36  0.39  -0.56  -0.52  0.37  0.37  0.61  0.45  0.77  0.58  -0.40  -0.41  0.51  0.54  -0.53  0.41  0.74  0.47  0.39  -0.29  0.68  0.59  -0.34  -0.63  0.41  -0.42  -0.37  0.42  0.65  0.35  0.28  0.31  -0.32  0.38  -0.83  -0.46  -0.35  0.37  0.63  -0.39  -0.50  -0.48  0.51  0.34  -0.28  -0.38  0.37  0.32  0.43  0.46  0.58  0.43  -0.26  0.60  -0.40  -0.97  0.28  -0.68 | 1.15E-11  4.94E-08  3.62E-08  1.08E-07  1.51E-07  2.38E-07  5.88E-07  6.22E-07  4.71E-07  5.80E-07  4.00E-07  1.31E-06  1.76E-06  1.71E-06  2.39E-06  2.36E-06  2.67E-06  3.33E-06  3.23E-06  3.56E-06  9.05E-06  7.97E-06  8.18E-06  8.71E-06  9.14E-06  8.37E-06  1.03E-05  1.08E-05  1.11E-05  1.38E-05  1.38E-05  1.31E-05  1.46E-05  1.58E-05  1.67E-05  2.07E-05  2.44E-05  2.52E-05  2.42E-05  2.45E-05  2.60E-05  2.31E-05  2.62E-05  2.84E-05  3.25E-05  3.48E-05  3.54E-05  3.48E-05  3.61E-05  3.89E-05  4.07E-05  4.64E-05  4.62E-05  4.64E-05  5.86E-05  6.13E-05  6.51E-05  6.89E-05  7.09E-05  7.49E-05  8.01E-05  8.07E-05  7.92E-05  8.23E-05  8.36E-05  8.50E-05  9.71E-05  1.02E-04  1.03E-04  1.05E-04  1.08E-04  1.07E-04  1.11E-04  1.14E-04  1.17E-04  1.17E-04  1.22E-04  1.23E-04  1.30E-04  1.38E-04  1.40E-04  1.42E-04  1.43E-04  1.51E-04  1.53E-04  1.55E-04  1.62E-04  1.63E-04  1.71E-04  1.75E-04  1.77E-04  1.81E-04  1.85E-04  1.93E-04  2.04E-04  2.16E-04  2.17E-04  2.22E-04  2.41E-04  2.45E-04  2.53E-04  2.59E-04  2.62E-04  2.78E-04  2.96E-04  2.97E-04  2.99E-04  3.02E-04  3.08E-04  3.08E-04  3.17E-04  3.42E-04  3.59E-04  3.89E-04  3.95E-04  3.97E-04  4.01E-04  4.05E-04  4.10E-04  4.27E-04  4.33E-04  4.40E-04  4.50E-04  4.64E-04  4.72E-04  4.78E-04  4.96E-04  5.51E-04  5.52E-04  5.55E-04  5.58E-04  5.70E-04  5.78E-04  6.08E-04  6.21E-04  6.26E-04  6.52E-04  6.59E-04  6.84E-04  7.15E-04  7.18E-04  7.34E-04  7.77E-04  7.90E-04  7.92E-04  8.16E-04  8.26E-04  8.32E-04  8.51E-04  8.55E-04  8.57E-04  8.70E-04  8.73E-04  8.85E-04  9.04E-04  9.33E-04  9.36E-04  9.47E-04  9.55E-04  9.59E-04  9.82E-04  1.02E-03  1.03E-03  1.04E-03  1.04E-03  1.05E-03  1.05E-03  1.08E-03  1.08E-03  1.10E-03  1.11E-03  1.11E-03  1.11E-03  1.11E-03  1.14E-03  1.15E-03  1.16E-03  1.17E-03  1.18E-03  1.19E-03  1.21E-03  1.26E-03  1.30E-03  1.31E-03  1.33E-03  1.35E-03  1.36E-03  1.37E-03  1.37E-03  1.39E-03  1.39E-03  1.40E-03  1.41E-03  1.42E-03  1.42E-03  1.43E-03  1.44E-03  1.45E-03  1.48E-03  1.50E-03  1.52E-03 | 7.52E-08  1.08E-04  1.08E-04  1.77E-04  1.97E-04  2.59E-04  3.69E-04  3.69E-04  3.69E-04  3.69E-04  3.69E-04  7.12E-04  8.22E-04  8.22E-04  9.75E-04  9.75E-04  1.02E-03  1.14E-03  1.14E-03  1.16E-03  2.29E-03  2.29E-03  2.29E-03  2.29E-03  2.29E-03  2.29E-03  2.49E-03  2.49E-03  2.49E-03  2.82E-03  2.82E-03  2.82E-03  2.89E-03  3.03E-03  3.12E-03  3.76E-03  3.97E-03  3.97E-03  3.97E-03  3.97E-03  3.97E-03  3.97E-03  3.97E-03  4.22E-03  4.72E-03  4.81E-03  4.81E-03  4.81E-03  4.81E-03  5.08E-03  5.20E-03  5.61E-03  5.61E-03  5.61E-03  6.96E-03  7.15E-03  7.45E-03  7.76E-03  7.84E-03  8.15E-03  8.36E-03  8.36E-03  8.36E-03  8.39E-03  8.39E-03  8.40E-03  9.46E-03  9.73E-03  9.73E-03  9.81E-03  9.81E-03  9.81E-03  9.89E-03  1.00E-02  1.00E-02  1.00E-02  1.03E-02  1.03E-02  1.08E-02  1.12E-02  1.13E-02  1.13E-02  1.13E-02  1.18E-02  1.18E-02  1.18E-02  1.21E-02  1.21E-02  1.25E-02  1.27E-02  1.27E-02  1.29E-02  1.30E-02  1.34E-02  1.40E-02  1.46E-02  1.46E-02  1.48E-02  1.59E-02  1.60E-02  1.63E-02  1.66E-02  1.66E-02  1.74E-02  1.83E-02  1.83E-02  1.83E-02  1.83E-02  1.83E-02  1.83E-02  1.86E-02  1.99E-02  2.07E-02  2.23E-02  2.23E-02  2.23E-02  2.24E-02  2.24E-02  2.25E-02  2.32E-02  2.34E-02  2.36E-02  2.39E-02  2.44E-02  2.46E-02  2.48E-02  2.55E-02  2.78E-02  2.78E-02  2.78E-02  2.78E-02  2.82E-02  2.84E-02  2.96E-02  3.00E-02  3.01E-02  3.11E-02  3.12E-02  3.21E-02  3.32E-02  3.32E-02  3.37E-02  3.54E-02  3.56E-02  3.56E-02  3.65E-02  3.67E-02  3.67E-02  3.71E-02  3.71E-02  3.71E-02  3.72E-02  3.72E-02  3.75E-02  3.81E-02  3.89E-02  3.89E-02  3.91E-02  3.91E-02  3.91E-02  3.98E-02  4.11E-02  4.11E-02  4.12E-02  4.12E-02  4.12E-02  4.12E-02  4.18E-02  4.18E-02  4.18E-02  4.18E-02  4.18E-02  4.18E-02  4.18E-02  4.24E-02  4.27E-02  4.28E-02  4.29E-02  4.29E-02  4.31E-02  4.36E-02  4.52E-02  4.65E-02  4.65E-02  4.70E-02  4.74E-02  4.74E-02  4.74E-02  4.74E-02  4.75E-02  4.75E-02  4.76E-02  4.76E-02  4.76E-02  4.76E-02  4.78E-02  4.78E-02  4.78E-02  4.85E-02  4.90E-02  4.95E-02 |
| **Hippocampus** | | | | | | |
| **Gene name** | **Ensembl ID** | | **Fold change** | **Log2 fold change** | **pval** | **adj.pval** |
| n/a | n/a | | n/a | n/a | n/a | n/a |
| **Ventral Striatum** | | | | | | |
| **Gene name** | **Ensembl ID** | | **Fold change** | **Log2 fold change** | **pval** | **adj.pval** |
| n/a | n/a | | n/a | n/a | n/a | n/a |

**(C)** Differentially expressed genes between male NTg and WT groups

| **Amygdala** | | | | | | |
| --- | --- | --- | --- | --- | --- | --- |
| **Gene name** | **Ensembl ID** | | **Fold change** | **Log2 fold change** | **pval** | **adj.pval** |
| n/a | n/a | | n/a | n/a | n/a | n/a |
| **Hypothalamus** | | | | | | |
| **Gene name** | | **Ensembl ID** | **Fold change** | **Log2 fold change** | **pval** | **adj.pval** |
| n/a | | n/a | n/a | n/a | n/a | n/a |
| **Hippocampus** | | | | | | |
| **Gene name** | **Ensembl ID** | | **Fold change** | **Log2 fold change** | **pval** | **adj.pval** |
| Pde4b  Rpl13  Ptprd  Cox8a  Cdh2  Tceb2  Ccar1  Rprml  Sgsm1  Psd  Ank2  Uqcr10  Usp54  AW047730  Map4k3  Cenpb  Tbr1  Fam131a  Specc1  Rps15  Cst3  Atxn2  Clcn6  Cyfip2  Map1b  Lmo1  Dgcr6  Vstm2l  Cox4i1  Tnr  Fmn2  Ablim1  Zc3h11a  Clip3  Ndufa2  Tmem160  Ifitm10  Stox2  Bloc1s1  Limch1  Map1lc3a  Rab5a  Tjp1  4930402H24Rik  Fxyd2  Cox6a1  Luc7l2  Ramp1  Ank3  Rps5  Selm  Efr3a  Rps14  Ndufa11  Znhit2  Nemf  Wnk1  Gm16755  Prkar2a  Cep120  Son  Bicd2  Mrps7  Serinc5  Uqcrq  Setd7  Med14  Cnot4  Polr3h  Mt2  1110065P20Rik  Nampt  Ssbp4  Dst  Neo1  Scand1  Lin7b  Ndufs7  Cox5b  Dbp  Gm20441  Gm44330  Frmd4a  Dtna  Gm15954  Mgat5  Tox4  Sgip1  Itsn1  Prdx5  Rbm25  Dhx9  Mtag2  Mrpl57  B4galt2  Ubap2l  Rps3a1  Ppm1l  Rps12  Slc22a23  Ppp1r1a  Ndufb9  Slc6a6  Mir5121  Ryr2  Adam9  Kif21a  Atp6v1f  Katnal1  Polr2a  Bmyc  Ndufb6  Prrc2c  Rnf187  Phlpp1  Mrps24  Mrpl11  Gm22133  Gnb2l1  Fam213b  Rpl14  Rpl8  Malat1  Dtnbp1  C1qtnf4  Grcc10 | ENSMUSG00000028525  ENSMUSG00000000740  ENSMUSG00000028399  ENSMUSG00000035885  ENSMUSG00000024304  ENSMUSG00000055839  ENSMUSG00000020074  ENSMUSG00000046215  ENSMUSG00000042216  ENSMUSG00000037126  ENSMUSG00000032826  ENSMUSG00000059534  ENSMUSG00000034235  ENSMUSG00000097428  ENSMUSG00000024242  ENSMUSG00000068267  ENSMUSG00000035033  ENSMUSG00000050821  ENSMUSG00000042331  ENSMUSG00000063457  ENSMUSG00000027447  ENSMUSG00000042605  ENSMUSG00000029016  ENSMUSG00000020340  ENSMUSG00000052727  ENSMUSG00000036111  ENSMUSG00000003531  ENSMUSG00000037843  ENSMUSG00000031818  ENSMUSG00000015829  ENSMUSG00000028354  ENSMUSG00000025085  ENSMUSG00000102976  ENSMUSG00000013921  ENSMUSG00000014294  ENSMUSG00000019158  ENSMUSG00000045777  ENSMUSG00000038143  ENSMUSG00000090247  ENSMUSG00000037736  ENSMUSG00000027602  ENSMUSG00000017831  ENSMUSG00000030516  ENSMUSG00000027309  ENSMUSG00000059412  ENSMUSG00000041697  ENSMUSG00000029823  ENSMUSG00000034353  ENSMUSG00000069601  ENSMUSG00000012848  ENSMUSG00000075702  ENSMUSG00000015002  ENSMUSG00000024608  ENSMUSG00000002379  ENSMUSG00000075227  ENSMUSG00000020982  ENSMUSG00000045962  ENSMUSG00000097658  ENSMUSG00000032601  ENSMUSG00000048799  ENSMUSG00000022961  ENSMUSG00000037933  ENSMUSG00000046756  ENSMUSG00000021703  ENSMUSG00000044894  ENSMUSG00000037111  ENSMUSG00000064127  ENSMUSG00000038784  ENSMUSG00000022476  ENSMUSG00000031762  ENSMUSG00000078570  ENSMUSG00000020572  ENSMUSG00000070003  ENSMUSG00000026131  ENSMUSG00000032340  ENSMUSG00000046229  ENSMUSG00000003872  ENSMUSG00000020153  ENSMUSG00000061518  ENSMUSG00000059824  ENSMUSG00000092360  ENSMUSG00000105878  ENSMUSG00000026657  ENSMUSG00000024302  ENSMUSG00000089979  ENSMUSG00000036155  ENSMUSG00000016831  ENSMUSG00000028524  ENSMUSG00000022957  ENSMUSG00000024953  ENSMUSG00000010608  ENSMUSG00000042699  ENSMUSG00000091510  ENSMUSG00000021967  ENSMUSG00000028541  ENSMUSG00000042520  ENSMUSG00000028081  ENSMUSG00000027784  ENSMUSG00000061983  ENSMUSG00000038267  ENSMUSG00000022490  ENSMUSG00000022354  ENSMUSG00000030096  ENSMUSG00000105913  ENSMUSG00000021313  ENSMUSG00000031555  ENSMUSG00000022629  ENSMUSG00000004285  ENSMUSG00000041298  ENSMUSG00000005198  ENSMUSG00000049086  ENSMUSG00000071014  ENSMUSG00000040225  ENSMUSG00000020496  ENSMUSG00000044340  ENSMUSG00000020477  ENSMUSG00000024902  ENSMUSG00000076036  ENSMUSG00000020372  ENSMUSG00000029059  ENSMUSG00000025794  ENSMUSG00000003970  ENSMUSG00000092341  ENSMUSG00000057531  ENSMUSG00000040794  ENSMUSG00000072772 | | 1.52  -1.53  1.61  -1.50  1.59  -1.48  1.62  -1.74  1.46  -1.47  1.52  -1.49  1.46  -1.50  1.45  -1.28  1.58  -1.27  1.38  -1.47  -1.33  1.33  1.31  1.31  1.44  -1.53  -1.47  -1.58  -1.39  1.53  1.49  1.46  1.66  1.26  -1.49  -1.59  -1.43  1.37  -1.44  1.46  -1.38  1.32  1.95  1.38  -1.47  -1.34  1.43  -1.37  1.43  -1.34  -1.59  1.37  -1.51  -1.40  -1.58  1.39  1.45  -1.53  1.40  1.30  1.46  1.28  -1.36  1.50  -1.48  1.53  1.31  1.30  -1.39  -1.59  -1.42  1.39  -1.55  1.44  1.34  -2.03  -1.61  -1.37  -1.45  -1.54  -1.65  2.89  1.49  1.41  -1.79  1.37  1.25  1.44  1.32  -1.33  1.36  1.45  -1.73  -1.41  -1.27  1.30  -1.31  1.32  -1.36  1.36  -1.55  -1.28  1.36  -1.66  1.63  1.32  1.61  -1.36  1.25  1.34  -1.38  -1.37  1.82  -1.25  1.29  -1.43  -1.38  -1.54  -1.27  -1.40  -1.31  -1.43  1.91  -1.34  -1.72  -1.41 | 0.60  -0.62  0.69  -0.59  0.67  -0.56  0.70  -0.80  0.54  -0.55  0.60  -0.58  0.54  -0.58  0.54  -0.35  0.66  -0.35  0.47  -0.56  -0.41  0.41  0.39  0.40  0.53  -0.61  -0.55  -0.66  -0.47  0.61  0.58  0.55  0.73  0.33  -0.57  -0.67  -0.51  0.45  -0.53  0.54  -0.47  0.40  0.96  0.46  -0.56  -0.42  0.51  -0.45  0.52  -0.42  -0.67  0.45  -0.59  -0.48  -0.66  0.48  0.54  -0.62  0.48  0.38  0.54  0.36  -0.44  0.58  -0.56  0.62  0.39  0.37  -0.48  -0.67  -0.50  0.47  -0.63  0.53  0.42  -1.02  -0.69  -0.46  -0.54  -0.62  -0.72  1.53  0.57  0.50  -0.84  0.46  0.32  0.53  0.40  -0.41  0.44  0.54  -0.79  -0.50  -0.34  0.37  -0.39  0.40  -0.44  0.45  -0.63  -0.35  0.45  -0.73  0.70  0.40  0.69  -0.44  0.32  0.42  -0.47  -0.46  0.86  -0.32  0.37  -0.51  -0.46  -0.62  -0.35  -0.48  -0.39  -0.52  0.94  -0.42  -0.78  -0.50 | 3.26E-09  4.93E-09  1.92E-07  3.67E-07  7.66E-07  9.65E-07  9.94E-07  1.10E-06  1.83E-06  2.38E-06  5.36E-06  5.41E-06  6.53E-06  9.00E-06  1.01E-05  1.36E-05  1.56E-05  1.94E-05  2.15E-05  2.20E-05  3.56E-05  3.87E-05  3.89E-05  3.96E-05  4.14E-05  4.44E-05  4.91E-05  5.05E-05  5.08E-05  5.33E-05  5.40E-05  5.51E-05  5.64E-05  6.03E-05  6.35E-05  6.65E-05  6.90E-05  7.06E-05  8.36E-05  9.12E-05  9.90E-05  9.91E-05  1.04E-04  1.29E-04  1.40E-04  1.42E-04  1.51E-04  1.53E-04  1.56E-04  1.61E-04  1.66E-04  1.77E-04  1.84E-04  1.84E-04  1.97E-04  1.99E-04  2.11E-04  2.11E-04  2.21E-04  2.25E-04  2.32E-04  2.34E-04  2.36E-04  2.46E-04  2.47E-04  2.49E-04  2.68E-04  2.74E-04  2.81E-04  2.82E-04  3.08E-04  3.15E-04  3.20E-04  3.24E-04  3.37E-04  3.41E-04  3.50E-04  3.51E-04  3.58E-04  3.64E-04  3.64E-04  3.92E-04  4.14E-04  4.20E-04  4.42E-04  4.50E-04  4.61E-04  4.66E-04  4.71E-04  4.79E-04  5.10E-04  5.22E-04  5.33E-04  5.45E-04  5.54E-04  5.56E-04  5.82E-04  5.88E-04  5.93E-04  5.96E-04  5.99E-04  6.02E-04  6.23E-04  6.24E-04  6.31E-04  6.37E-04  6.41E-04  6.42E-04  6.57E-04  6.74E-04  6.96E-04  7.01E-04  7.05E-04  7.42E-04  7.70E-04  7.78E-04  7.87E-04  7.88E-04  7.90E-04  7.91E-04  8.49E-04  8.52E-04  8.57E-04  8.60E-04  8.62E-04  8.62E-04 | 1.70E-05  1.70E-05  4.40E-04  6.32E-04  9.48E-04  9.48E-04  9.48E-04  9.48E-04  1.40E-03  1.64E-03  3.10E-03  3.10E-03  3.46E-03  4.43E-03  4.65E-03  5.85E-03  6.33E-03  7.42E-03  7.57E-03  7.57E-03  1.14E-02  1.14E-02  1.14E-02  1.14E-02  1.14E-02  1.18E-02  1.18E-02  1.18E-02  1.18E-02  1.18E-02  1.18E-02  1.18E-02  1.18E-02  1.22E-02  1.25E-02  1.27E-02  1.28E-02  1.28E-02  1.48E-02  1.57E-02  1.62E-02  1.62E-02  1.66E-02  2.02E-02  2.13E-02  2.13E-02  2.20E-02  2.20E-02  2.20E-02  2.21E-02  2.24E-02  2.34E-02  2.35E-02  2.35E-02  2.44E-02  2.44E-02  2.51E-02  2.51E-02  2.58E-02  2.58E-02  2.58E-02  2.58E-02  2.58E-02  2.60E-02  2.60E-02  2.60E-02  2.76E-02  2.77E-02  2.77E-02  2.77E-02  2.99E-02  3.01E-02  3.01E-02  3.01E-02  3.09E-02  3.09E-02  3.09E-02  3.09E-02  3.09E-02  3.09E-02  3.09E-02  3.29E-02  3.43E-02  3.44E-02  3.58E-02  3.61E-02  3.65E-02  3.65E-02  3.65E-02  3.66E-02  3.86E-02  3.91E-02  3.94E-02  3.99E-02  3.99E-02  3.99E-02  4.06E-02  4.06E-02  4.06E-02  4.06E-02  4.06E-02  4.06E-02  4.10E-02  4.10E-02  4.10E-02  4.10E-02  4.10E-02  4.10E-02  4.15E-02  4.22E-02  4.30E-02  4.30E-02  4.30E-02  4.48E-02  4.54E-02  4.54E-02  4.54E-02  4.54E-02  4.54E-02  4.54E-02  4.71E-02  4.71E-02  4.71E-02  4.71E-02  4.71E-02  4.71E-02 |
| **Ventral Striatum** | | | | | | |
| **Gene name** | **Ensembl ID** | | **Fold change** | **Log2 fold change** | **pval** | **adj.pval** |
| Erbb2ip  Bcas1os1  Secisbp2l  Gm20463  4931403E22Rik  Gm7887  Mrpl36  Eif3a  mt-Ti  Nsrp1 | ENSMUSG00000021709  ENSMUSG00000086970  ENSMUSG00000035093  ENSMUSG00000092395  ENSMUSG00000093772  ENSMUSG00000044211  ENSMUSG00000021607  ENSMUSG00000024991  ENSMUSG00000064342  ENSMUSG00000037958 | | 1.92  2.49  1.72  -1.69  1.96  2.70  -2.02  1.75  -3.32  1.80 | 0.94  1.32  0.78  -0.76  0.97  1.43  -1.01  0.81  -1.73  0.85 | 6.11E-07  9.66E-07  5.18E-06  8.63E-06  1.50E-05  2.54E-05  3.50E-05  3.62E-05  4.73E-05  5.43E-05 | 3.63E-03  3.63E-03  1.30E-02  1.62E-02  2.26E-02  3.18E-02  3.40E-02  3.40E-02  3.95E-02  4.08E-02 |

**(D)** Differentially expressed genes between female Tg and WT groups

| **Amygdala** | | | | | | |
| --- | --- | --- | --- | --- | --- | --- |
| **Gene name** | **Ensembl ID** | | **Fold change** | **Log2 fold change** | **pval** | **adj.pval** |
| Gm2115  Kcna5  Sel1l3  Lbhd2  Neurod6  Krt12  Vegfd  Lefty1  Myh9  Gpr161  Susd2  Mst1r  Tle3  C130093G08Rik  Vwa5b1  Zbtb7c  Ndrg1  Cachd1  Fibcd1  Cacng6  Prdm8  Slc8a3  Fancd2  Fezf1  Cmbl  Ucn3  Hcrtr1  Foxp1  Rspo2  Nr4a2  Adra1d  Klhl14  Kcns3  Col11a1  Nppc  Jun  Gm9924  Vsnl1  Vxn  Dcn  Slc9a2  Rprm  Akap13  Hpcal1  Stard5  Cartpt  Klhdc8a  Ccdc88c  Anln  Ksr1  Chrm5  Ngb  Col6a3  Cgnl1  Slc36a1  Aldh3b2 | ENSMUSG00000097789  ENSMUSG00000045534  ENSMUSG00000029189  ENSMUSG00000087075  ENSMUSG00000037984  ENSMUSG00000020912  ENSMUSG00000031380  ENSMUSG00000038793  ENSMUSG00000022443  ENSMUSG00000040836  ENSMUSG00000006342  ENSMUSG00000032584  ENSMUSG00000032280  ENSMUSG00000106775  ENSMUSG00000028753  ENSMUSG00000044646  ENSMUSG00000005125  ENSMUSG00000028532  ENSMUSG00000026841  ENSMUSG00000078815  ENSMUSG00000035456  ENSMUSG00000079055  ENSMUSG00000034023  ENSMUSG00000029697  ENSMUSG00000022235  ENSMUSG00000044988  ENSMUSG00000028778  ENSMUSG00000030067  ENSMUSG00000051920  ENSMUSG00000026826  ENSMUSG00000027335  ENSMUSG00000042514  ENSMUSG00000043673  ENSMUSG00000027966  ENSMUSG00000026241  ENSMUSG00000052684  ENSMUSG00000104299  ENSMUSG00000054459  ENSMUSG00000067879  ENSMUSG00000019929  ENSMUSG00000026062  ENSMUSG00000075334  ENSMUSG00000066406  ENSMUSG00000071379  ENSMUSG00000046027  ENSMUSG00000021647  ENSMUSG00000042115  ENSMUSG00000021182  ENSMUSG00000036777  ENSMUSG00000018334  ENSMUSG00000074939  ENSMUSG00000021032  ENSMUSG00000048126  ENSMUSG00000032232  ENSMUSG00000020261  ENSMUSG00000075296 | | -0.08  3.02  -0.55  7.20  -0.29  2.43  -0.12  -0.21  -0.64  -0.29  2.62  -0.35  -0.59  96.72  2.80  1.94  -0.43  -0.48  -0.14  2.63  1.68  1.54  -0.52  13.96  1.60  66.22  3.15  -0.53  -0.43  -0.37  -0.35  -0.43  -0.45  -0.38  1.82  -0.57  2.56  1.54  -0.71  -0.24  -0.53  1.54  -0.40  1.74  -0.42  3.85  2.57  -0.55  -0.33  -0.47  -0.16  2.49  -0.59  -0.33  -0.68  2.25 | -3.67  1.59  -0.87  2.85  -1.78  1.28  -3.00  -2.26  -0.65  -1.76  1.39  -1.50  -0.77  6.60  1.48  0.96  -1.22  -1.06  -2.82  1.40  0.75  0.62  -0.95  3.80  0.68  6.05  1.65  -0.93  -1.22  -1.43  -1.50  -1.22  -1.14  -1.39  0.86  -0.82  1.36  0.62  -0.50  -2.08  -0.91  0.63  -1.31  0.80  -1.26  1.94  1.36  -0.86  -1.62  -1.10  -2.65  1.32  -0.76  -1.61  -0.56  1.17 | 2.16E-21  4.93E-08  5.16E-08  6.10E-08  2.28E-07  3.97E-07  7.25E-07  8.29E-07  1.13E-06  1.27E-06  1.38E-06  1.48E-06  1.57E-06  1.60E-06  2.62E-06  3.13E-06  4.15E-06  5.37E-06  9.73E-06  1.37E-05  1.41E-05  2.13E-05  2.34E-05  2.43E-05  2.64E-05  2.92E-05  3.31E-05  3.42E-05  3.47E-05  3.71E-05  4.38E-05  4.42E-05  4.63E-05  5.35E-05  5.56E-05  5.82E-05  6.05E-05  6.72E-05  6.85E-05  7.25E-05  7.37E-05  8.02E-05  8.52E-05  8.55E-05  9.27E-05  1.00E-04  1.02E-04  1.11E-04  1.12E-04  1.23E-04  1.28E-04  1.30E-04  1.32E-04  1.33E-04  1.34E-04  1.60E-04 | 3.77E-17  2.65E-04  2.65E-04  2.65E-04  7.92E-04  1.15E-03  1.80E-03  1.80E-03  1.99E-03  1.99E-03  1.99E-03  1.99E-03  1.99E-03  1.99E-03  3.04E-03  3.40E-03  4.25E-03  5.19E-03  8.92E-03  1.17E-02  1.17E-02  1.68E-02  1.76E-02  1.76E-02  1.84E-02  1.96E-02  2.08E-02  2.08E-02  2.08E-02  2.15E-02  2.40E-02  2.40E-02  2.44E-02  2.74E-02  2.77E-02  2.82E-02  2.85E-02  3.06E-02  3.06E-02  3.13E-02  3.13E-02  3.33E-02  3.38E-02  3.38E-02  3.59E-02  3.77E-02  3.77E-02  3.99E-02  3.99E-02  4.23E-02  4.23E-02  4.23E-02  4.23E-02  4.23E-02  4.23E-02  4.99E-02 |
| **Hypothalamus** | | | | | | |
| **Gene name** | | **Ensembl ID** | **Fold change** | **Log2 fold change** | **pval** | **adj.pval** |
| n/a | | n/a | n/a | n/a | n/a | n/a |
| **Hippocampus** | | | | | | |
| **Gene name** | **Ensembl ID** | | **Fold change** | **Log2 fold change** | **pval** | **adj.pval** |
| Fosl2  Ret  Scn4b  Sstr1  Slc16a2  Adcyap1  Tead1  Car12  Dio3  Hr  Hap1  Opalin  C2cd4c  AW551984  Itga7  Tspan33  Inf2  Gabra3  Hpcal4  Lypd1  Efnb2  Baiap3  Mamld1  Mag  Zcchc12  Srsf5  Npr3  Col11a2  Cacng5  Ngb  Kcnk2  Grin3a  BC067074  Xbp1  Ccdc3  Gm43154  Plxnb2  Tmem141  Acan  Gh  Vgll3  Trhr  Ago2  Nr4a2  Sec14l5  Pcyt2  Tmem255a  Cadm1  Kif19a  Smo  Lingo3  Pon3  Adgra1  Cmtm4  B3galt2  Mcam  Iffo1  Gpr62  Tnfaip8  Krt2  Tenm2  Gda  Josd2  Syt9  Vwc2l  Magel2  Phlpp2  Ndnf  Ptprf  Foxo1  Ndst3  Vegfd  Atp2b4  Tmem125  Mvd  Lgi3  Nkx6-2  Wscd1  Cnp  Lct  Trpv6  Ubl3  Kit  Sfrp1  Ifi27  Rgs4  Tenm4  Il33  Caln1  Tjap1  Timp2  Ifit1  Frmpd3  Otof  Csrp1  Ephb1  Tmem63a  Slc7a3  Nkd2  Syt17  Phldb1  Adamtsl1  Cldn14  Stk32b  Medag  Galnt6  Tsc22d4  Trpc7  Rxrg  Bst2  Kcnc4  Dpy19l3  38231  Car10  Spint1  Rprm  Grp  D430041D05Rik  Reln  Tenm1  Chst2  Olig1  Map3k15  Rxra  Erbb3  Ssbp2  Aatk  Pdlim2  Pcdh11x  Arhgap23  Kcnd3  Ankrd6  Calb2  Rem2  Ttyh2  Slit3 | ENSMUSG00000029135  ENSMUSG00000030110  ENSMUSG00000046480  ENSMUSG00000035431  ENSMUSG00000033965  ENSMUSG00000024256  ENSMUSG00000055320  ENSMUSG00000032373  ENSMUSG00000075707  ENSMUSG00000022096  ENSMUSG00000006930  ENSMUSG00000050121  ENSMUSG00000045912  ENSMUSG00000038112  ENSMUSG00000025348  ENSMUSG00000001763  ENSMUSG00000037679  ENSMUSG00000031343  ENSMUSG00000046093  ENSMUSG00000026344  ENSMUSG00000001300  ENSMUSG00000047507  ENSMUSG00000059401  ENSMUSG00000036634  ENSMUSG00000036699  ENSMUSG00000021134  ENSMUSG00000022206  ENSMUSG00000024330  ENSMUSG00000040373  ENSMUSG00000021032  ENSMUSG00000037624  ENSMUSG00000039579  ENSMUSG00000021763  ENSMUSG00000020484  ENSMUSG00000026676  ENSMUSG00000086804  ENSMUSG00000036606  ENSMUSG00000026939  ENSMUSG00000030607  ENSMUSG00000020713  ENSMUSG00000091243  ENSMUSG00000038760  ENSMUSG00000036698  ENSMUSG00000026826  ENSMUSG00000091712  ENSMUSG00000025137  ENSMUSG00000036502  ENSMUSG00000032076  ENSMUSG00000010021  ENSMUSG00000001761  ENSMUSG00000051067  ENSMUSG00000029759  ENSMUSG00000025475  ENSMUSG00000096188  ENSMUSG00000033849  ENSMUSG00000032135  ENSMUSG00000038271  ENSMUSG00000091735  ENSMUSG00000062210  ENSMUSG00000064201  ENSMUSG00000049336  ENSMUSG00000058624  ENSMUSG00000038695  ENSMUSG00000062542  ENSMUSG00000045648  ENSMUSG00000056972  ENSMUSG00000031732  ENSMUSG00000049001  ENSMUSG00000033295  ENSMUSG00000044167  ENSMUSG00000027977  ENSMUSG00000031380  ENSMUSG00000026463  ENSMUSG00000050854  ENSMUSG00000006517  ENSMUSG00000033595  ENSMUSG00000041309  ENSMUSG00000020811  ENSMUSG00000006782  ENSMUSG00000026354  ENSMUSG00000029868  ENSMUSG00000001687  ENSMUSG00000005672  ENSMUSG00000031548  ENSMUSG00000064215  ENSMUSG00000038530  ENSMUSG00000048078  ENSMUSG00000024810  ENSMUSG00000060371  ENSMUSG00000012296  ENSMUSG00000017466  ENSMUSG00000034459  ENSMUSG00000042425  ENSMUSG00000062372  ENSMUSG00000026421  ENSMUSG00000032537  ENSMUSG00000026519  ENSMUSG00000031297  ENSMUSG00000021567  ENSMUSG00000058420  ENSMUSG00000048537  ENSMUSG00000066113  ENSMUSG00000047109  ENSMUSG00000029123  ENSMUSG00000029659  ENSMUSG00000037280  ENSMUSG00000029723  ENSMUSG00000021541  ENSMUSG00000015843  ENSMUSG00000046718  ENSMUSG00000027895  ENSMUSG00000043671  ENSMUSG00000020486  ENSMUSG00000056158  ENSMUSG00000027315  ENSMUSG00000075334  ENSMUSG00000024517  ENSMUSG00000068373  ENSMUSG00000042453  ENSMUSG00000016150  ENSMUSG00000033350  ENSMUSG00000046160  ENSMUSG00000031303  ENSMUSG00000015846  ENSMUSG00000018166  ENSMUSG00000003992  ENSMUSG00000025375  ENSMUSG00000022090  ENSMUSG00000034755  ENSMUSG00000049807  ENSMUSG00000040896  ENSMUSG00000040183  ENSMUSG00000003657  ENSMUSG00000022176  ENSMUSG00000034714  ENSMUSG00000056427 | | -0.47  -0.57  1.76  -0.46  -0.64  -0.23  -0.65  -0.48  -0.08  1.95  -0.52  1.89  -0.47  -0.36  1.90  -0.64  1.67  -0.52  -0.80  -0.72  -0.56  -0.41  -0.72  1.75  -0.59  1.24  -0.55  1.49  1.91  -0.32  1.39  -0.62  -0.48  -0.74  1.90  2.49  -0.78  1.40  1.88  -0.01  -0.08  -0.10  -0.79  -0.69  1.79  1.33  -0.51  -0.68  1.58  -0.67  1.64  -0.43  -0.55  -0.68  -0.68  1.59  1.30  1.63  -0.58  -0.34  -0.72  -0.70  1.42  -0.46  -0.34  -0.34  -0.81  -0.72  -0.74  -0.68  -0.53  -0.30  -0.36  1.80  1.41  1.34  1.94  1.37  1.64  1.74  -0.51  1.21  -0.67  -0.54  -0.56  -0.82  -0.66  -0.52  -0.73  1.48  -0.46  -0.21  -0.71  -0.31  1.35  1.51  1.57  -0.43  -0.60  -0.44  1.48  -0.56  2.58  -0.64  2.35  1.75  1.28  -0.41  -0.31  -0.32  -0.66  -0.83  1.47  -0.72  -0.25  -0.50  -0.22  -0.80  -0.81  -0.67  1.27  1.40  2.61  -0.74  1.82  -0.76  1.20  1.71  -0.65  1.27  -0.73  -0.77  -0.23  1.55  1.41  -0.76 | -1.10  -0.80  0.82  -1.11  -0.65  -2.10  -0.62  -1.07  -3.59  0.97  -0.95  0.92  -1.09  -1.47  0.92  -0.65  0.74  -0.95  -0.32  -0.47  -0.83  -1.27  -0.48  0.80  -0.75  0.31  -0.87  0.58  0.93  -1.66  0.47  -0.68  -1.07  -0.43  0.93  1.32  -0.35  0.49  0.91  -6.20  -3.70  -3.36  -0.34  -0.54  0.84  0.41  -0.98  -0.55  0.66  -0.58  0.71  -1.21  -0.85  -0.56  -0.56  0.67  0.38  0.70  -0.79  -1.55  -0.48  -0.52  0.51  -1.12  -1.56  -1.55  -0.30  -0.47  -0.44  -0.55  -0.91  -1.72  -1.47  0.85  0.49  0.42  0.95  0.45  0.72  0.80  -0.98  0.27  -0.58  -0.88  -0.82  -0.28  -0.59  -0.94  -0.46  0.56  -1.12  -2.27  -0.49  -1.70  0.43  0.59  0.65  -1.22  -0.74  -1.18  0.56  -0.85  1.37  -0.64  1.23  0.81  0.36  -1.28  -1.68  -1.65  -0.59  -0.27  0.55  -0.47  -2.02  -1.00  -2.19  -0.32  -0.30  -0.58  0.34  0.49  1.38  -0.43  0.86  -0.40  0.26  0.78  -0.63  0.34  -0.45  -0.38  -2.12  0.63  0.50  -0.41 | 2.34E-10  8.01E-09  8.07E-09  8.70E-09  1.56E-08  1.93E-08  3.58E-08  4.26E-08  4.31E-08  5.77E-07  5.89E-07  1.20E-06  1.30E-06  1.54E-06  1.57E-06  2.29E-06  3.92E-06  4.40E-06  4.67E-06  5.29E-06  6.61E-06  9.17E-06  9.78E-06  1.06E-05  1.13E-05  1.24E-05  1.26E-05  1.32E-05  1.54E-05  1.76E-05  1.77E-05  2.11E-05  2.27E-05  2.44E-05  2.57E-05  2.84E-05  3.06E-05  3.12E-05  3.19E-05  3.26E-05  3.57E-05  3.59E-05  3.63E-05  3.96E-05  4.45E-05  4.78E-05  4.84E-05  4.98E-05  5.25E-05  5.27E-05  6.62E-05  7.12E-05  7.39E-05  7.47E-05  7.98E-05  8.82E-05  9.02E-05  9.29E-05  9.49E-05  1.00E-04  1.01E-04  1.03E-04  1.11E-04  1.16E-04  1.19E-04  1.27E-04  1.32E-04  1.32E-04  1.35E-04  1.36E-04  1.42E-04  1.50E-04  1.55E-04  1.56E-04  1.66E-04  1.67E-04  1.68E-04  1.69E-04  1.70E-04  1.72E-04  1.73E-04  1.82E-04  1.88E-04  1.90E-04  1.91E-04  1.93E-04  1.94E-04  1.94E-04  1.95E-04  2.12E-04  2.22E-04  2.23E-04  2.24E-04  2.26E-04  2.28E-04  2.28E-04  2.30E-04  2.31E-04  2.34E-04  2.34E-04  2.48E-04  2.58E-04  2.70E-04  2.72E-04  2.73E-04  2.85E-04  2.85E-04  2.91E-04  2.99E-04  3.01E-04  3.15E-04  3.28E-04  3.30E-04  3.40E-04  3.45E-04  3.46E-04  3.50E-04  3.54E-04  3.54E-04  3.56E-04  3.60E-04  3.62E-04  3.64E-04  3.66E-04  3.80E-04  3.80E-04  3.83E-04  3.83E-04  3.84E-04  3.94E-04  3.94E-04  4.10E-04  4.32E-04  4.36E-04  4.50E-04  4.62E-04 | 3.44E-06  3.19E-05  3.19E-05  3.19E-05  4.57E-05  4.71E-05  7.02E-05  7.02E-05  7.02E-05  7.84E-04  7.84E-04  1.47E-03  1.47E-03  1.53E-03  1.53E-03  2.10E-03  3.38E-03  3.58E-03  3.61E-03  3.88E-03  4.62E-03  6.11E-03  6.23E-03  6.49E-03  6.63E-03  6.87E-03  6.87E-03  6.92E-03  7.80E-03  8.38E-03  8.38E-03  9.69E-03  1.01E-02  1.05E-02  1.08E-02  1.15E-02  1.19E-02  1.19E-02  1.19E-02  1.19E-02  1.24E-02  1.24E-02  1.24E-02  1.32E-02  1.45E-02  1.51E-02  1.51E-02  1.52E-02  1.54E-02  1.54E-02  1.90E-02  2.01E-02  2.03E-02  2.03E-02  2.13E-02  2.31E-02  2.32E-02  2.35E-02  2.36E-02  2.43E-02  2.43E-02  2.43E-02  2.59E-02  2.66E-02  2.68E-02  2.81E-02  2.84E-02  2.84E-02  2.84E-02  2.84E-02  2.94E-02  3.06E-02  3.09E-02  3.09E-02  3.13E-02  3.13E-02  3.13E-02  3.13E-02  3.13E-02  3.13E-02  3.13E-02  3.21E-02  3.21E-02  3.21E-02  3.21E-02  3.21E-02  3.21E-02  3.21E-02  3.21E-02  3.43E-02  3.43E-02  3.43E-02  3.43E-02  3.43E-02  3.43E-02  3.43E-02  3.43E-02  3.43E-02  3.43E-02  3.43E-02  3.60E-02  3.71E-02  3.81E-02  3.81E-02  3.81E-02  3.91E-02  3.91E-02  3.95E-02  4.01E-02  4.01E-02  4.17E-02  4.28E-02  4.28E-02  4.33E-02  4.33E-02  4.33E-02  4.33E-02  4.33E-02  4.33E-02  4.33E-02  4.33E-02  4.33E-02  4.33E-02  4.33E-02  4.36E-02  4.36E-02  4.36E-02  4.36E-02  4.36E-02  4.41E-02  4.41E-02  4.55E-02  4.76E-02  4.77E-02  4.89E-02  4.98E-02 |
| **Ventral Striatum** | | | | | | |
| **Gene name** | **Ensembl ID** | | **Fold change** | **Log2 fold change** | **pval** | **adj.pval** |
| Emp1  Lrrk1  Uaca  Flna | ENSMUSG00000030208  ENSMUSG00000015133  ENSMUSG00000034485  ENSMUSG00000031328 | | -0.42  -0.56  -0.56  -0.60 | -1.24  -0.85  -0.83  -0.74 | 7.16E-07  1.69E-06  1.95E-06  6.31E-06 | 1.17E-02  1.17E-02  1.17E-02  2.84E-02 |

**(E)** Differentially expressed genes between female Tg and NTg groups

| **Amygdala** | | | | | | |
| --- | --- | --- | --- | --- | --- | --- |
| **Gene name** | **Ensembl ID** | | **Fold change** | **Log2 fold change** | **pval** | **adj.pval** |
| Gm2115  Scn5a  Neurod6  C130093G08Rik  Anln  Adra1d  Stard5 | ENSMUSG00000097789  ENSMUSG00000032511  ENSMUSG00000037984  ENSMUSG00000106775  ENSMUSG00000036777  ENSMUSG00000027335  ENSMUSG00000046027 | | -0.13  2.91  -0.33  84.16  -0.27  -0.32  -0.36 | -2.99  1.54  -1.62  6.40  -1.90  -1.66  -1.45 | 6.43E-15  7.33E-08  2.43E-06  3.41E-06  6.04E-06  6.56E-06  6.78E-06 | 1.16E-10  6.64E-04  1.46E-02  1.54E-02  1.75E-02  1.75E-02  1.75E-02 |
| **Hypothalamus** | | | | | | |
| **Gene name** | | **Ensembl ID** | **Fold change** | **Log2 fold change** | **pval** | **adj.pval** |
| n/a | | n/a | n/a | n/a | n/a | n/a |
| **Hippocampus** | | | | | | |
| **Gene name** | **Ensembl ID** | | **Fold change** | **Log2 fold change** | **pval** | **adj.pval** |
| n/a | n/a | | n/a | n/a | n/a | n/a |
| **Ventral Striatum** | | | | | | |
| **Gene name** | **Ensembl ID** | | **Fold change** | **Log2 fold change** | **pval** | **adj.pval** |
| n/a | n/a | | n/a | n/a | n/a | n/a |

**(F)** Differentially expressed genes between female NTg and WT groups

| **Amygdala** | | | | | | |
| --- | --- | --- | --- | --- | --- | --- |
| **Gene name** | **Ensembl ID** | | **Fold change** | **Log2 fold change** | **pval** | **adj.pval** |
| Lrp1 | ENSMUSG00000040249 | | -0.65 | -0.61 | 2.18E-06 | 3.95E-02 |
| **Hypothalamus** | | | | | | |
| **Gene name** | | **Ensembl ID** | **Fold change** | **Log2 fold change** | **pval** | **adj.pval** |
| n/a | | n/a | n/a | n/a | n/a | n/a |
| **Hippocampus** | | | | | | |
| **Gene name** | **Ensembl ID** | | **Fold change** | **Log2 fold change** | **pval** | **adj.pval** |
| Rgs4  Adcyap1  Fosl2  Nr4a2  Mag  Sstr1  Vwc2l  Gabra3  Sec14l5  Fos  Dio3  Ret  Efnb2  Opalin  Tjap1  Itga7  Col11a2  Gm43154  AW551984  Pcsk5  Ndst3  Grem2  Grm8  Mirt1  Tmtc4  Satb2  Pcdh11x  Tmem141  Kif19a  Gm3764  B3galt2  Ttyh2  Erbb3  Baiap3  Inf2  Bhlhe40  Grin3a  Cnp  Tsc22d4  Scn4b  Tmem63a  Trhr  Pdlim2  Hpcal4 | ENSMUSG00000038530  ENSMUSG00000024256  ENSMUSG00000029135  ENSMUSG00000026826  ENSMUSG00000036634  ENSMUSG00000035431  ENSMUSG00000045648  ENSMUSG00000031343  ENSMUSG00000091712  ENSMUSG00000021250  ENSMUSG00000075707  ENSMUSG00000030110  ENSMUSG00000001300  ENSMUSG00000050121  ENSMUSG00000012296  ENSMUSG00000025348  ENSMUSG00000024330  ENSMUSG00000086804  ENSMUSG00000038112  ENSMUSG00000024713  ENSMUSG00000027977  ENSMUSG00000050069  ENSMUSG00000024211  ENSMUSG00000097636  ENSMUSG00000041594  ENSMUSG00000038331  ENSMUSG00000034755  ENSMUSG00000026939  ENSMUSG00000010021  ENSMUSG00000097156  ENSMUSG00000033849  ENSMUSG00000034714  ENSMUSG00000018166  ENSMUSG00000047507  ENSMUSG00000037679  ENSMUSG00000030103  ENSMUSG00000039579  ENSMUSG00000006782  ENSMUSG00000029723  ENSMUSG00000046480  ENSMUSG00000026519  ENSMUSG00000038760  ENSMUSG00000022090  ENSMUSG00000046093 | | -0.72  -0.20  -0.48  -0.61  1.87  -0.52  -0.26  -0.50  1.98  -0.52  -0.13  -0.65  -0.57  1.78  1.59  1.79  1.49  2.58  -0.40  -0.57  -0.49  -0.70  -0.34  -0.34  -0.64  -0.58  -0.60  1.39  1.57  1.41  -0.67  1.48  1.94  -0.46  1.55  -0.71  -0.65  1.67  1.30  1.45  1.60  -0.12  1.78  -0.83 | -0.47  -2.29  -1.06  -0.72  0.90  -0.93  -1.94  -0.99  0.98  -0.95  -2.96  -0.62  -0.81  0.83  0.67  0.84  0.58  1.37  -1.32  -0.80  -1.02  -0.51  -1.56  -1.55  -0.64  -0.77  -0.73  0.48  0.65  0.50  -0.57  0.56  0.95  -1.13  0.63  -0.49  -0.62  0.74  0.38  0.54  0.68  -3.11  0.84  -0.26 | 3.21E-10  9.15E-10  1.08E-09  3.91E-08  7.40E-07  1.59E-06  1.63E-06  1.68E-06  1.85E-06  6.58E-06  7.38E-06  1.00E-05  1.10E-05  1.13E-05  1.15E-05  1.18E-05  1.37E-05  1.50E-05  1.74E-05  1.79E-05  2.00E-05  2.17E-05  2.70E-05  2.84E-05  3.02E-05  3.14E-05  3.55E-05  4.20E-05  6.29E-05  6.48E-05  6.66E-05  8.05E-05  8.28E-05  8.96E-05  8.97E-05  1.09E-04  1.10E-04  1.13E-04  1.16E-04  1.26E-04  1.27E-04  1.30E-04  1.35E-04  1.55E-04 | 4.38E-06  4.93E-06  4.93E-06  1.33E-04  2.02E-03  2.81E-03  2.81E-03  2.81E-03  2.81E-03  8.99E-03  9.16E-03  1.01E-02  1.01E-02  1.01E-02  1.01E-02  1.01E-02  1.10E-02  1.14E-02  1.22E-02  1.22E-02  1.30E-02  1.35E-02  1.60E-02  1.61E-02  1.65E-02  1.65E-02  1.80E-02  2.05E-02  2.93E-02  2.93E-02  2.93E-02  3.42E-02  3.42E-02  3.50E-02  3.50E-02  4.04E-02  4.04E-02  4.04E-02  4.06E-02  4.24E-02  4.24E-02  4.24E-02  4.29E-02  4.82E-02 |
| **Ventral Striatum** | | | | | | |
| **Gene name** | **Ensembl ID** | | **Fold change** | **Log2 fold change** | **pval** | **adj.pval** |
| n/a | n/a | | n/a | n/a | n/a | n/a |

**Supplemental Table S3** Shared DEGs

**(A)** Genes shared between male Tg vs WT DEG lists and male Tg vs NTg DEG lists

| **Amygdala** | | | | | |
| --- | --- | --- | --- | --- | --- |
| **Gene name** | **Ensembl ID** | **Fold change** | **Log2 fold change** | **Pval** | **adj.pval** |
| Ttr | ENSMUSG00000061808 | 193.6 | 7.6 | 8.18E-11 | 5.43E-06 |
| **Hypothalamus** | | | | | |
| **Gene name** | **Ensembl ID** | **Fold change** | **Log2 fold change** | **Pval** | **adj.pval** |
| Zfr2  Mir770  Agrn  Unc13a  Bzrap1  Celsr3  Kmt2b  Sema6c  Map4k2  Gcn1l1  Sptbn4  Plekhn1  Gpc1  Pisd-ps1  Zswim8  Crocc  Col16a1  Dot1l  Adam11  Zfp512b  Plxna3  Setd1a  Lrrc16b  Fcho1  Col4a2  Speg  Slc4a3  PISD  Col11a2  Epha10  Gigyf1  Gm996  Gm26702  P3h3  Cacna1g  Mroh1  Dgkz  Tmem191c  Cacna1h  Lmtk3  Vwa5b2  Sema4g  Myo9b  Cyth1  Arhgap33  Pnrc2  Pkd1  5031439G07Rik  Flna  Dync1h1  Zfp598  B4galnt4  Col4a1  Slc16a11  RP23-325D10.3  Scrib  Mbd6  Vps9d1  Ptpru  Phc1  Colgalt1  Prpf40b  Cbx7  Gm26377  Inha  Edc4  Slc9a5  Atp8b2  Zfp384  Ltbp4  Zgpat  Calb1  Ankrd24  Ptpn23  Phldb1  Usp19  Srrm2  Rap1gap  Nckap5l  Fbxl19  Kcnt1  Vars  Atg2a  Dstn  Fam193b  Dennd6b  Srrm1  Ercc2  Nktr  BC037034  Man2c1  Megf8  Hdac7  Arhgef1  Zfp523  Snapc4  Repin1  Clcn2  Dvl1  Gm16740  Tbkbp1  Thbs3  Ppfia4  Impa1  Fbrs  Chn1  B3gat1  Ssr3  Adgrb1  Pid1  Morf4l1  Rdh13 | ENSMUSG00000034949  ENSMUSG00000076451  ENSMUSG00000041936  ENSMUSG00000034799  ENSMUSG00000034156  ENSMUSG00000023473  ENSMUSG00000006307  ENSMUSG00000038777  ENSMUSG00000024948  ENSMUSG00000041638  ENSMUSG00000011751  ENSMUSG00000078485  ENSMUSG00000034220  ENSMUSG00000082286  ENSMUSG00000021819  ENSMUSG00000040860  ENSMUSG00000040690  ENSMUSG00000061589  ENSMUSG00000020926  ENSMUSG00000000823  ENSMUSG00000031398  ENSMUSG00000042308  ENSMUSG00000022211  ENSMUSG00000070000  ENSMUSG00000031503  ENSMUSG00000026207  ENSMUSG00000006576  ENSMUSG00000095041  ENSMUSG00000024330  ENSMUSG00000028876  ENSMUSG00000029714  ENSMUSG00000029419  ENSMUSG00000097872  ENSMUSG00000023191  ENSMUSG00000020866  ENSMUSG00000022558  ENSMUSG00000040479  ENSMUSG00000055692  ENSMUSG00000024112  ENSMUSG00000062044  ENSMUSG00000046613  ENSMUSG00000025207  ENSMUSG00000004677  ENSMUSG00000017132  ENSMUSG00000036882  ENSMUSG00000028675  ENSMUSG00000032855  ENSMUSG00000036046  ENSMUSG00000031328  ENSMUSG00000018707  ENSMUSG00000041130  ENSMUSG00000055629  ENSMUSG00000031502  ENSMUSG00000040938  ENSMUSG00000108815  ENSMUSG00000022568  ENSMUSG00000025409  ENSMUSG00000001062  ENSMUSG00000028909  ENSMUSG00000040669  ENSMUSG00000034807  ENSMUSG00000023007  ENSMUSG00000053411  ENSMUSG00000076138  ENSMUSG00000032968  ENSMUSG00000036270  ENSMUSG00000014786  ENSMUSG00000060671  ENSMUSG00000038346  ENSMUSG00000040488  ENSMUSG00000027582  ENSMUSG00000028222  ENSMUSG00000054708  ENSMUSG00000036057  ENSMUSG00000048537  ENSMUSG00000006676  ENSMUSG00000039218  ENSMUSG00000041351  ENSMUSG00000023009  ENSMUSG00000030811  ENSMUSG00000058740  ENSMUSG00000007029  ENSMUSG00000024773  ENSMUSG00000015932  ENSMUSG00000021495  ENSMUSG00000015377  ENSMUSG00000028809  ENSMUSG00000030400  ENSMUSG00000032525  ENSMUSG00000036948  ENSMUSG00000032295  ENSMUSG00000045039  ENSMUSG00000022475  ENSMUSG00000040940  ENSMUSG00000024220  ENSMUSG00000036281  ENSMUSG00000052751  ENSMUSG00000022843  ENSMUSG00000029071  ENSMUSG00000097167  ENSMUSG00000038517  ENSMUSG00000028047  ENSMUSG00000026458  ENSMUSG00000027531  ENSMUSG00000042423  ENSMUSG00000056486  ENSMUSG00000045994  ENSMUSG00000027828  ENSMUSG00000034730  ENSMUSG00000045658  ENSMUSG00000062270  ENSMUSG00000008435 | 1.48  2.81  1.53  1.47  1.82  1.74  1.50  1.68  1.67  1.44  1.58  2.17  1.60  2.29  1.40  1.77  2.22  1.72  1.40  1.58  1.49  1.42  1.70  1.58  1.76  1.50  1.40  2.21  2.02  1.65  1.74  1.36  1.35  2.00  1.67  1.36  1.54  1.49  1.40  1.43  1.67  1.68  1.69  1.41  1.53  -1.40  1.50  1.49  1.61  1.30  1.48  1.31  1.67  1.99  1.43  1.54  1.92  1.40  1.46  1.27  1.39  1.57  1.48  -1.78  1.92  1.28  1.59  1.28  1.51  1.45  1.71  -1.34  1.73  1.48  1.46  1.28  1.57  1.25  1.40  1.34  1.52  1.40  1.44  -1.34  2.04  1.64  1.35  1.67  1.56  1.36  1.51  1.19  1.82  1.74  1.62  1.71  1.35  1.48  1.45  -1.44  1.61  1.64  1.27  -1.35  1.34  -1.23  1.22  -1.28  1.27  -1.55  -1.24  1.35 | 0.56  1.49  0.61  0.56  0.86  0.80  0.58  0.75  0.74  0.52  0.66  1.12  0.68  1.19  0.48  0.82  1.15  0.78  0.48  0.66  0.58  0.51  0.76  0.65  0.81  0.58  0.49  1.14  1.01  0.72  0.80  0.44  0.44  1.00  0.74  0.44  0.62  0.57  0.48  0.52  0.74  0.75  0.76  0.49  0.61  -0.49  0.58  0.58  0.69  0.37  0.57  0.39  0.74  0.99  0.51  0.63  0.94  0.49  0.55  0.35  0.47  0.65  0.57  -0.82  0.94  0.35  0.67  0.35  0.59  0.54  0.76  -0.42  0.79  0.56  0.54  0.36  0.65  0.32  0.49  0.42  0.60  0.48  0.52  -0.43  1.01  0.71  0.43  0.74  0.64  0.44  0.59  0.26  0.86  0.79  0.69  0.77  0.44  0.57  0.54  -0.52  0.69  0.71  0.34  -0.43  0.43  -0.30  0.29  -0.35  0.34  -0.63  -0.31  0.43 | 1.84E-08  1.56E-06  1.67E-07  1.83E-07  2.99E-07  3.30E-07  2.09E-06  1.40E-06  9.37E-07  3.44E-06  1.69E-06  1.93E-05  6.72E-06  1.12E-04  6.19E-06  4.07E-06  4.87E-06  5.41E-06  1.05E-05  6.94E-06  6.40E-06  8.97E-06  1.21E-05  1.30E-05  1.27E-05  1.23E-05  2.26E-05  1.43E-05  1.30E-05  2.86E-05  1.77E-05  2.30E-05  2.49E-05  2.39E-05  2.45E-05  3.87E-05  2.95E-05  4.25E-05  4.46E-05  4.57E-05  5.55E-05  5.70E-05  5.54E-05  8.53E-05  6.36E-05  7.97E-05  6.35E-05  7.17E-05  6.57E-05  1.33E-04  7.66E-05  7.43E-05  9.09E-05  8.83E-05  8.61E-05  1.31E-04  1.22E-04  1.16E-04  1.76E-04  1.57E-04  1.39E-04  1.39E-04  1.36E-04  2.50E-04  1.54E-04  1.62E-04  2.01E-04  2.39E-04  1.90E-04  1.86E-04  2.39E-04  2.38E-04  2.79E-04  2.48E-04  2.67E-04  3.28E-04  3.27E-04  3.02E-04  3.77E-04  3.16E-04  3.49E-04  5.70E-04  5.34E-04  3.77E-04  4.35E-04  4.34E-04  4.17E-04  4.14E-04  4.71E-04  4.14E-04  4.71E-04  5.60E-04  4.89E-04  5.55E-04  5.27E-04  5.39E-04  6.00E-04  5.26E-04  5.75E-04  9.28E-04  7.18E-04  7.31E-04  8.36E-04  7.87E-04  7.47E-04  7.53E-04  7.91E-04  8.69E-04  1.11E-03  1.11E-03  1.25E-03  1.30E-03 | 5.59E-05  6.70E-05  1.03E-04  1.38E-04  1.98E-04  2.09E-04  3.38E-04  3.78E-04  4.41E-04  5.30E-04  6.12E-04  6.44E-04  8.58E-04  8.63E-04  1.10E-03  1.18E-03  1.25E-03  1.27E-03  1.39E-03  1.44E-03  1.49E-03  1.67E-03  2.00E-03  2.01E-03  2.02E-03  2.04E-03  2.09E-03  2.14E-03  2.16E-03  2.23E-03  2.43E-03  2.58E-03  2.71E-03  2.96E-03  3.08E-03  3.42E-03  3.43E-03  4.23E-03  4.71E-03  4.78E-03  5.00E-03  5.07E-03  5.32E-03  5.47E-03  5.49E-03  5.53E-03  5.75E-03  5.82E-03  5.90E-03  6.21E-03  6.44E-03  6.45E-03  6.49E-03  6.69E-03  6.91E-03  7.94E-03  8.14E-03  8.20E-03  8.45E-03  8.60E-03  8.85E-03  9.06E-03  9.21E-03  9.49E-03  9.57E-03  9.75E-03  9.90E-03  1.07E-02  1.13E-02  1.15E-02  1.24E-02  1.28E-02  1.40E-02  1.41E-02  1.47E-02  1.57E-02  1.62E-02  1.62E-02  1.64E-02  1.64E-02  1.68E-02  1.79E-02  1.79E-02  1.85E-02  1.87E-02  1.92E-02  1.94E-02  1.94E-02  1.99E-02  1.99E-02  2.00E-02  2.02E-02  2.04E-02  2.12E-02  2.13E-02  2.20E-02  2.29E-02  2.30E-02  2.38E-02  2.40E-02  2.46E-02  2.47E-02  2.61E-02  2.61E-02  2.90E-02  2.95E-02  2.97E-02  3.24E-02  3.45E-02  3.71E-02  4.01E-02  4.11E-02 |
| **Hippocampus** | | | | | |
| **Gene name** | **Ensembl ID** | **Fold change** | **Log2 fold change** | **pval** | **adj.pval** |
| n/a | n/a | n/a | n/a | n/a | n/a |
| **Ventral Striatum** | | | | | |
| **Gene name** | **Ensembl ID** | **Fold change** | **Log2 fold change** | **pval** | **adj.pval** |
| n/a | n/a | n/a | n/a | n/a | n/a |

**(B)** Genes shared between female Tg vs WT DEG lists and female Tg vs NTg DEG lists

| **Amygdala** | | | | | |
| --- | --- | --- | --- | --- | --- |
| **Gene name** | **Ensembl ID** | **Fold change** | **Log2 fold change** | **Pval** | **adj.pval** |
| Gm2115  Neurod6  C130093G08Rik  Adra1d  Stard5  Anln | ENSMUSG00000097789  ENSMUSG00000037984  ENSMUSG00000106775  ENSMUSG00000027335  ENSMUSG00000046027  ENSMUSG00000036777 | -0.10  -0.31  90.23  -0.33  -0.39  -0.30 | -3.33  -1.70  6.50  -1.58  -1.36  -1.76 | 3.21E-15  1.33E-06  2.50E-06  2.52E-05  4.97E-05  5.92E-05 | 5.82E-11  7.72E-03  8.70E-03  2.08E-02  2.67E-02  2.87E-02 |
| **Hypothalamus** | | | | | |
| **Gene name** | **Ensembl ID** | **Fold change** | **Log2 fold change** | **Pval** | **adj.pval** |
| n/a | n/a | n/a | n/a | n/a | n/a |
| **Hippocampus** | | | | | |
| **Gene name** | **Ensembl ID** | **Fold change** | **Log2 fold change** | **pval** | **adj.pval** |
| n/a | n/a | n/a | n/a | n/a | n/a |
| **Ventral Striatum** | | | | | |
| **Gene name** | **Ensembl ID** | **Fold change** | **Log2 fold change** | **pval** | **adj.pval** |
| n/a | n/a | n/a | n/a | n/a | n/a |

**(C)** Genes shared between male Tg vs WT DEG lists and male NTg vs WT DEG lists

| **Amygdala** | | | | | | |
| --- | --- | --- | --- | --- | --- | --- |
| **Gene name** | **Ensembl ID** | | **Fold change** | **Log2 fold change** | **pval** | **adj.pval** |
| n/a | n/a | | n/a | n/a | n/a | n/a |
| **Hypothalamus** | | | | | | |
| **Gene name** | | **Ensembl ID** | **Fold change** | **Log2 fold change** | **pval** | **adj.pval** |
| n/a | | n/a | n/a | n/a | n/a | n/a |
| **Hippocampus** | | | | | | |
| **Gene name** | **Ensembl ID** | | **Fold change** | **Log2 fold change** | **pval** | **adj.pval** |
| Ablim1  Vstm2l  Ccar1  Rprml  Psd  Uqcr10  Cox8a  Tbr1  Rpl13  Malat1  Dgcr6  Ifitm10  Map1lc3a  4930402H24Rik  Ndufa11  Znhit2  Efr3a  Ndufs7  Prdx5 | ENSMUSG00000025085  ENSMUSG00000037843  ENSMUSG00000020074  ENSMUSG00000046215  ENSMUSG00000037126  ENSMUSG00000059534  ENSMUSG00000035885  ENSMUSG00000035033  ENSMUSG00000000740  ENSMUSG00000092341  ENSMUSG00000003531  ENSMUSG00000045777  ENSMUSG00000027602  ENSMUSG00000027309  ENSMUSG00000002379  ENSMUSG00000075227  ENSMUSG00000015002  ENSMUSG00000020153  ENSMUSG00000024953 | | 1.48  -1.59  1.55  -1.64  -1.42  -1.45  -1.43  1.55  -1.42  2.36  -1.45  -1.42  -1.38  1.38  -1.41  -1.59  1.37  -1.40  -1.34 | 0.56  -0.67  0.63  -0.71  -0.50  -0.53  -0.51  0.63  -0.50  1.21  -0.54  -0.50  -0.47  0.46  -0.50  -0.67  0.45  -0.49  -0.42 | 3.62E-05  3.61E-05  5.09E-05  7.30E-05  6.44E-05  5.80E-05  8.03E-05  6.73E-05  1.06E-04  4.29E-04  7.76E-05  9.61E-05  1.03E-04  1.17E-04  1.24E-04  1.65E-04  1.79E-04  2.06E-04  3.45E-04 | 1.88E-02  1.88E-02  2.01E-02  2.01E-02  2.04E-02  2.11E-02  2.12E-02  2.27E-02  2.32E-02  2.40E-02  2.55E-02  2.60E-02  2.77E-02  2.97E-02  3.13E-02  3.18E-02  3.44E-02  3.50E-02  4.15E-02 |
| **Ventral Striatum** | | | | | | |
| **Gene name** | **Ensembl ID** | | **Fold change** | **Log2 fold change** | **pval** | **adj.pval** |
| Erbb2ip  Secisbp2l | ENSMUSG00000021709  ENSMUSG00000035093 | | 1.80  1.65 | 0.85  0.73 | 3.82E-05  4.58E-05 | 2.55E-02  3.02E-02 |

**(D)** Genes shared between female Tg vs WT DEG lists and female NTg vs WT DEG lists

| **Amygdala** | | | | | | |
| --- | --- | --- | --- | --- | --- | --- |
| **Gene name** | **Ensembl ID** | | **Fold change** | **Log2 fold change** | **pval** | **adj.pval** |
| n/a | n/a | | n/a | n/a | n/a | n/a |
| **Hypothalamus** | | | | | | |
| **Gene name** | | **Ensembl ID** | **Fold change** | **Log2 fold change** | **pval** | **adj.pval** |
| n/a | | n/a | n/a | n/a | n/a | n/a |
| **Hippocampus** | | | | | | |
| **Gene name** | **Ensembl ID** | | **Fold change** | **Log2 fold change** | **pval** | **adj.pval** |
| Fosl2  Ret  Scn4b  Sstr1  Adcyap1  Dio3  Opalin  AW551984  Itga7  Inf2  Gabra3  Hpcal4  Efnb2  Baiap3  Mag  Col11a2  Grin3a  Gm43154  Tmem141  Trhr  Nr4a2  Sec14l5  Kif19a  B3galt2  Vwc2l  Ndst3  Cnp  Rgs4  Tjap1  Tmem63a  Tsc22d4  Erbb3  Pdlim2  Pcdh11x  Ttyh2 | ENSMUSG00000029135  ENSMUSG00000030110  ENSMUSG00000046480  ENSMUSG00000035431  ENSMUSG00000024256  ENSMUSG00000075707  ENSMUSG00000050121  ENSMUSG00000038112  ENSMUSG00000025348  ENSMUSG00000037679  ENSMUSG00000031343  ENSMUSG00000046093  ENSMUSG00000001300  ENSMUSG00000047507  ENSMUSG00000036634  ENSMUSG00000024330  ENSMUSG00000039579  ENSMUSG00000086804  ENSMUSG00000026939  ENSMUSG00000038760  ENSMUSG00000026826  ENSMUSG00000091712  ENSMUSG00000010021  ENSMUSG00000033849  ENSMUSG00000045648  ENSMUSG00000027977  ENSMUSG00000006782  ENSMUSG00000038530  ENSMUSG00000012296  ENSMUSG00000026519  ENSMUSG00000029723  ENSMUSG00000018166  ENSMUSG00000022090  ENSMUSG00000034755  ENSMUSG00000034714 | | -0.47  -0.61  1.61  -0.49  -0.22  -0.11  1.84  -0.38  1.84  1.61  -0.51  -0.82  -0.56  -0.44  1.81  1.49  -0.64  2.54  1.40  -0.11  -0.65  1.88  1.57  -0.68  -0.30  -0.51  1.66  -0.77  1.53  1.59  1.29  1.88  1.75  -0.62  1.44 | -1.08  -0.71  0.68  -1.02  -2.20  -3.28  0.88  -1.40  0.88  0.69  -0.97  -0.29  -0.82  -1.20  0.85  0.58  -0.65  1.34  0.48  -3.23  -0.63  0.91  0.65  -0.56  -1.75  -0.96  0.73  -0.37  0.61  0.67  0.37  0.91  0.81  -0.68  0.53 | 6.59E-10  5.02E-06  6.30E-05  7.99E-07  1.01E-08  3.71E-06  6.26E-06  9.47E-06  6.69E-06  4.68E-05  3.04E-06  8.00E-05  8.80E-06  4.94E-05  5.68E-06  1.35E-05  6.55E-05  2.17E-05  3.66E-05  8.31E-05  1.98E-05  2.32E-05  5.77E-05  7.32E-05  6.03E-05  8.11E-05  1.42E-04  9.65E-05  1.12E-04  1.79E-04  2.01E-04  2.31E-04  2.59E-04  2.10E-04  2.65E-04 | 4.18E-06  5.06E-03  2.12E-02  1.42E-03  2.60E-05  4.61E-03  5.77E-03  6.87E-03  5.81E-03  1.92E-02  3.20E-03  2.59E-02  7.35E-03  2.05E-02  4.26E-03  8.98E-03  2.51E-02  1.15E-02  1.62E-02  2.74E-02  6.66E-03  8.66E-03  2.24E-02  2.53E-02  1.48E-02  2.12E-02  3.59E-02  1.60E-02  2.22E-02  3.83E-02  3.99E-02  3.89E-02  4.32E-02  3.08E-02  4.16E-02 |
| **Ventral Striatum** | | | | | | |
| **Gene name** | **Ensembl ID** | | **Fold change** | **Log2 fold change** | **pval** | **adj.pval** |
| n/a | n/a | | n/a | n/a | n/a | n/a |

**Supplemental Table S4** Statistics

|  | **Light/dark box** | | | | | | | | | | | | | | | | | |
| --- | --- | --- | --- | --- | --- | --- | --- | --- | --- | --- | --- | --- | --- | --- | --- | --- | --- | --- |
| **Measure** | | ***N* (animals)** | **Mean ± SD** | **95% CI (lower; upper)** |  | **MV** | **d.f.** | **Residual** | **F** | **p** | **Effect**  **size** | **Post hoc tests (Multiple comparisons Tukey HSD)** | | | | | | |
|  |  |  |  |  |  |  |  |  |  |  |  |  | **t** | | **d.f.** | | **p** | **CI** |
| ***Crosses into light*** | | 40 WT | 26.03±5.59 | 24.27; 27.78 | 2-way ANOVA (GROUP)  Bonferroni correction | - | 2 | 113 | 5.15 | .007** | .817 | WT vs NTg | 2.99 | | 77 | | .009** | .777; 6.76 |
|  |  | 39 NTg | 22.26±5.14 | 20.48; 24.03 |  | 1 |  |  |  |  |  | WT vs Tg | 2.50 | | 78 | | .037* | .152; 6.10 |
|  |  | 40 Tg | 22.90±5.92 | 21.15; 24.65 |  | - |  |  |  |  |  | NTg vs Tg | 0.51 | | 77 | | .866 | -3.64; 2.35 |
|  |  | 60 F | 23.30±5.30 | 21.87; 24.73 | 2-way ANOVA (SEX) | - | 1 | 113 | .691 | .408 | .131 |  |  | |  | |  |  |
|  |  | 59 M | 24.15±6.20 | 22.71; 25.60 |  | 1 |  |  |  |  |  |  |  | |  | |  |  |
|  |  | 20 WT (F) | 25.70±5.62 | 23.22; 28.18 | 2-way ANOVA (GROUP*SEX) | - | 2 | 113 | .354 | .702 | .106 |  |  | |  | |  |  |
|  |  | 20 NTg (F) | 22.30±5.13 | 19.82; 24.78 |  | - |  |  |  |  |  |  |  | |  | |  |  |
|  |  | 20 Tg (F) | 21.90±4.49 | 19.42; 24.38 |  | - |  |  |  |  |  |  |  | |  | |  |  |
|  |  | 20 WT (M) | 26.35±5.68 | 23.87; 28.83 |  | - |  |  |  |  |  |  |  | |  | |  |  |
|  |  | 19 NTg (M) | 22.21±5.29 | 19.67; 24.76 |  | 1 |  |  |  |  |  |  |  | |  | |  |  |
|  | | 20 Tg (M) | 23.90±7.04 | 21.42; 26.38 |  | - |  |  |  |  |  |  |  | |  | |  |  |
|  | | 20 WT (F) | 25.70±5.62 | 23.42; 27.99 | 1-way ANOVA (F)  Bonferroni correction | - | 2 | 57 | 3.35 | .042* | .610 | WT vs NTg | 2.11 | | 38 | | .097 | -.483; 7.28 |
|  |  | 20 NTg (F) | 22.30±5.13 | 20.02; 24.59 |  | - |  |  |  |  |  | WT vs Tg | 2.35 | | 38 | | .056 | -.083; 7.68 |
|  |  | 20 Tg (F) | 21.90±4.49 | 19.62; 24.19 |  | - |  |  |  |  |  | NTg vs Tg | 0.25 | | 38 | | .967 | -3.48; 4.28 |
|  | | 20 WT (M) | 26.35±5.68 | 23.64; 29.07 | 1-way ANOVA (M)  Bonferroni correction | - | 2 | 56 | 2.36 | .104 | .449 |  |  | |  | |  |  |
|  |  | 19 NTg (M) | 22.21±5.29 | 19.43; 25.00 |  | 1 |  |  |  |  |  |  |  | |  | |  |  |
|  |  | 20 Tg ((M) | 23.90±7.04 | 21.19; 26.62 |  | - |  |  |  |  |  |  |  | |  | |  |  |
| ***Time in light*** | | 40 WT | 95.49±21.9 | 88.22; 102.76 | 2-way ANOVA (GROUP)  Bonferroni correction | - | 2 | 113 | 2.06 | .132 | .454 |  |  | |  | |  |  |
|  |  | 39 NTg | 92.69±26.9 | 85.36; 100.09 |  | 1 |  |  |  |  |  |  |  | |  | |  |  |
|  |  | 40 Tg | 85.30±20.3 | 78.02; 92.57 |  | - |  |  |  |  |  |  |  | |  | |  |  |
|  |  | 60 F | 89.72±22.3 | 83.79; 95.66 | 2-way ANOVA (SEX) | - | 1 | 113 | 0.47 | .496 | .103 |  |  | |  | |  |  |
|  |  | 59 M | 92.60±24.5 | 86.63; 98.61 |  | 1 |  |  |  |  |  |  |  | |  | |  |  |
|  |  | 20 WT (F) | 97.65±19.1 | 87.36; 107.94 | 2-way ANOVA (GROUP*SEX) | - | 2 | 113 | 1.02 | .364 | .224 |  |  | |  | |  |  |
|  |  | 20 NTg (F) | 91.47±22.8 | 81.19; 101.76 |  | - |  |  |  |  |  |  |  | |  | |  |  |
|  |  | 20 Tg (F) | 80.05±17.3 | 69.76; 90.34 |  | - |  |  |  |  |  |  |  | |  | |  |  |
|  |  | 20 WT (M) | 93.33±24.6 | 83.04; 103.62 |  | - |  |  |  |  |  |  |  | |  | |  |  |
|  |  | 19 NTg (M) | 93.98±31.3 | 83.43; 104.53 |  | 1 |  |  |  |  |  |  |  | |  | |  |  |
|  | | 20 Tg (M) | 90.55±17.3 | 80.26; 100.83 |  | - |  |  |  |  |  |  |  | |  | |  |  |
|  | | 20 WT (F) | 97.65±19.1 | 88.06; 107.24 | 1-way ANOVA (F)  Bonferroni correction | - | 2 | 57 | 3.48 | .038* | .628 | WT vs NTg |  | | 38 | | .635 | -10.1; 22.5 |
|  |  | 20 NTg (F) | 91.47±22.8 | 81.89; 101.06 |  | - |  |  |  |  |  | WT vs Tg |  | | 38 | | .031* | 1.31; 33.9 |
|  |  | 20 Tg (F) | 80.05±17.3 | 70.46; 89.64 |  | - |  |  |  |  |  | NTg vs Tg |  | | 38 | | .219 | -4.87; 27.7 |
|  | | 20 WT (M) | 93.33±24.6 | 82.17; 104.50 | 1-way ANOVA (M)  Bonferroni correction | - | 2 | 56 | 0.11 | .899 | .065 |  |  | |  | |  |  |
|  |  | 19 NTg (M) | 93.98±31.3 | 82.52; 105.43 |  | 1 |  |  |  |  |  |  |  | |  | |  |  |
|  |  | 20 Tg ((M) | 90.55±17.3 | 79.38; 101.7 |  | - |  |  |  |  |  |  |  | |  | |  |  |
|  | **Social propinquity** | | | | | | | | | | | | | | | | | |
| **Measure** | | ***N* (pairs)** | **Mean ± SD** | **95% CI (lower; upper)** |  | **MV** | **d.f.** | **Residual** | **F** | **p** | **Effect**  **size** | **Post hoc tests (Multiple comparisons Tukey HSD)** | | | | | | |
|  |  |  |  |  |  |  |  |  |  |  |  |  | **t** | | **d.f.** | | **p** | **CI** |
| ***Latency to first share*** | | 12 WT | 88.5±86.0 | 36.7; 140.3 | 2-way ANOVA (GROUP)  Bonferroni correction | 8 | 2 | 39 | 0.16 | .854 | .065 |  |  | |  | |  |  |
|  |  | 17 NTg | 76.1±84.8 | 31.2; 120.9 |  | 3 |  |  |  |  |  |  |  | |  | |  |  |
|  |  | 16 Tg | 89.0±90.3 | 43.8; 134.3 |  | 4 |  |  |  |  |  |  |  | |  | |  |  |
|  |  | 24 F | 71.6±70.4 | 32.2; 108.2 | 2-way ANOVA (SEX) | 6 | 1 | 39 | 1.66 | .205 | .179 |  |  | |  | |  |  |
|  |  | 21 M | 97.9±99.1 | 59.4; 138.3 |  | 9 |  |  |  |  |  |  |  | |  | |  |  |
|  |  | 6 WT (F) | 63.0±89.4 | -10.3; 136.3 | 2-way ANOVA (GROUP*SEX) | 4 | 2 | 39 | 0.44 | .647 | .094 |  |  | |  | |  |  |
|  |  | 9 NTg (F) | 58.7±61.0 | -4.7; 122.2 |  | 1 |  |  |  |  |  |  |  | |  | |  |  |
|  |  | 9 Tg (F) | 88.8±69.4 | 29.0; 157.1 |  | 1 |  |  |  |  |  |  |  | |  | |  |  |
|  |  | 6 WT (M) | 114.0±81.9 | 40.7; 187.3 |  | 4 |  |  |  |  |  |  |  | |  | |  |  |
|  |  | 8 NTg (M) | 93.4±104.9 | 29.9; 156.8 |  | 2 |  |  |  |  |  |  |  | |  | |  |  |
|  | | 7 Tg (M) | 89.2±118.2 | 21.4; 157.1 |  | 3 |  |  |  |  |  |  |  | |  | |  |  |
|  | | 6 WT (F) | 63.0±89.4 | 1.4; 124.6 | 1-way ANOVA (F)  Bonferroni correction | 4 | 2 | 21 | 0.57 | .575 | .109 |  |  | |  | |  |  |
|  |  | 8 NTg (F) | 58.7±61.0 | 5.4; 112.0 |  | 2 |  |  |  |  |  |  |  | |  | |  |  |
|  |  | 9 Tg (F) | 88.8±69.4 | 38.6; 139.1 |  | 1 |  |  |  |  |  |  |  | |  | |  |  |
|  | | 6 WT (M) | 114.0±81.9 | 24.9; 203.1 | 1-way ANOVA (M)  Bonferroni correction | 4 | 2 | 18 | 0.16 | .852 | .064 |  |  | |  | |  |  |
|  |  | 8 NTg (M) | 93.4±104.9 | 16.2; 170.5 |  | 2 |  |  |  |  |  |  |  | |  | |  |  |
|  |  | 7 Tg ((M) | 89.2±118.2 | 6.7; 171.7 |  | 3 |  |  |  |  |  |  |  | |  | |  |  |
| ***Time vacant*** | | 12 WT | 20.8±19.0 | 14.3; 27.4 | 2-way ANOVA (GROUP)  Bonferroni correction | 8 | 2 | 42 | 5.75 | .006** | .668 | WT vs NTg | 1.91 | | 27 | | .150 | -2.2; 18.4 |
|  |  | 17 NTg | 12.7±11.1 | 7.1; 18.1 |  | 2 |  |  |  |  |  | WT vs Tg | 2.76 | | 28 | | .023* | 1.4; 21.8 |
|  |  | 18 Tg | 9.3±9.0 | 3.9; 14.6 |  | 2 |  |  |  |  |  | NTg vs Tg | 0.92 | | 33 | | .634 | -5.8; 12.7 |
|  |  | 24 F | 8.3±8.3 | 3.6; 13.2 | 2-way ANOVA (SEX) | 6 | 1 | 42 | 16.65 | <.001 *** | .926 |  |  | |  | |  |  |
|  |  | 24 M | 18.4±15.5 | 15.3; 24.8 |  | 6 |  |  |  |  |  |  |  | |  | |  |  |
|  |  | 6 WT (F) | 8.3±7.5 | -0.9; 17.6 | 2-way ANOVA (GROUP*SEX) | 4 | 2 | 42 | 5.34 | .009** | .626 |  |  | |  | |  |  |
|  |  | 9 NTg (F) | 10.4±10.7 | 2.4; 18.5 |  | 1 |  |  |  |  |  |  |  | |  | |  |  |
|  |  | 9 Tg (F) | 6.5±6.9 | -1.1; 14.1 |  | 1 |  |  |  |  |  |  |  | |  | |  |  |
|  |  | 6 WT (M) | 33.3±19.0 | 24.1; 42.6 |  | 4 |  |  |  |  |  |  |  | |  | |  |  |
|  |  | 9 NTg (M) | 14.8±11.6 | 7.2; 22.4 |  | 1 |  |  |  |  |  |  |  | |  | |  |  |
|  | | 9 Tg (M) | 18.4±15.5 | 4.5; 19.6 |  | 1 |  |  |  |  |  |  |  | |  | |  |  |
|  | | 6 WT (F) | 8.3±7.5 | 1.1; 15.6 | 1-way ANOVA (F)  Bonferroni correction | 4 | 2 | 21 | 0.49 | .619 | .113 |  |  | |  | |  |  |
|  |  | 8 NTg (F) | 10.4±10.7 | 4.1; 16.7 |  | 2 |  |  |  |  |  |  |  | |  | |  |  |
|  |  | 9 Tg (F) | 6.5±6.9 | 0.5, 12.4 |  | 1 |  |  |  |  |  |  |  | |  | |  |  |
|  |  | 6 WT (M) | 33.3±19.0 | 22.0; 44.7 | 1-way ANOVA (M)  Bonferroni correction | 4 | 2 | 21 | 7.53 | .003** | .763 | WT vs NTg | 2.63 | | 15 | | .039* | 0.8; 36.2 |
|  |  | 9 NTg (M) | 14.8±11.6 | 5.6; 24.1 |  | 1 |  |  |  |  |  | WT vs Tg | 3.03 | | 15 | | .017* | 3.6; 39.0 |
|  |  | 9 Tg (M) | 18.4±15.5 | 2.8; 21.3 |  | 1 |  |  |  |  |  | NTg vs Tg | 0.44 | | 18 | | .898 | -13.1; 18.6 |
| ***Duration of double occupan-cy*** | | 12 WT | 45.8±28.8 | 32.7; 59.0 | 2-way ANOVA (GROUP)  Bonferroni correction | 8 | 2 | 42 | 3.07 | .057 | .485 |  |  | |  | |  |  |
|  |  | 17 NTg | 64.2±22.8 | 53.6; 75.8 |  | 3 |  |  |  |  |  |  |  | |  | |  |  |
|  |  | 18 Tg | 60.2±27.8 | 49.4; 70.9 |  | 2 |  |  |  |  |  |  |  | |  | |  |  |
|  |  | 23 F | 70.3±16.6 | 60.8; 80.1 | 2-way ANOVA (SEX) | 7 | 1 | 42 | 18.53 | <.001 *** | .977 |  |  | |  | |  |  |
|  |  | 24 M | 46.2±29.6 | 33.9; 52.8 |  | 6 |  |  |  |  |  |  |  | |  | |  |  |
|  |  | 6 WT (F) | 70.8±15.6 | 52.2; 89.4 | 2-way ANOVA (GROUP*SEX) | 4 | 2 | 42 | 4.21 | .022* | .489 | WT vs NTg | 2.16 | | 27 | | .091 | -39.1; 2.3 |
|  |  | 8 NTg (F) | 72.9±15.3 | 56.8; 89.0 |  | 2 |  |  |  |  |  | WT vs Tg | 1.71 | | 28 | | .215 | 34.8; 6.1 |
|  |  | 9 Tg (F) | 67.6±19.7 | 52.4; 82.8 |  | 1 |  |  |  |  |  | NTg vs Tg | 0.53 | | 33 | | .858 | -14.5; 22.6 |
|  |  | 6 WT (M) | 20.8±8.7 | 2.2; 39.4 |  | 4 |  |  |  |  |  |  |  | |  | |  |  |
|  |  | 9 NTg (M) | 56.5±26.3 | 41.3; 71.7 |  | 1 |  |  |  |  |  |  |  | |  | |  |  |
|  | | 9 Tg (M) | 52.8±33.6 | 37.6; 68.0 |  | 1 |  |  |  |  |  |  |  | |  | |  |  |
|  | | 6 WT (F) | 70.8±15.6 | 56.1; 85.52 | 1-way ANOVA (F)  Bonferroni correction | 4 | 2 | 21 | 0.08 | .924 | .078 |  |  | |  | |  |  |
|  |  | 8 NTg (F) | 72.9±15.3 | 60.2; 85.7 |  | 2 |  |  |  |  |  |  |  | |  | |  |  |
|  |  | 9 Tg (F) | 67.6±19.7 | 55.6; 79.6 |  | 1 |  |  |  |  |  |  |  | |  | |  |  |
|  |  | 6 WT (M) | 20.8±8.7 | -1.8; 43.5 | 1-way ANOVA (M)  Bonferroni correction | 4 | 2 | 21 | 5.40 | .013* | .609 | WT vs NTg | 2.54 | | 15 | | .048* | -71.1; -0.2 |
|  |  | 9 NTg (M) | 56.5±26.3 | 38.0; 75.0 |  | 1 |  |  |  |  |  | WT vs Tg | 2.27 | | 15 | | .082 | -67.4; 3.5 |
|  |  | 9 Tg (M) | 52.8±33.6 | 34.3; 71.3 |  | 1 |  |  |  |  |  | NTg vs Tg | 0.29 | | 18 | | .953 | -28.0; 35.4 |
|  | **Exploratory reluctance test** | | | | | | | | | | | | | | | | | |
| **Measure** | | ***N* (animals)** | **Mean ± SD** | **95% CI (lower; upper)** |  | **MV** | **d.f.** | **Residual** | **F** | **p** | **Effect**  **size** | **Post hoc tests (Multiple comparisons Tukey HSD)** | | | | | | |
|  |  |  |  |  |  |  |  |  |  |  |  |  | **t** | | **d.f.** | | **p** | **CI** |
| ***Latency to first approach*** | | 39 WT | 9.5±6.6 | -8.6; 27.7 | 2-way ANOVA (GROUP)  Bonferroni correction | 1 | 2 | 113 | 3.59 | .031* | .651 | WT vs NTg |  | | 77 | | .802 | -38.8; 22.4 |
|  |  | 40 NTg | 17.7±24.3 | -0.2; 35.6 |  | - |  |  |  |  |  | WT vs Tg |  | | 77 | | .032* | -63.5; -2.4 |
|  |  | 40 Tg | 42.5±96.1 | 24.6; 60.4 |  | - |  |  |  |  |  | NTg vs Tg |  | | 78 | | .133 | -55.2; 5.6 |
|  |  | 60 F | 29.3±76.9 | 14.7; 43.9 | 2-way ANOVA (SEX) | - | 1 | 113 | 1.35 | .248 | .209 |  |  | |  | |  |  |
|  |  | 59 M | 17.3±30.8 | 2.4; 31.9 |  | 1 |  |  |  |  |  |  |  | |  | |  |  |
|  |  | 20 WT (F) | 8.9±6.9 | -16.4; 34.3 | 2-way ANOVA (GROUP*SEX) | - | 2 | 113 | 1.55 | .217 | .321 |  |  | |  | |  |  |
|  |  | 20 NTg (F) | 17.5±32.6 | -7.9; 42.8 |  | - |  |  |  |  |  |  |  | |  | |  |  |
|  |  | 20 Tg (F) | 61.5±128.4 | 36.2; 86.9 |  | - |  |  |  |  |  |  |  | |  | |  |  |
|  |  | 19 WT (M) | 10.2±6.5 | -15.8; 36.2 |  | 1 |  |  |  |  |  |  |  | |  | |  |  |
|  |  | 20 NTg (M) | 17.9±32.6 | -7.4; 43.3 |  | - |  |  |  |  |  |  |  | |  | |  |  |
|  | | 20 Tg (M) | 23.4±41.2 | -1.9; 48.8 |  | - |  |  |  |  |  |  |  | |  | |  |  |
|  | | 20 WT (F) | 8.9±6.9 | -24.5; 42.3 | 1-way ANOVA (F)  Bonferroni correction | - | 2 | 57 | 2.87 | .065 | .540 |  |  | |  | |  |  |
|  |  | 20 NTg (F) | 17.5±32.6 | -15.9; 50.8 |  | - |  |  |  |  |  |  |  | |  | |  |  |
|  |  | 20 Tg (F) | 61.5±128.4 | 28.2; 94.9 |  | - |  |  |  |  |  |  |  | |  | |  |  |
|  |  | 19 WT (M) | 10.2±6.5 | -4.0; 24.4 | 1-way ANOVA (M)  Bonferroni correction | 1 | 2 | 56 | 0.93 | .400 | .198 |  |  | |  | |  |  |
|  |  | 20 NTg (M) | 17.9±32.6 | 4.1; 31.8 |  | - |  |  |  |  |  |  |  | |  | |  |  |
|  |  | 20 Tg (M) | 23.4±41.2 | 9.6; 37.2 |  | - |  |  |  |  |  |  |  | |  | |  |  |
| ***Latency to first cross*** | | 39 WT | 102.3±136.5 | 51.9; 152.9 | 2-way ANOVA (GROUP)  Bonferroni correction | 1 | 2 | 113 | 1.98 | .143 | .402 |  |  | |  | |  |  |
|  |  | 40 NTg | 92.9±115.0 | 43.1; 142.8 |  | - |  |  |  |  |  |  |  | |  | |  |  |
|  |  | 40 Tg | 158.5±191.0 | 108.6; 208.3 |  | - |  |  |  |  |  |  |  | |  | |  |  |
|  |  | 60 F | 134.7±177.4 | 94.0; 175.4 | 2-way ANOVA (SEX) | - | 1 | 113 | 1.33 | .251 | .207 |  |  | |  | |  |  |
|  |  | 59 M | 101.1±141.4 | 60.1; 142.2 |  | 1 |  |  |  |  |  |  |  | |  | |  |  |
|  |  | 20 WT (F) | 99.0±138.8 | 28.5; 169.5 | 2-way ANOVA (GROUP*SEX) | - | 2 | 113 | 1.07 | .347 | .232 |  |  | |  | |  |  |
|  |  | 20 NTg (F) | 100.6±158.5 | 30.1; 171.1 |  | - |  |  |  |  |  |  |  | |  | |  |  |
|  |  | 20 Tg (F) | 204.6±213.7 | 134.1; 275.1 |  | - |  |  |  |  |  |  |  | |  | |  |  |
|  |  | 19 WT (M) | 105.9±137.7 | 33.5; 178.2 |  | 1 |  |  |  |  |  |  |  | |  | |  |  |
|  |  | 20 NTg (M) | 85.3±133.7 | 14.7; 155.8 |  | - |  |  |  |  |  |  |  | |  | |  |  |
|  | | 20 Tg (M) | 112.4±157.3 | 41.9; 182.9 |  | - |  |  |  |  |  |  |  | |  | |  |  |
|  | | 20 WT (F) | 99.0±138.8 | 21.4; 176.6 | 1-way ANOVA (F)  Bonferroni correction | - | 2 | 57 | 2.44 | .096 | .472 |  |  | |  | |  |  |
|  |  | 20 NTg (F) | 100.6±158.5 | 23.0; 178.2 |  | - |  |  |  |  |  |  |  | |  | |  |  |
|  |  | 20 Tg (F) | 204.6±213.7 | 127.0; 282.2 |  | - |  |  |  |  |  |  |  | |  | |  |  |
|  |  | 19 WT (M) | 105.9±137.7 | 40.0; 171.8 | 1-way ANOVA (M)  Bonferroni correction | 1 | 2 | 56 | 0.20 | .823 | .079 |  |  | |  | |  |  |
|  |  | 20 NTg (M) | 85.3±133.7 | 21.0; 149.5 |  | - |  |  |  |  |  |  |  | |  | |  |  |
|  |  | 20 Tg (M) | 112.4±157.3 | 48.1; 176.6 |  | - |  |  |  |  |  |  |  | |  | |  |  |
|  | **Lickometry** | | | | | | | | | | | | | | | | | |
| **Measure** | | ***N* (animals)** | **Mean ± SD** | **95% CI (lower; upper)** |  | **MV** | **d.f.** | **Residual** | **F** | **p** | **Effect**  **size** | **Post hoc tests (Multiple comparisons Tukey HSD)** | | | | | | |
|  |  |  |  |  |  |  |  |  |  |  |  |  | **t** | | **d.f.** | | **p** | **CI** |
| ***LCS*** | | 38 WT | 39.7±36.4 | 32.94; 46.55 | 3-way repeated measures ANOVA (GROUP)  Bonferroni correction | 2 | 2 | 102 | 4.64 | .012* | .771 | WT vs NTg | 1.79 | | 69 | | .177 | -2.94; 20.9 |
|  |  | 33 NTg | 31.0±24.3 | 23.67; 38.31 |  | 7 |  |  |  |  |  | WT vs Tg | 3.00 | | 73 | | .009** | 3.05; 26.2 |
|  |  | 37 Tg | 25.0±16.6 | 18.09; 31.86 |  | 3 |  |  |  |  |  | NTg vs Tg | 1.12 | | 68 | | .506 | -6.38; 17.7 |
|  |  | 51 F | 35.4±30.5 | 29.53; 41.31 | 3-way repeated measures ANOVA (SEX)  Bonferroni correction | 9 | 1 | 102 | 2.98 | .087 | .401 |  |  | |  | |  |  |
|  |  | 57 M | 28.4±24.7 | 22.83; 33.93 |  | 3 |  |  |  |  |  |  |  | |  | |  |  |
|  |  |  |  |  |  |  |  |  |  |  |  |  |  |  |  |  |  |  |
|  |  | 108 4% | 22.4±12.0 | 20.27; 24.62 | 3-way repeated measures ANOVA (CONC)  Bonferroni correction | 12 | 1 | 102 | 45.39 | <0.001 *** | 1.00 |  |  | |  | |  |  |
|  |  | 109 16% | 41.4±35.1 | 34.76; 47.94 |  | 11 |  |  |  |  |  |  |  | |  | |  |  |
|  |  |  |  |  |  |  |  |  |  |  |  |  |  |  |  |  |  |  |
|  | | 18 WT (F) | 43.4±39.5 | 33.53; 53.27 | 3-way repeated measure ANOVA (GROUP*SEX)  Bonferroni correction | 2 | 2 | 102 | 0.15 | .865 | .072 |  |  | |  | |  |  |
|  |  | 15 NTg (F) | 35.8±27.7 | 24.98; 46.60 |  | 5 |  |  |  |  |  |  |  | |  | |  |  |
|  |  | 18 Tg (F) | 27.1±18.8 | 17.20; 36.93 |  | 2 |  |  |  |  |  |  |  | |  | |  |  |
|  |  | 20 WT (M) | 36.1±33.4 | 26.73; 45.45 |  | - |  |  |  |  |  |  |  | |  | |  |  |
|  |  | 18 NTg (M) | 26.2±20.4 | 16.31; 36.05 |  | 2 |  |  |  |  |  |  |  | |  | |  |  |
|  |  | 19 Tg (M) | 22.9±14.2 | 13.26; 32.47 |  | 1 |  |  |  |  |  |  |  | |  | |  |  |
|  |  | 38 WT (4%) | 27.4±14.9 | 23.93; 31.25 | 3-way repeated measure ANOVA (GROUP*CONC)  Bonferroni correction | 2 | 2 | 102 | 1.36 | .260 | .288 | WT vs NTg (4%) | | 2.29 | | 69 | .072 | -0.39; 12.8 |
|  |  | 33 NTg (4%) | 21.3±10.6 | 17.45; 25.33 |  | 7 |  |  |  |  |  | WT vs Tg (4%) | | 3.52 | | 73 | .002** | 2.85; 15.6 |
|  |  | 37 Tg (4%) | 18.3±7.3 | 14.65; 22.06 |  | 3 |  |  |  |  |  | NTg vs Tg (4%) | | 1.12 | | 68 | .805 | -3.60; 9.67 |
|  |  | 38 WT (16%) | 51.7±46.4 | 40.81; 62.98 |  | 2 |  |  |  |  |  | WT vs NTg (16%) | | 1.38 | | 70 | .513 | -8.67; 31.3 |
|  |  | 33 NTg (16%) | 39.8±30.3 | 28.65; 52.51 |  | 7 |  |  |  |  |  | WT vs Tg (16%) | | 2.55 | | 73 | .036* | 0.96; 39.7 |
|  |  | 37 Tg (16%) | 31.5±20.4 | 20.35; 42.80 |  | 3 |  |  |  |  |  | NTg vs Tg (16%) | | 1.09 | | 69 | .835 | -29.1; 11.1 |
|  |  | 18 WT (F;4%) | 31.6±17.9 | 26.3; 36.9 | 3-way repeated measure ANOVA (GROUP*SEX*CONC)  Bonferroni correction | 2 | 2 | 102 | 0.79 | .456 | .182 |  |  | |  | |  |  |
|  |  | 15 NTg (F;4%) | 22.2±8.8 | 16.4; 28.0 |  | 5 |  |  |  |  |  |  |  | |  | |  |  |
|  |  | 18 Tg (F;4%) | 19.1±8.0 | 13.8; 24.4 |  | 2 |  |  |  |  |  |  |  | |  | |  |  |
|  |  | 20 WT (M;4%) | 23.6±10.9 | 18.6; 28.7 |  | - |  |  |  |  |  |  |  | |  | |  |  |
|  |  | 18 NTg (M;4%) | 20.6±12.1 | 15.3; 25.9 |  | 2 |  |  |  |  |  |  |  | |  | |  |  |
|  |  | 19 Tg (M;4%) | 17.1±6.8 | 12.5; 22.8 |  | 1 |  |  |  |  |  |  |  | |  | |  |  |
|  |  | 18 WT (F;16%) | 55.2±51.0 | 39.2; 71.3 |  | 2 |  |  |  |  |  |  |  | |  | |  |  |
|  |  | 15 NTg (F;16%) | 49.4±33.4 | 31.8; 67.0 |  | 5 |  |  |  |  |  |  |  | |  | |  |  |
|  |  | 18 Tg (F;16%) | 35.1±23.0 | 19.0; 51.2 |  | 2 |  |  |  |  |  |  |  | |  | |  |  |
|  |  | 20 WT (M;16%) | 48.6±43.0 | 33.3; 63.8 |  | - |  |  |  |  |  |  |  | |  | |  |  |
|  |  | 18 NTg (M;16%) | 31.7±25.7 | 15.6; 47.8 |  | 2 |  |  |  |  |  |  |  | |  | |  |  |
|  |  | 19 Tg (M;16%) | 28.1±17.6 | 12.4; 43.7 |  | 1 |  |  |  |  |  |  |  | |  | |  |  |
| ***Change in LCS*** | | 38 WT | 1.0±1.5 | 0.6; 1.4 | 2-way ANOVA (GROUP)  Bonferroni correction | 2 | 2 | 102 | 1.36 | .261 | .287 |  |  | |  | |  |  |
|  |  | 33 NTg | 0.7±1.2 | 0.4; 1.2 |  | 7 |  |  |  |  |  |  |  | |  | |  |  |
|  |  | 37 Tg | 0.6±0.8 | 0.2; 0.9 |  | 3 |  |  |  |  |  |  |  | |  | |  |  |
|  |  | 51 F | 0.9±1.2 | 0.6; 1.3 | 2-way ANOVA (SEX) | 9 | 1 | 102 | 1.49 | .226 | .227 |  |  | |  | |  |  |
|  |  | 57 M | 0.7±1.2 | 0.3; 1.0 |  | 3 |  |  |  |  |  |  |  | |  | |  |  |
|  |  | 18 WT (F) | 1.0±1.6 | 0.4; 1.6 | 2-way ANOVA (GROUP*SEX) | 2 | 2 | 102 | 0.79 | .456 | .182 |  |  | |  | |  |  |
|  |  | 15 NTg (F) | 1.1±1.2 | 0.5; 1.8 |  | 5 |  |  |  |  |  |  |  | |  | |  |  |
|  |  | 18 Tg (F) | 0.7±0.8 | 0.1; 1.2 |  | 2 |  |  |  |  |  |  |  | |  | |  |  |
|  |  | 20 WT (M) | 1.0±1.5 | 0.5; 1.6 |  | - |  |  |  |  |  |  |  | |  | |  |  |
|  |  | 18 NTg (M) | 0.5±1.1 | -0.1; 1.0 |  | 2 |  |  |  |  |  |  |  | |  | |  |  |
|  | | 19 Tg (M) | 0.4±0.7 | -0.1; 1.0 |  | 1 |  |  |  |  |  |  |  | |  | |  |  |
|  | | 18 WT (F) | 1.0±1.6 | 0.4; 1.6 | 1-way ANOVA (F)  Bonferroni correction | 2 | 2 | 48 | 0.61 | .549 | .145 |  |  | |  | |  |  |
|  |  | 15 NTg (F) | 1.1±1.2 | 0.5; 1.8 |  | 5 |  |  |  |  |  |  |  | |  | |  |  |
|  |  | 18 Tg (F) | 0.7±0.8 | 0.1; 1.3 |  | 2 |  |  |  |  |  |  |  | |  | |  |  |
|  |  | 20 WT (M) | 1.0±1.5 | 0.5; 1.6 | 1-way ANOVA (M)  Bonferroni correction | - | 2 | 54 | 1.66 | .199 | .335 |  |  | |  | |  |  |
|  |  | 18 NTg (M) | 0.5±1.1 | -0.1; 1.0 |  | 2 |  |  |  |  |  |  |  | |  | |  |  |
|  |  | 19 Tg (M) | 0.4±0.7 | -0.1; 1.0 |  | 1 |  |  |  |  |  |  |  | |  | |  |  |
| ***Total number of licks*** | | 38 WT | 858±426 | 728; 986 | 2-way ANOVA (GROUP)  Bonferroni correction | 2 | 2 | 103 | 0.25 | .782 | .088 |  |  | |  | |  |  |
|  |  | 34 NTg | 813±379 | 668; 940 |  | 6 |  |  |  |  |  |  |  | |  | |  |  |
|  |  | 37 Tg | 799±388 | 667; 929 |  | 3 |  |  |  |  |  |  |  | |  | |  |  |
|  |  | 51 F | 772±406 | 657; 880 | 2-way ANOVA (SEX) | 9 | 1 | 103 | 1.69 | .196 | .252 |  |  | |  | |  |  |
|  |  | 58 M | 869±385 | 765; 973 |  | 2 |  |  |  |  |  |  |  | |  | |  |  |
|  |  | 18 WT (F) | 832±423 | 645; 1019 | 2-way ANOVA (GROUP*SEX) | 2 | 2 | 103 | 0.30 | .742 | .096 |  |  | |  | |  |  |
|  |  | 15 NTg (F) | 710±380 | 505; 915 |  | 5 |  |  |  |  |  |  |  | |  | |  |  |
|  |  | 18 Tg (F) | 765±424 | 578; 952 |  | 2 |  |  |  |  |  |  |  | |  | |  |  |
|  |  | 20 WT (M) | 881±439 | 704; 1059 |  | - |  |  |  |  |  |  |  | |  | |  |  |
|  |  | 19 NTg (M) | 895±367 | 713; 1077 |  | 1 |  |  |  |  |  |  |  | |  | |  |  |
|  | | 19 Tg (M) | 931±359 | 649; 1013 |  | 1 |  |  |  |  |  |  |  | |  | |  |  |
|  | | 18 WT (F) | 832±423 | 637; 1027 | 1-way ANOVA (F)  Bonferroni correction | 2 | 2 | 48 | 0.37 | .695 | .106 |  |  | |  | |  |  |
|  |  | 15 NTg (F) | 710±380 | 496; 923 |  | 5 |  |  |  |  |  |  |  | |  | |  |  |
|  |  | 18 Tg (F) | 765±424 | 570; 960 |  | 2 |  |  |  |  |  |  |  | |  | |  |  |
|  |  | 20 WT (M) | 881±439 | 706; 1056 | 1-way ANOVA (M)  Bonferroni correction | - | 2 | 55 | 0.14 | .868 | .071 |  |  | |  | |  |  |
|  |  | 19 NTg (M) | 895±367 | 715; 1075 |  | 1 |  |  |  |  |  |  |  | |  | |  |  |
|  |  | 19 Tg (M) | 931±359 | 651; 1011 |  | 1 |  |  |  |  |  |  |  | |  | |  |  |
|  | **Fecal corticosterone metabolites** | | | | | | | | | | | | | | | | | |
| **Measure** | | ***N* (animals)** | **Mean ± SD** | **95% CI (lower; upper)** |  | **MV** | **d.f.** | **Residual** | **F** | **p** | **Effect**  **size** | **Post hoc tests (Multiple comparisons Tukey HSD)** | | | | | | |
|  |  |  |  |  |  |  |  |  |  |  |  |  | **t** | | **d.f.** | | **p** | **CI** |
| ***Baseline*** | | 23 WT | 42.5±20.6 | 36.9; 49.0 | 2-way ANOVA (GROUP)  Bonferroni correction | 1 | 2 | 64 | 4.60 | .014* | .760 | WT vs NTg | 2.49 | | 44 | | .041* | 0.37; 20.9 |
|  |  | 23 NTg | 31.9±14.3 | 26.2; 38.2 |  | 1 |  |  |  |  |  | WT vs Tg | 2.63 | | 45 | | .029* | 0.96; 21.2 |
|  |  | 24 Tg | 31.4±11.4 | 25.5; 37.4 |  | - |  |  |  |  |  | NTg vs Tg | 0.11 | | 45 | | .993 | -9.66; 10.6 |
|  |  | 34 F | 41.5±19.9 | 36.8; 46.7 | 2-way ANOVA (SEX) | 2 | 1 | 64 | 12.95 | .001*** | .943 |  |  | |  | |  |  |
|  |  | 36 M | 29.3±9.1 | 24.5; 34.1 |  | - |  |  |  |  |  |  |  | |  | |  |  |
|  |  | 11 WT (F) | 53.1±23.9 | 44.4; 61.8 | 2-way ANOVA (GROUP*SEX) | 1 | 2 | 64 | 1.70 | .190 | .345 |  |  | |  | |  |  |
|  |  | 11 NTg (F) | 38.4±16.5 | 29.7; 47.2 |  | 1 |  |  |  |  |  |  |  | |  | |  |  |
|  |  | 12 Tg (F) | 33.8±14.7 | 25.4; 42.1 |  | - |  |  |  |  |  |  |  | |  | |  |  |
|  |  | 12 WT (M) | 32.9±10.7 | 24.5; 41.2 |  | - |  |  |  |  |  |  |  | |  | |  |  |
|  |  | 12 NTg (M) | 26.0±9.1 | 17.6; 34.3 |  | - |  |  |  |  |  |  |  | |  | |  |  |
|  | | 12 Tg (M) | 29.1±6.4 | 20.8; 37.5 |  | - |  |  |  |  |  |  |  | |  | |  |  |
|  | | 11 WT (F) | 53.1±23.9 | 41.6; 64.6 | 1-way ANOVA (F)  Bonferroni correction | 1 | 2 | 31 | 3.30 | .05* | .583 | WT vs NTg | 1.84 | | 20 | | .173 | -4.9; 34.3 |
|  |  | 11 NTg (F) | 38.4±16.5 | 27.0; 50.0 |  | 1 |  |  |  |  |  | WT vs Tg | 2.48 | | 21 | | .048* | 0.15; 38.5 |
|  |  | 12 Tg (F) | 33.8±14.7 | 22.8; 44.8 |  | - |  |  |  |  |  | NTg vs Tg | 0.60 | | 21 | | .821 | -14.5; 23.9 |
|  | | 12 WT (M) | 32.9±10.7 | 27.6; 38.1 | 1-way ANOVA (M)  Bonferroni correction | - | 2 | 33 | 1.81 | .179 | .351 |  |  | |  | |  |  |
|  |  | 12 NTg (M) | 26.0±9.1 | 20.7; 31.2 |  | - |  |  |  |  |  |  |  | |  | |  |  |
|  |  | 12 Tg (M) | 29.1±6.4 | 23.9; 34.3 |  | - |  |  |  |  |  |  |  | |  | |  |  |
| ***Stressed*** | | 23 WT | 50.7±30.5 | 41.2; 59.4 | 2-way ANOVA (GROUP)  Bonferroni correction | 1 | 2 | 64 | 1.21 | .306 | .255 |  |  | |  | |  |  |
|  |  | 23 NTg | 40.7±17.5 | 31.9; 49.6 |  | 1 |  |  |  |  |  |  |  | |  | |  |  |
|  |  | 24 Tg | 43.5±15.3 | 34.2; 52.3 |  | - |  |  |  |  |  |  |  | |  | |  |  |
|  |  | 34 F | 39.7±18.1 | 32.2; 47.1 | 2-way ANOVA (SEX) | 2 | 1 | 64 | 3.85 | .054 | .489 |  |  | |  | |  |  |
|  |  | 36 M | 49.9±24.6 | 42.6; 57.1 |  | - |  |  |  |  |  |  |  | |  | |  |  |
|  |  | 11 WT (F) | 41.4±18.8 | 28.4; 54.5 | 2-way ANOVA (GROUP*SEX) | 1 | 2 | 64 | 0.71 | .495 | .165 |  |  | |  | |  |  |
|  |  | 11 NTg (F) | 39.5±20.6 | 26.9; 52.0 |  | 1 |  |  |  |  |  |  |  | |  | |  |  |
|  |  | 12 Tg (F) | 38.1±16.0 | 25.0; 51.2 |  | - |  |  |  |  |  |  |  | |  | |  |  |
|  |  | 12 WT (M) | 59.2±37.2 | 46.6; 71.7 |  | - |  |  |  |  |  |  |  | |  | |  |  |
|  |  | 12 NTg (M) | 42.0±14.5 | 29.5; 54.6 |  | - |  |  |  |  |  |  |  | |  | |  |  |
|  | | 12 Tg (M) | 48.4±13.3 | 35.9; 61.0 |  | - |  |  |  |  |  |  |  | |  | |  |  |
|  | | 11 WT (F) | 41.4±18.8 | 30.0; 52.9 | 1-way ANOVA (F)  Bonferroni correction | 1 | 2 | 31 | 0.09 | .916 | .062 |  |  | |  | |  |  |
|  |  | 11 NTg (F) | 39.5±20.6 | 28.5; 50.4 |  | 1 |  |  |  |  |  |  |  | |  | |  |  |
|  |  | 12 Tg (F) | 38.1±16.0 | 26.7; 49.6 |  | - |  |  |  |  |  |  |  | |  | |  |  |
|  |  | 12 WT (M) | 59.2±37.2 | 44.9; 73.4 | 1-way ANOVA (M)  Bonferroni correction | - | 2 | 33 | 1.52 | .084 | .300 |  |  | |  | |  |  |
|  |  | 12 NTg (M) | 42.0±14.5 | 27.8; 56.3 |  | - |  |  |  |  |  |  |  | |  | |  |  |
|  |  | 12 Tg (M) | 48.4±13.3 | 34.2; 62.7 |  | - |  |  |  |  |  |  |  | |  | |  |  |
| ***Change*** | | 23 WT | 159±137 | 102; 211 | 2-way ANOVA (GROUP)  Bonferroni correction | 1 | 2 | 63 | 0.22 | .801 | .083 |  |  | |  | |  |  |
|  |  | 23 NTg | 153±91 | 97; 206 |  | 1 |  |  |  |  |  |  |  | |  | |  |  |
|  |  | 23 Tg | 176±159 | 121; 230 |  | 1 |  |  |  |  |  |  |  | |  | |  |  |
|  |  | 33 F | 138±150 | 92; 183 | 2-way ANOVA (SEX) | 3 | 1 | 63 | 2.28 | .136 | .319 |  |  | |  | |  |  |
|  |  | 36 M | 185±107 | 142; 229 |  | - |  |  |  |  |  |  |  | |  | |  |  |
|  |  | 11 WT (F) | 109±96 | 30; 187 | 2-way ANOVA (GROUP*SEX) | 1 | 2 | 63 | 0.90 | .413 | .198 |  |  | |  | |  |  |
|  |  | 11 NTg (F) | 124±84 | 46; 203 |  | 1 |  |  |  |  |  |  |  | |  | |  |  |
|  |  | 11 Tg (F) | 180±229 | 101; 258 |  | 1 |  |  |  |  |  |  |  | |  | |  |  |
|  |  | 12 WT (M) | 104±156 | 129; 280 |  | - |  |  |  |  |  |  |  | |  | |  |  |
|  |  | 12 NTg (M) | 179±93 | 103; 254 |  | - |  |  |  |  |  |  |  | |  | |  |  |
|  | | 12 Tg (M) | 172±52 | 97; 248 |  | - |  |  |  |  |  |  |  | |  | |  |  |
|  | | 11 WT (F) | 109±96 | 16; 202 | 1-way ANOVA (F)  Bonferroni correction | 1 | 2 | 30 | 0.67 | .521 | .151 |  |  | |  | |  |  |
|  |  | 11 NTg (F) | 124±84 | 31; 218 |  | 1 |  |  |  |  |  |  |  | |  | |  |  |
|  |  | 11 Tg (F) | 180±229 | 86; 273 |  | 1 |  |  |  |  |  |  |  | |  | |  |  |
|  |  | 12 WT (M) | 204±156 | 140; 268 | 1-way ANOVA (M)  Bonferroni correction | - | 2 | 33 | 0.29 | .751 | .092 |  |  | |  | |  |  |
|  |  | 12 NTg (M) | 179±93 | 115; 243 |  | - |  |  |  |  |  |  |  | |  | |  |  |
|  |  | 12 Tg (M) | 172±52 | 108; 236 |  | - |  |  |  |  |  |  |  | |  | |  |  |
|  | **Open field** | | | | | | | | | | | | | | | | | |
| **Measure** | | ***N* (animals)** | **Mean ± SD** | **95% CI (lower; upper)** |  | **MV** | **d.f.** | **Residual** | **F** | **p** | **Effect**  **size** | **Post hoc tests (Multiple comparisons Tukey HSD)** | | | | | | |
|  |  |  |  |  |  |  |  |  |  |  |  |  | **t** | | **d.f.** | | **p** | **CI** |
| ***Distance moved*** | | 24 WT | 56.3±22.7 | 48.7; 64.0 | 2-way ANOVA (GROUP)  Bonferroni correction | - | 2 | 66 | 0.00 | .998 | .050 |  |  | |  | |  |  |
|  |  | 24 NTg | 56.6±18.5 | 48.9; 64.2 |  | - |  |  |  |  |  |  |  | |  | |  |  |
|  |  | 24 Tg | 56.7±13.2 | 49.0; 64.4 |  | - |  |  |  |  |  |  |  | |  | |  |  |
|  |  | 36 F | 55.9±19.5 | 49.7; 62.1 | 2-way ANOVA (SEX) | - | 1 | 66 | 0.08 | .778 | .059 |  |  | |  | |  |  |
|  |  | 36 M | 57.2±17.3 | 50.9; 63.4 |  | - |  |  |  |  |  |  |  | |  | |  |  |
|  |  | 12 WT (F) | 59.0±26.5 | 48.1; 69.8 | 2-way ANOVA (GROUP*SEX) | - | 2 | 66 | 0.55 | .581 | .137 |  |  | |  | |  |  |
|  |  | 12 NTg (F) | 54.0±16.2 | 43.1; 64.8 |  | - |  |  |  |  |  |  |  | |  | |  |  |
|  |  | 12 Tg (F) | 54.8±15.1 | 44.0; 65.7 |  | - |  |  |  |  |  |  |  | |  | |  |  |
|  |  | 12 WT (M) | 53.7±19.1 | 42.9; 64.6 |  | - |  |  |  |  |  |  |  | |  | |  |  |
|  |  | 12 NTg (M) | 59.3±21.0 | 48.4; 70.1 |  | - |  |  |  |  |  |  |  | |  | |  |  |
|  | | 12 Tg (M) | 58.6±11.4 | 47.7; 69.4 |  | - |  |  |  |  |  |  |  | |  | |  |  |
|  | | 12 WT (F) | 59.0±26.5 | 47.3; 70.7 | 1-way ANOVA (F)  Bonferroni correction | - | 2 | 33 | 0.22 | .808 | .081 |  |  | |  | |  |  |
|  |  | 12 NTg (F) | 54.0±16.2 | 42.3; 65.7 |  | - |  |  |  |  |  |  |  | |  | |  |  |
|  |  | 12 Tg (F) | 54.8±15.1 | 43.1; 66.5 |  | - |  |  |  |  |  |  |  | |  | |  |  |
|  | | 12 WT (M) | 53.7±19.1 | 43.4; 64.1 | 1-way ANOVA (M)  Bonferroni correction | - | 2 | 33 | 0.35 | .706 | .102 |  |  | |  | |  |  |
|  |  | 12 NTg (M) | 59.3±21.0 | 48.9; 69.6 |  | - |  |  |  |  |  |  |  | |  | |  |  |
|  |  | 12 Tg (M) | 58.6±11.4 | 48.2; 68.9 |  | - |  |  |  |  |  |  |  | |  | |  |  |
| ***Moving time*** | | 24 WT | 512±153 | 461; 564 | 2-way ANOVA (GROUP)  Bonferroni correction | - | 2 | 66 | 0.32 | .724 | .100 |  |  | |  | |  |  |
|  |  | 24 NTg | 519±123 | 468; 571 |  | - |  |  |  |  |  |  |  | |  | |  |  |
|  |  | 24 Tg | 541±98 | 489; 593 |  | - |  |  |  |  |  |  |  | |  | |  |  |
|  |  | 36 F | 507±120 | 465; 549 | 2-way ANOVA (SEX) | - | 1 | 66 | 1.30 | .258 | .203 |  |  | |  | |  |  |
|  |  | 36 M | 541±130 | 499; 584 |  | - |  |  |  |  |  |  |  | |  | |  |  |
|  |  | 12 WT (F) | 518±158 | 444; 591 | 2-way ANOVA (GROUP*SEX) | - | 2 | 66 | 0.57 | .568 | .141 |  |  | |  | |  |  |
|  |  | 12 NTg (F) | 488±93 | 415; 562 |  | - |  |  |  |  |  |  |  | |  | |  |  |
|  |  | 12 Tg (F) | 515±108 | 442; 588 |  | - |  |  |  |  |  |  |  | |  | |  |  |
|  |  | 12 WT (M) | 507±154 | 434; 580 |  | - |  |  |  |  |  |  |  | |  | |  |  |
|  |  | 12 NTg (M) | 550±145 | 477; 624 |  | - |  |  |  |  |  |  |  | |  | |  |  |
|  | | 12 Tg (M) | 566±83 | 493; 640 |  | - |  |  |  |  |  |  |  | |  | |  |  |
|  | | 12 WT (F) | 518±158 | 445; 590 | 1-way ANOVA (F)  Bonferroni correction | - | 2 | 33 | 0.21 | .813 | .080 |  |  | |  | |  |  |
|  |  | 12 NTg (F) | 488±93 | 416; 561 |  | - |  |  |  |  |  |  |  | |  | |  |  |
|  |  | 12 Tg (F) | 515±108 | 443; 587 |  | - |  |  |  |  |  |  |  | |  | |  |  |
|  |  | 12 WT (M) | 507±154 | 430; 584 | 1-way ANOVA (M)  Bonferroni correction | - | 2 | 33 | 0.66 | .524 | .151 |  |  | |  | |  |  |
|  |  | 12 NTg (M) | 550±145 | 473; 627 |  | - |  |  |  |  |  |  |  | |  | |  |  |
|  |  | 12 Tg (M) | 566±83 | 489; 643 |  | - |  |  |  |  |  |  |  | |  | |  |  |
| ***Velocity*** | | 24 WT | 6.3±2.5 | 5.4; 7.1 | 2-way ANOVA (GROUP)  Bonferroni correction | - | 2 | 66 | 0.00 | .998 | .050 |  |  | |  | |  |  |
|  |  | 24 NTg | 6.3±2.1 | 5.4; 7.1 |  | - |  |  |  |  |  |  |  | |  | |  |  |
|  |  | 24 Tg | 6.3±1.5 | 5.4; 7.2 |  | - |  |  |  |  |  |  |  | |  | |  |  |
|  |  | 36 F | 6.2±2.2 | 5.5; 6.9 | 2-way ANOVA (SEX) | - | 1 | 66 | 0.08 | .778 | .059 |  |  | |  | |  |  |
|  |  | 36 M | 6.4±1.9 | 5.7; 7.1 |  | - |  |  |  |  |  |  |  | |  | |  |  |
|  |  | 12 WT (F) | 6.6±2.9 | 5.3; 7.8 | 2-way ANOVA (GROUP*SEX) | - | 2 | 66 | 0.55 | .581 | .137 |  |  | |  | |  |  |
|  |  | 12 NTg (F) | 6.0±1.8 | 4.8; 7.2 |  | - |  |  |  |  |  |  |  | |  | |  |  |
|  |  | 12 Tg (F) | 6.1±1.7 | 4.9; 7.3 |  | - |  |  |  |  |  |  |  | |  | |  |  |
|  |  | 12 WT (M) | 6.0±2.1 | 4.8; 7.2 |  | - |  |  |  |  |  |  |  | |  | |  |  |
|  |  | 12 NTg (M) | 6.6±2.3 | 5.4; 7.8 |  | - |  |  |  |  |  |  |  | |  | |  |  |
|  | | 12 Tg (M) | 6.5±1.3 | 5.3; 7.7 |  | - |  |  |  |  |  |  |  | |  | |  |  |
|  | | 12 WT (F) | 6.6±2.9 | 5.3; 7.9 | 1-way ANOVA (F)  Bonferroni correction | - | 2 | 33 | 0.21 | .808 | .081 |  |  | |  | |  |  |
|  |  | 12 NTg (F) | 6.0±1.8 | 4.7; 7.3 |  | - |  |  |  |  |  |  |  | |  | |  |  |
|  |  | 12 Tg (F) | 6.1±1.7 | 4.8; 7.4 |  | - |  |  |  |  |  |  |  | |  | |  |  |
|  |  | 12 WT (M) | 6.0±2.1 | 4.8; 7.1 | 1-way ANOVA (M)  Bonferroni correction | - | 2 | 33 | 0.35 | .706 | .102 |  |  | |  | |  |  |
|  |  | 12 NTg (M) | 6.6±2.3 | 5.4; 7.7 |  | - |  |  |  |  |  |  |  | |  | |  |  |
|  |  | 12 Tg (M) | 6.5±1.3 | 5.4; 7.7 |  | - |  |  |  |  |  |  |  | |  | |  |  |
|  | **Locomotor activity** | | | | | | | | | | | | | | | | | |
| **Measure** | | ***N* (animals)** | **Mean ± SD** | **95% CI (lower; upper)** |  | **MV** | **d.f.** | **Residual** | **F** | **p** | **Effect**  **size** | **Post hoc tests (Multiple comparisons Tukey HSD)** | | | | | | |
|  |  |  |  |  |  |  |  |  |  |  |  |  | **t** | | **d.f.** | | **p** | **CI** |
| ***Dark period activity*** | | 62 WT | 646±271 | 574; 714 | 2-way ANOVA (GROUP)  Bonferroni correction | 2 | 2 | 177 | 0.26 | .768 | .091 |  |  | |  | |  |  |
|  |  | 58 NTg | 648±273 | 560; 705 |  | 6 |  |  |  |  |  |  |  | |  | |  |  |
|  |  | 63 Tg | 667±306 | 599; 737 |  | 1 |  |  |  |  |  |  |  | |  | |  |  |
|  |  | 89 F | 585±255 | 525; 642 | 2-way ANOVA (SEX) | 7 | 1 | 177 | 9.88 | .002** | .878 |  |  | |  | |  |  |
|  |  | 94 M | 713±295 | 656; 770 |  | 2 |  |  |  |  |  |  |  | |  | |  |  |
|  |  | 30 WT (F) | 581±241 | 480; 681 | 2-way ANOVA (GROUP*SEX) | 2 | 2 | 177 | 0.17 | .842 | .076 |  |  | |  | |  |  |
|  |  | 27 NTg (F) | 552±232 | 446; 657 |  | 5 |  |  |  |  |  |  |  | |  | |  |  |
|  |  | 32 Tg (F) | 617±288 | 520; 715 |  | - |  |  |  |  |  |  |  | |  | |  |  |
|  |  | 32 WT (M) | 707±287 | 610; 804 |  | - |  |  |  |  |  |  |  | |  | |  |  |
|  |  | 31 NTg (M) | 713±286 | 614; 812 |  | 1 |  |  |  |  |  |  |  | |  | |  |  |
|  | | 31 Tg (M) | 719±320 | 620; 818 |  | 1 |  |  |  |  |  |  |  | |  | |  |  |
|  | | 30 WT (F) | 581±241 | 488; 674 | 1-way ANOVA (F)  Bonferroni correction | 2 | 2 | 86 | 0.49 | .616 | .128 |  |  | |  | |  |  |
|  |  | 27 NTg (F) | 552±232 | 453; 650 |  | 5 |  |  |  |  |  |  |  | |  | |  |  |
|  |  | 32 Tg (F) | 617±288 | 527; 707 |  | - |  |  |  |  |  |  |  | |  | |  |  |
|  | | 32 WT (M) | 707±287 | 602; 812 | 1-way ANOVA (M)  Bonferroni correction | - | 2 | 91 | 0.01 | .988 | .052 |  |  | |  | |  |  |
|  |  | 31 NTg (M) | 713±286 | 606; 819 |  | 1 |  |  |  |  |  |  |  | |  | |  |  |
|  |  | 31 Tg (M) | 719±320 | 613; 825 |  | 1 |  |  |  |  |  |  |  | |  | |  |  |
| ***Light period activity*** | | 62 WT | 251±143 | 215; 288 | 2-way ANOVA (GROUP)  Bonferroni correction | 2 | 2 | 177 | 0.36 | .697 | .107 |  |  | |  | |  |  |
|  |  | 58 NTg | 269±149 | 230; 305 |  | 6 |  |  |  |  |  |  |  | |  | |  |  |
|  |  | 63 Tg | 272±147 | 236; 308 |  | 1 |  |  |  |  |  |  |  | |  | |  |  |
|  |  | 89 F | 238±134 | 208; 268 | 2-way ANOVA (SEX) | 7 | 1 | 177 | 5.84 | .017* | .671 |  |  | |  | |  |  |
|  |  | 94 M | 289±152 | 260; 319 |  | 2 |  |  |  |  |  |  |  | |  | |  |  |
|  |  | 30 WT (F) | 242±140 | 190; 294 | 2-way ANOVA (GROUP*SEX) | 2 | 2 | 177 | 0.75 | .476 | .175 |  |  | |  | |  |  |
|  |  | 27 NTg (F) | 240±120 | 185; 295 |  | 5 |  |  |  |  |  |  |  | |  | |  |  |
|  |  | 32 Tg (F) | 232±145 | 181; 282 |  | - |  |  |  |  |  |  |  | |  | |  |  |
|  |  | 32 WT (M) | 261±143 | 210; 311 |  | - |  |  |  |  |  |  |  | |  | |  |  |
|  |  | 31 NTg (M) | 295±149 | 244; 346 |  | 1 |  |  |  |  |  |  |  | |  | |  |  |
|  | | 31 Tg (M) | 313±140 | 262; 364 |  | 1 |  |  |  |  |  |  |  | |  | |  |  |
|  | | 30 WT (F) | 242±140 | 193; 291 | 1-way ANOVA (F)  Bonferroni correction | 2 | 2 | 86 | 0.05 | .949 | .058 |  |  | |  | |  |  |
|  |  | 27 NTg (F) | 240±120 | 188; 292 |  | 5 |  |  |  |  |  |  |  | |  | |  |  |
|  |  | 32 Tg (F) | 232±145 | 184; 279 |  | - |  |  |  |  |  |  |  | |  | |  |  |
|  |  | 32 WT (M) | 261±143 | 207; 314 | 1-way ANOVA (M)  Bonferroni correction | - | 2 | 91 | 0.97 | .383 | .214 |  |  | |  | |  |  |
|  |  | 31 NTg (M) | 295±149 | 241; 349 |  | 1 |  |  |  |  |  |  |  | |  | |  |  |
|  |  | 31 Tg (M) | 313±140 | 259; 367 |  | 1 |  |  |  |  |  |  |  | |  | |  |  |
